# A Protecting‐Group‐Free Synthesis of (−)‐Salvinorin A

**DOI:** 10.1002/chem.202100560

**Published:** 2021-05-02

**Authors:** Patrick Zimdars, Yuzhou Wang, Peter Metz

**Affiliations:** ^1^ Fakultät Chemie und Lebensmittelchemie Organische Chemie I Technische Universität Dresden Bergstraße 66 01069 Dresden Germany

**Keywords:** Asymmetric catalysis, Cycloaddition, Mitsunobu reaction, Terpenoids, Total synthesis

## Abstract

A concise enantioselective total synthesis of the neoclerodane diterpene (−)‐salvinorin A is reported. The stereogenic center at C‐12 was installed by catalytic asymmetric propargylation with excellent enantioselectivity, and the remaining six stereogenic centers were set up highly diastereoselectively under substrate control. As for our previous synthesis of racemic salvinorin A, two intramolecular Diels‐Alder reactions were applied to generate the tricyclic core. A chemoselective Mitsunobu inversion of a *syn* 1,2‐diol allowed for further streamlining of the original reaction sequence by two steps. Overall, (−)‐salvinorin A was synthesized in only 16 steps starting from 3‐furaldehyde with 1.4 % total yield. Furthermore, an alternative intramolecular Diels‐Alder strategy employing a 2‐bromo‐1,3‐diene moiety was investigated.

## Graphical abstract

**A divine sage**: The potent KOR agonist (−)‐salvinorin A was synthesized in only sixteen steps by modification of our previous route to the racemic title compound. Transition to asymmetric synthesis succeeded by a highly enantioselective propargylation to install the stereogenic center at C‐12. Shortening of the original reaction sequence by two steps was finally achieved using a chemoselective Mitsunobu esterification of a cyclic *syn* 1,2‐diol. Furthermore, an alternative intramolecular Diels‐Alder strategy employing a 2‐bromo‐1,3‐diene moiety was investigated.

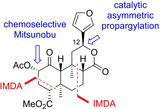

## Introduction

(−)‐Salvinorin A (**1**), a neoclerodane diterpene isolated from the leaves of the Mexican medicinal plant *Salvia divinorum*,^[1]^ is a potent and highly selective κ opioid receptor (KOR) agonist.^[2]^ Smoking of microgram amounts of **1** leads to hallucinogenic perceptions, and the leaves of *Salvia divinorum* have been used over centuries for spiritual practices by indigenous people in Mexico.^[3]^ Today, the diterpene **1** is considered to be a promising lead for the development of drugs against disorders of the central nervous system, such as depression, pain, and drug addiction.[^3^, ^4^] Thus, **1** represents an attractive synthetic target. To this date, four asymmetric total syntheses^[5]^ of (−)‐salvinorin A (**1**) have been developed by the groups of Evans^[6a]^ (2007), Hagiwara[^6b^, ^6c^] (2008 and 2009), and Forsyth^[6d]^ (2016). Moreover, further synthetic studies towards **1** ^[7]^ and extensive investigations on hundreds of analogues prepared by chemical modification of the natural product **1** have been reported.^[3]^ An exciting novel research direction focusses on the rapid generation of designed KOR agonistic analogues of **1** by total synthesis.[^5^, ^8^]

**Scheme 1:**
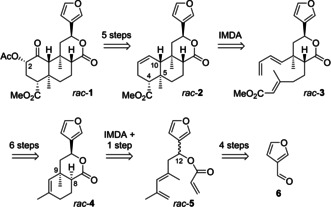
Retrosynthetic analysis of our previous synthesis^[9]^ of racemic salvinorin A (*rac*‐**1**).

In 2018, we established an 18‐step synthesis of racemic salvinorin A (*rac*‐**1**) starting from 3‐furaldehyde (**6**) that employed two intramolecular Diels‐Alder reactions (IMDA) as the key steps for construction of the tricyclic framework (Scheme 1).^[9]^ Both Diels‐Alder reactions featured good diastereocontrol and set up the relative configuration of the stereogenic centers at C‐4, C‐5, C‐10 (transformation of *rac*‐**3** to *rac*‐**2**) as well as C‐8 (with subsequent epimerization) and C‐9 (*rac*‐**5** to *rac*‐**4**). As part of the final five steps from *rac*‐**2** to *rac*‐**1**, the stereogenic center at C‐2 was installed via *cis* dihydroxylation from the β face of *rac*‐**2** and subsequent Mitsunobu inversion. In conclusion, except for C‐12, each stereogenic center was formed diastereoselectively under substrate control. Therefore, transition from our route in the racemic series to an asymmetric synthesis would be easily achieved by enantioselective construction of the stereogenic center at C‐12 (see structure **5**).

**Scheme 2:**
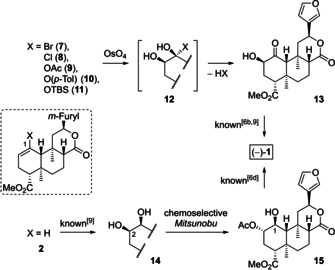
Possible pathways for streamlining of the final synthetic sequence.

Moreover, we strived for a streamlining of the final synthetic sequence (Scheme 2). While we used a selective triethylsilylether monoprotection to differentiate the two secondary alcohols formed upon dihydroxylation of *rac*‐**2**, we now wanted to realize a completely protecting‐group‐free synthesis. We envisioned to install a suitable substituent X at C‐1 to combine the dihydroxylation step with a subsequent collapse of intermediate **12** to form α‐hydroxy ketone **13** in a single step.^[10]^ The known Mitsunobu inversion of **13**[^6b^, ^9^] would complete the synthesis of (−)‐salvinorin A (**1**). An alternative strategy was inspired by the work of O'Doherty et al.^[11]^ who reported a chemoselective Mitsunobu inversion of a cyclohexane *syn* 1,2‐diol with conversion of the axial hydroxy group only. According to our NMR analysis of racemic diol **14**,^[9]^ the hydroxy group at C‐2 indeed occupies an axial position, which provides the opportunity for a selective transformation of **14** to give **15**. Oxidation of **15** to afford (−)‐**1** was already reported by Forsyth et al.^[6d]^

## Results and Discussion

**Scheme 3:**
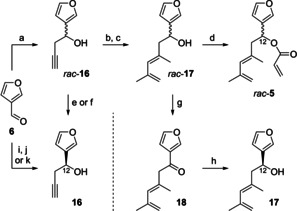
Synthesis of the racemic IMDA substrate *rac*‐**5** and various strategies for asymmetric construction of the stereogenic center at C‐12. a) Mg, cat. HgCl_2_, propargyl bromide, Et_2_O, −78 °C to 0 °C, 40 min, 97 %; b) Cp_2_ZrCl_2_, AlMe_3_, CH_2_Cl_2_, RT, overnight, then I_2_, THF, −50 °C, 2 h, 77 %; c) CuTC, (2‐propenyl)SnBu_3_, NMP, 0 °C, 24 h, 95 %; d) acryloyl chloride, cat. DMAP, NEt_3_, CH_2_Cl_2_, −78 °C to RT, 5 h, 91 %; e) (−)‐DIPT, Ti(O*i*‐Pr)_4_, 0.65 equiv. TBHP, CH_2_Cl_2_, −21 °C, 24 h, 44 %, 99 % *ee*; f) cat. (−)‐DIPT, cat. Ti(O*i*‐Pr)_4_, 0.65 equiv. TBHP, CH_2_Cl_2_, −21 °C, 48 h, 43 %, 94 % *ee*; g) DMP, NaHCO_3_, CH_2_Cl_2_, 0 °C to RT, 35 min, 96 %; h) cat. [Ru(*p*‐cymene)Cl_2_]_2_, cat. (*S*,*S*)‐TsDPEN, KOH, *i*‐PrOH, RT, 38 h, 66 %, 99 % *ee*; i) cat. **19**, **22**, PhMe, 4 Å molecular sieves, 0 °C, 6 d, 64 % **16**, 81 % *ee*; j) cat. **20**, SiCl_4_, **23**, CH_2_Cl_2_, −30 °C, 18 h, 96 % **16**, 77 % *ee*; k) cat. **21**, **23**, CH_2_Cl_2_, −35 °C to 0 °C, 19 h, then RT, 3 d, 80 % **16**, 97 % *ee*. Cp=cyclopentadienyl, TC=thiophene‐2‐carboxylate, NMP=*N*‐methyl‐2‐pyrrolidone, DMAP=4‐dimethylaminopyridine, DIPT=diisopropyl tartrate, TBHP=*tert*‐butylhydroperoxide, DMP=Dess–Martin periodinane, Ts=Tosyl, DPEN=1,2‐diphenyl‐1,2‐ethylenediamine.

We started our investigations with the asymmetric synthesis of Diels‐Alder substrate **5**, the racemic form of which is readily available in four steps starting from 3‐furaldehyde (**6**; Scheme 3).[^7d^, ^9^] For enantioselective construction of the stereogenic center at C‐12 we pursued several strategies.

Kinetic resolution of the racemic homopropargylic alcohol *rac*‐**16** ^[7d]^ using the Sharpless asymmetric epoxidation^[12]^ delivered the desired (*S*)‐enantiomer **16** in 44 % yield with 99 % *ee* (stoichiometric method) or 43 % yield with 94 % *ee* (catalytic method), respectively. However, yields are limited to 50 % using this methodology. Alternatively, alkyne *rac*‐**16** was first converted to dienol *rac*‐**17**.[^7d^, ^9^] After oxidation with DMP, the resulting dienone **18** was subjected to an asymmetric Noyori reduction^[13]^ to give the (*S*)‐configured dienol **17** in fair yield (66 %) with excellent enantioselectivity (99 % *ee*).

**Figure 1:**
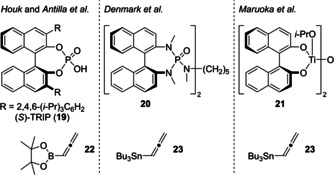
Examples of known catalytic systems for enantioselective propargylation of aldehydes – chiral catalysts (top line) and corresponding nucleophiles (bottom line).^[16]^

A third and most direct strategy replaced the Grignard propargylation of **6** by an asymmetric method (Scheme 3). In 2006, Singaram et al. achieved this transformation under indium‐mediated Barbier‐like conditions using stoichiometric amounts of (1*S*,2*R*)‐(+)‐2‐amino‐1,2‐diphenylethanol as a chiral auxiliary.^[14a]^ The groups of Evans^[6a]^ and Prisinzano^[14b]^ also converted 3‐furaldehyde (**6**) to homopropargylic alcohol **16**. However, four or five steps were needed, respectively. Our aim was to apply a methodology using a chiral catalyst.^[15]^ Specifically, we focused on catalysts bearing chiral binaphthyl moieties that already performed well for asymmetric propargylation (Figure 1). Independently of each other, Houk and Antilla et al. as well as Reddy developed a protocol using (*S*)‐TRIP (**19**) as the chiral catalyst and allenyl pinacol borane (**22**) as the propargylation agent.[^16a^, ^16b^] Adaption to 3‐furaldehyde (**6**) gave the desired homopropargylic alcohol **16** in 64 % yield with 81 % *ee* (Scheme 3). Furthermore, we applied Denmark's chiral phosphoramide **20** in combination with SiCl_4_ as a Lewis acid, which can be used for both allylation and propargylation of aldehydes.^[16c]^ Using allenyltributylstannane (**23**) as the nucleophile, **16** was obtained in high yield (96 %) with 77 % ee. Finally, we also tested Maruoka's chiral bis‐titanium(IV)‐oxide catalyst **21** with **23** as the nucleophile.^[16d]^ To our delight, **16** was isolated in 80 % yield with an excellent 97 % *ee*. Noteworthy, upscaling of this reaction up to 1.5 g 3‐furaldehyde (**6**) affected neither the yield nor the enantioselectivity. Due to the high *ee* of the desired homopropargylic alcohol **16**, the option of running the reaction on a gram scale, and the facile in situ generation of catalyst **21**, we selected Maruoka's system for our synthetic purpose.

**Scheme 4:**
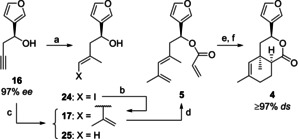
Synthesis of enantiopure lactone **4**. a) cat. Cp_2_ZrCl_2_, AlMe_3_, H_2_O, CH_2_Cl_2_, −25 °C to RT, 5 h, then I_2_, THF, −50 °C to RT, 17 h, 74 % **24**; b) CuTC, (2‐propenyl)SnBu_3_, NMP, 0 °C, 24 h, 95 %; c) performed with *rac*‐**16**: cat. Cp_2_ZrCl_2_, AlMe_3_, H_2_O, CH_2_Cl_2_, −30 °C to RT, 4 h, then cat. Pd_2_dba_3_, 2‐bromopropene, THF, reflux, 18 h, crude mixture: 54 % *rac*‐**17**+13 % *rac*‐**25**, purely isolated: 51 % *rac*‐**17**, 3 % *rac*‐**25**; d) acryloyl chloride, cat. DMAP, NEt_3_, CH_2_Cl_2_, −78 °C to RT, 16 h, 92 % **5**; e) BHT, PhCl, sealed tube, 183 °C, 2.8 d, then RT, 19 h, 94 % **4**, 91 % *ds*; f) LiHMDS, THF, −78 °C to 0 °C, 2 h, then MeOH, −95 °C to −86 °C, 1 h, 87 %, ds≥97 %. BHT=2,6‐di‐*tert*‐butyl‐4‐methylphenol.

With almost enantiopure **16** (97 % *ee*) in hand, preparation of lactone **4** was achieved as for our synthesis in the racemic series[^7d^, ^9^] through carboalumination/iodolysis of alkyne **16** to vinyl iodide **24**, Liebeskind coupling to give diene **17**, acylation of the alcohol, IMDA of the resulting acrylate **5**, and subsequent de‐/reprotonation to increase the diastereomeric purity of **4**. Some parts of this known sequence were studied in more depth (Scheme 4): (1) Stoichiometric carboalumination of alkyne **16** ^[7d]^ was replaced by Wipf's catalytic version^[17]^ without significant loss of yield. (2) Installation of the diene moiety of **17** was alternatively accomplished in a one‐pot process by Pd‐catalyzed cross‐coupling of the intermediate vinylalane with 2‐bromopropene. After intensive optimization of the reaction conditions performed with the racemic starting material *rac*‐**16**, a mixture of diene *rac*‐**17** and olefin *rac*‐**25** was obtained in 54 % and 13 % yield (NMR), respectively. Formation of olefin *rac*‐**25** could not be prevented, but after double flash chromatography the desired diene *rac*‐**17** was isolated nearly without loss in 51 % yield as a pure compound. Regarding the efficiency of this transformation, the two‐step procedure (74 %^[7d]^ for carboalumination/iodolysis+95 %^[9]^ for Liebeskind coupling=70 % over two steps) is still superior to the one‐pot reaction. (3) Use of stoichiometric amounts of the radical inhibitor BHT increased the yield of the IMDA of **5** to 94 %.

**Scheme 5:**
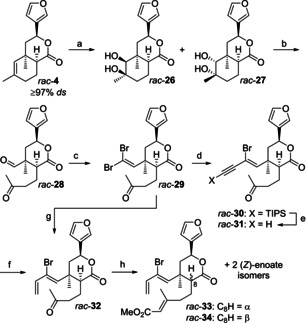
Preparation of IMDA substrates *rac*‐**33** and *rac*‐**34**. a) cat. OsO_4_, NMO, acetone, H_2_O, 0 °C to RT, 24 h, 69 %; b) PIDA, CH_2_Cl_2_, RT, 1.5 h, 88 %; c) CBr_4_, PPh_3_, CH_2_Cl_2_, 0 °C, 45 min, 95 %; d) cat. Pd(DPEphos)Cl_2_, cat. CuI, NEt_3_, TIPS‐acetylene, PhH, 3 °C to 5 °C, 35 min, 87 %; e) TBAF, HOAc, THF, −70 °C to 0 °C, 50 min, 100 %; f) cat. (IPr)Cu(O*t*‐Bu), PMHS, *i*‐BuOH, PhMe, RT, 24 h, 99 %; g) cat. Pd_2_dba_3_, cat. P(*o*‐furyl)_3_, Bu_3_Sn(vinyl), PhMe, RT, 24 h, 90 %; h) NaH, methyl diethylphosphonoacetate, THF, RT, 24 h, 41 % *rac*‐**33**, 11 % *rac*‐**34**. NMO=*N*‐methylmorpholine *N*‐oxide, PIDA=phenyliodine(III) diacetate, DPEphos=bis(2‐diphenylphosphino)phenyl ether, TBAF=tetrabutylammonium fluoride, IPr=1,3‐bis(2,6‐di‐*iso*‐propylphenyl)imidazol‐2‐ylidene, PMHS=polymethylhydrosiloxane.

For a first evaluation of the idea to use functionalized cyclohexenes **7–11** (see Scheme 2) as intermediates en route to the target diterpene **1**, lactone **4** was employed as the racemic mixture (Scheme 5). Thus, *rac*‐**4** was converted to keto aldehyde *rac*‐**28** as previously reported.^[9]^ We first focused on the preparation of the brominated tricycle *rac*‐**7**, which should be available by IMDA using a 2‐bromo‐1,3‐diene moiety.^[18]^ For substrate synthesis, keto aldehyde *rac*‐**28** was subjected to a Ramirez dibromoolefination.^[19]^ Installation of the diene moiety could then be achieved either by a three‐step protocol consisting of (*E*)‐selective Sonogashira cross‐coupling^[20]^ of *rac*‐**29** with TIPS‐acetylene, desilylation, and alkyne semi‐reduction^[21]^ or preferably by a direct (*E*)‐selective Stille cross‐coupling^[22]^ of dibromoolefin *rac*‐**29**. The resulting ketone *rac*‐**32** was subjected to Horner‐Wadsworth‐Emmons (HWE) olefination. In analogy to our previous work,^[9]^ partial C‐8 epimerization occurred, leading to four diastereomers *rac*‐**33**, *rac*‐**34**, and two (*Z*)‐enoate isomers. Enoate *rac*‐**33**, which was isolated as a pure compound by simple flash chromatography, could in principle be a suitable IMDA substrate given the option of post‐cycloaddition epimerization at C‐8. On the other hand, *rac*‐**34** could only be separated from *rac*‐**33** by semi‐preparative HPLC. Moreover, compounds *rac*‐**33** and *rac*‐**34** were rather unstable and should be used for the next reaction step quickly. Due to this lability and the laborious isolation of pure *rac*‐**34**, we did not favor C‐8 epimerization of *rac*‐**33** at this stage.

**Scheme 6:**
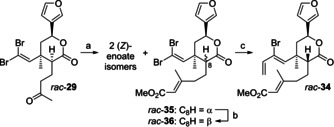
Alternative preparation of IMDA substrate *rac*‐**34**. a) NaH, methyl diethylphosphonoacetate, THF, RT, 25 h, 32 % *rac*‐**35**, 15 % *rac*‐**36**; b) DBU, CH_2_Cl_2_, 0 °C to RT, 2 h, 22 % (81 % brsm); c) cat. Pd_2_dba_3_, cat. P(*o*‐furyl)_3_, Bu_3_Sn(vinyl), PhMe, RT, 26 h, 76 %. DBU=1,8‐diazabicyclo[5.4.0]undec‐7‐ene.

In an alternative attempt for the preparation of IMDA substrate *rac*‐**34**, we simply changed the order of events and built the dienophile moiety first through HWE reaction of ketone *rac*‐**29** still bearing the dibromoolefin substructure (Scheme 6).

Again, C‐8 epimerization occurred during HWE olefination, but all four isomers could be separated from each other by simple flash chromatography. Unfortunately, additional formation of products with a bromo alkyne moiety was observed that contaminated the product fractions of *rac*‐**36** and *rac*‐**35**. The latter compound was subjected to C‐8 epimerization with DBU delivering *rac*‐**36** in 22 % (81 % brsm) yield along with reisolated starting material. After running this reaction a few times, the majority of *rac*‐**35** was transformed to *rac*‐**36**, which was then submitted to Stille coupling. To our delight, double flash chromatography afforded *rac*‐**34** as a pure compound free from bromoalkyne or eneyne impurities.

**Scheme 7:**
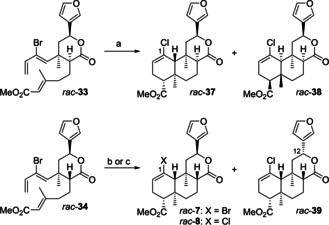
IMDA of substrates *rac*‐**33** and *rac*‐**34**. a) BHT, PhCl, sealed tube, 200 °C, 4.5 d, 85 %, *dr*=76 : 24; b) BHT, PhCl, sealed tube, 200 °C, 4.6 d, 72 % *rac*‐**7** (84 % brsm), 98 % *ds*; c) BHT, PhCl, sealed tube, 200 °C, 3.7 d, 44 % *rac*‐**8**, 90 % purity, 14 % *rac*‐**39**, >99 % *ds*.

Both trienes *rac*‐**33** and *rac*‐**34** were subjected to the crucial second IMDA reaction (Scheme 7). For *rac*‐**33**, featuring a *cis* relationship between diene and dienophile, several runs of the IMDA were accompanied by either partial or complete Br/Cl‐exchange at C‐1. Since we used the radical inhibitor BHT in large excess, it is unlikely that this exchange occurs via a radical mechanism with the solvent PhCl acting as the chlorine source. Alternatively, traces of transition metals could be responsible for this unexpected process.^[23]^ To the best of our knowledge, such a halogen exchange during Diels‐Alder reactions has not been reported yet. Thus, one run gave a mixture of cycloadducts *rac*‐**37** and *rac*‐**38**, presumably formed via two different *endo*‐chair transition states,^[9]^ in a combined yield of 85 % with a diastereomeric ratio of 3 : 1.

In anticipation of an improved diastereoselectivity,^[9]^ we examined the IMDA of *rac*‐**34** with a *trans* relationship between diene and dienophile. Unfortunately, this reaction lacked reproducibility over the six runs made. Scheme 7 illustrates this issue exemplified by the results of two different runs under similar reaction conditions. Whereas in one case Br/Cl‐exchange only occurred in negligible amounts and provided the desired cycloadduct *rac*‐**7** in good yield (72 %, 84 % brsm) with excellent diastereoselectivity (98 %), complete Br/Cl‐exchange took place to deliver *rac*‐**8** contaminated by 10 % of an unknown isomer and *rac*‐**39** as the only products in another experiment. From a mechanistic point of view, formation of *rac*‐**7**/*rac*‐**8** is assumed via an *endo*‐chair transition state,^[9]^ whereas *rac*‐**39** is formed by epimerization at C‐12.^[24]^ The use of bromobenzene as the solvent to prevent formation of chlorine‐containing cycloadducts only led to decomposition of the substrate *rac*‐**34**.

**Scheme 8:**
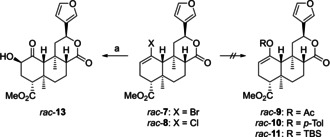
Attempts for utilization of IMDA products *rac*‐**7** and *rac*‐**8**. a) OsO_4_, H_2_O_2_, NaHCO_3_, THF/PhMe/H_2_O, for *rac*‐**7**: 0 °C to RT, 20 h, 5 % (29 % brsm), for *rac*‐**8**: 0 °C to 60 °C, 72 h, 20 % (38 % brsm).

Nevertheless, we had obtained enough material of *rac*‐**7** and *rac*‐**8** to investigate our initial strategy for the direct synthesis of *rac*‐**13** (Scheme 8). We tested a wide range of conditions for dihydroxylation, such as (1) stoichiometric use of OsO_4_, (2) OsO_4_/H_2_O_2_ according to Vogel et al.,^[10c]^ (3) various reagents for reoxidation of Os(VI) such as trimethylamine *N*‐oxide^[25]^ and potassium perchlorate,^[26]^ (4) additives like citric acid^[27]^ or 3,5‐lutidine for ligand acceleration, (5) Narasaka's modification,^[28]^ (6) Sharpless asymmetric dihydroxylation,^[29]^ and (7) Ru‐catalysis.^[30]^ In general, vinyl chloride *rac*‐**8** exhibited higher reactivity than vinyl bromide *rac*‐**7**, but in all cases yields remained in a very poor range. The best results were achieved applying Vogel's conditions^[10c]^ to furnish *rac*‐**13** in 5 % yield (from vinyl bromide *rac*‐**7**) or 20 % yield (from vinyl chloride *rac*‐**8**), respectively. Furthermore, synthesis of *rac*‐**13** through epoxidation of *rac*‐**7** or *rac*‐**8** with *m*‐CPBA^[29]^ or methyltrioxorhenium^[31]^ failed as well. We tried to overcome this issue by converting vinyl halides *rac*‐**7** or *rac*‐**8** into the vinyl acetate^[32]^ *rac*‐**9**, or aryl^[33]^ and silyl ethers^[34]^ *rac*‐**10** or *rac*‐**11**. Unfortunately, no conversion to any of the desired compounds could be achieved.

**Scheme 9:**
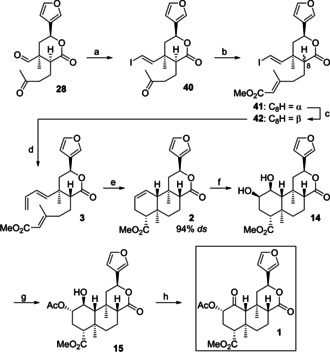
Final synthetic steps to (−)‐salvinorin A (**1**). a) CrCl_2_, CHI_3_, THF, 8 °C to 10 °C, 5 h, 58 %; b) NaH, methyl diethylphosphonoacetate, DME, RT, 48 h, 40 % **41**, 21 % **42**; c) DBU, CH_2_Cl_2_, RT, 3 h, 34 % (100 % brsm); d) cat. Pd(MeCN)_2_Cl_2_, Bu_3_Sn(vinyl), NMP, 24 h, 0 °C, 93 %; e) BHT, PhCl, sealed tube, 200 °C, 87.5 h, 66 % (88 % brsm), 94 % *ds*; f) OsO_4_, 3,5‐lutidine, THF, toluene, 0 °C to RT, 24 h, 95 %; g) Ph_3_P, DBAD, HOAc, THF, 60 °C, 47 h, 50 % (61 % brsm); h) cat. TPAP, NMO, CH_2_Cl_2_, 4 Å molecular sieves, RT, 2 h, 92 %. DME=dimethoxyethane, DBAD=di‐*tert*‐butyl azodicarboxylate, TPAP=tetrapropylammonium perruthenate.

As a consequence, the IMDA strategy using a 2‐bromo‐1,3‐diene moiety was abandoned, and no further efforts were made to optimize the production of *rac*‐**7** or *rac*‐**8** by cycloaddition. Instead, we then focused on shortening our access to (−)‐salvinorin A (**1**) by a chemoselective Mitsunobu inversion of diol **14** (Scheme 9). Thus, keto aldehyde **28** prepared in two steps from lactone **4** (97 % *ee*) was converted to diol **14** in six steps according to the route developed in the racemic series.^[9]^ After optimization of reaction time, temperature, and amounts of reagents, we were indeed able to convert **14** to monoacetate **15** in 50 % (61 % brsm) yield along with reisolated starting material. Although a large excess of reagents was required, isolation of the pure product **15** was achieved without problems by simple flash chromatography. Finally, oxidation^[6d]^ of alcohol **15** furnished (−)‐salvinorin A (**1**), the spectroscopic data of which were in full agreement with the literature.^[1]^

## Conclusion

We succeeded in the transition from our previous total synthesis of racemic salvinorin A (*rac*‐**1**) into a streamlined enantioselective version. Several systems for catalytic asymmetric propargylation of aldehydes were applied to 3‐furaldehyde (**6**), and the methodology of Maruoka et al. was found to be superior, especially due to its high enantioselectivity. With virtually enantiopure homopropargyl alcohol **16** in hand, our synthesis in the racemic series was reproduced with some improvements up to the stage of diol **14**. Finally, the route to (−)‐salvinorin A (**1**) was shortened by two steps through application of a chemoselective Mitsunobu esterification. Overall, (−)‐salvinorin A (**1**) was synthesized from the commercially available 3‐furaldehyde (**6**) in only 16 steps with 1.4 % total yield without the use of any protecting group. Thus, this second‐generation synthesis represents the shortest enantioselective route to **1** with the highest overall yield.

## Experimental Section

Experimental Details see Supporting Information.

## Conflict of interest

The authors declare no conflict of interest.

## Supporting information

As a service to our authors and readers, this journal provides supporting information supplied by the authors. Such materials are peer reviewed and may be re‐organized for online delivery, but are not copy‐edited or typeset. Technical support issues arising from supporting information (other than missing files) should be addressed to the authors.

Supplementary
