# Supplementary material for: A Protecting‐Group‐Free Synthesis of (−)‐Salvinorin A
Source: Chemistry. 2021 May 2;27(29):7968–73. doi: 10.1002/chem.202100560 (PMC8252117; doi:10.1002/chem.202100560)
Supplement: Supplementary file 1 — Supplementary [file CHEM-27-7968-s001.pdf]

Chemistry–A European Journal

Supporting Information

A Protecting-Group-Free Synthesis of (−)-Salvinorin A

Patrick Zimdars, Yuzhou Wang, and Peter Metz*

Table of Contents

1 General information 3
2 Experimental procedures 3
2.1 Kinetic resolution of homopropargylic alcohol *rac*-16 3
2.2 DMP oxidation of dienol *rac*-17 to give dienone 18 5
2.3 Asymmetric Noyori reduction of dienone 18 to give 17 5
2.4 Asymmetric propargylation of 3-furaldehyde (6) to give 16 6
2.5 Carboalumination and subsequent iodolysis of 16 to give 24 9
2.6 Liebeskind coupling of 24 to give 17 10
2.7 Carboalumination and subsequent cross-coupling of *rac*-16 to give *rac*-17 10
2.8 *O*-Acylation of 17 to give 5 and subsequent IMDA to give 4 11
2.9 Dibromoolefination of keto aldehyde *rac*-28 to give *rac*-29 11
2.10 (*E*)-selective Sonogashira coupling of *rac*-29 to give *rac*-30 12
2.11 Desilylation of TIPS-alkyne *rac*-30 to give *rac*-31 12
2.12 Semi-reduction of alkyne *rac*-31 to give *rac*-32 13
2.13 (*E*)-selective Stille coupling of dibromoolefin *rac*-29 to give *rac*-32 13
2.14 HWE reaction of ketone *rac*-32 to give *rac*-33 and *rac*-34 14
2.15 HWE reaction of ketone *rac*-29 to give *rac*-35 and *rac*-36 15
2.16 Epimerization of lactone *rac*-35 to give *rac*-36 16
2.17 Epimerization of bromo alkyne *rac*-SI-11 to give *rac*-SI-10 17
2.18 (*E*)-selective Stille coupling of dibromoolefin *rac*-36 to give *rac*-34 18
2.19 IMDA of triene *rac*-33 to give *rac*-37 18
2.20 IMDA of triene *rac*-34 to give *rac*-7 and *rac*-8 19
2.21 Dihydroxylation of vinyl bromide *rac*-7 to give *rac*-13 20
2.22 Dihydroxylation of vinyl chloride *rac*-8 to give *rac*-13 21
2.23 Conversion of lactone 4 to diol 14 21
2.24 Chemoselective Mitsunobu inversion of diol 14 to give 15 22
2.25 Oxidation of alcohol 15 to give (–)-salvinorin A (1) 22
3 References 23
4 NMR spectra 24
4.1 Compound 18 24
4.2 Compound 17 24
4.3 Compound *rac*-25 25
4.4 Compound 5 25
4.5 Compound *rac*-29 26
4.6 Compound *rac*-30 26
4.7 Compound *rac*-31 27
4.8 Compound *rac*-32 28
4.9 Compound *rac*-SI-6 28
4.10 Compound *rac*-SI-7 29
4.11 Compound *rac*-33 30
4.12 Compound *rac*-34 31
4.13 Compound *rac*-SI-8 31
4.14 Compound *rac*-SI-9 32
4.15 Compound *rac*-36 33
4.16 Compound *rac*-35 33
4.17 Compound *rac*-SI-11 34
4.18 Compound *rac*-SI-10 35

4.19 Compound *rac*-37 35
4.20 Compound *rac*-38 36
4.21 Compound *rac*-7 37
4.22 Compound *rac*-8 37
4.23 Compound *rac*-39 38
4.24 Compound 15 39
4.25 Compound 1 39

1 General information

THF, toluene, and CH_2Cl_2 were dried and purified by passage through a MB-SPS-800 device using molecular sieves. Et_3N was freshly distilled over CaH_2, and chlorobenzene was dried over molecular sieves 4Å. Allenyltributylstannane (**23**),[1] carbene complex (IPr)Cu(O*t*-Bu),[2,3] Denmark's catalyst **20**,[4] and tributylvinylstannane (**SI-1**)[5] were prepared according to known procedures. All other commercially available reagents were used as received. Reactions were performed under argon atmosphere. Sealed glass tubes were used for intramolecular Diels-Alder reactions. Thin layer chromatography (TLC) was performed on Merck silica gel 60 F_{254} 0.2 mm precoated plates. Product spots were visualized by UV light at 254 nm and subsequently developed using anisaldehyde or vanillin solution as appropriate. Flash column chromatography was carried out using silica gel (Merck, particle size 40–63 microns). Semi-preparative HPLC separations were performed with a Waters 600 pump, a Waters 600 controller, a Waters 2996 photodiode array detector, and a Macherey-Nagel SP 250/21 Nucleosil 100-7 column. Melting points were measured on a Wagner & Munz PolyTherm A and are uncorrected. Infrared spectra were recorded on a THERMONICOLET Avatar 360 instrument using ATR. NMR spectra were recorded on a Bruker AC 300 P (300 MHz ^{1}H, 75 MHz ^{13}C), on a Bruker DRX 500 P (500 MHz ^{1}H, 126 MHz ^{13}C), or on a Bruker AC 600-P (600 MHz ^{1}H, 151 MHz ^{13}C) spectrometer. Chemical shifts (*δ*) are quoted in parts per million (ppm) downfield of tetramethylsilane, using residual proton-containing solvent as internal standard (^{1}H: $CDCl_3$ at 7.26 ppm, ^{13}C: $CDCl_3$ at 77.16 ppm). Abbreviations used in the description of resonances are: s (singlet), d (doublet), t (triplet), q (quartet), br (broad). Coupling constants (*J*) are quoted to the nearest 0.1 Hz. Mass spectra were recorded with an Agilent 5973N detector coupled with an Agilent 6890N GC (GC-MS, 70 eV) or else with a Bruker Esquire-LC (direct injection as a methanolic NH_4OAc solution, ESI). HRMS spectra were recorded on a Bruker Daltonic "Impact II" (ESI-TOF) or on an Agilent 6538 Q-TOF. Elemental analysis was performed on a Hekatech EA 3000. Enantiomeric excesses were determined by either chiral HPLC on a Hewlett Packard LC 1090 with photo-diode array detector (DAD) using chiral column Chiralpak IA (DAICEL, 250 mm, inner diameter 4.6 mm, particle size 5 microns) or chiral GC on a Shimadzu GC-2014 with flame ionization detector using the chiral phase Hydrodex-β-6TBDM (MACHEREY-NAGEL, length 25 m, inner diameter 0.25 mm, column temperature 140 °C, air/N_2 carrier gas).

2 Experimental procedures

2.1 Kinetic resolution of homopropargylic alcohol *rac*-16

Stoichiometric version:

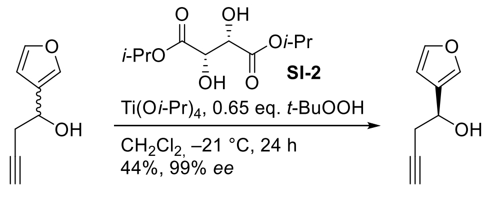

rac-**16** **16**

A mixture of titanium(IV) isopropoxide (569 mg, 2.00 mmol), molecular sieves 4Å (500 mg) and CH_2Cl_2 (10 mL) was treated at –20 °C with (–)-di-*iso*-propyl D-tartrate (**SI-2**; 576 mg, 2.46 mmol) and stirred for 15 min. After cooling to –24 °C, a solution of homopropargylic alcohol *rac*-**16**[6] (272 mg, 2.00 mmol) in CH_2Cl_2 (2 mL) was added, and stirring was continued for further 30 min. *tert*-Butyl hydroperoxide (236 µL, 5.5M in decane, 1.30 mmol) was added, and the mixture was stirred for 24 h at –21 °C. Subsequently, Me_2S (180 µL) was added, the mixture was warmed to RT and stirred for 15 min. Then, 10% aqueous tartaric acid (440 µL), Et_2O (10 mL) and NaF (2.6 g) were added. The mixture was filtered over Celite® (elution with Et_2O), and the filtrate was concentrated under reduced pressure. Purification of the residue by flash chromatography

(pentane/Et_2O 7:3) afforded homopropargylic alcohol **16** (120 mg, 0.881 mmol, 44%, 99% *ee*, analytical data see chapter 2.4).

Chiral HPLC, hexane/isopropanol 95:5, flow rate 1.0 mL/min:

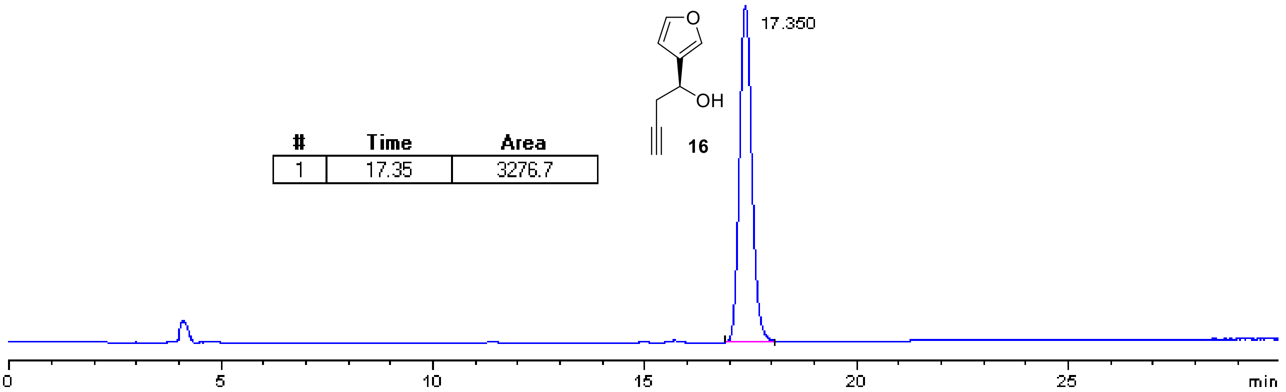

#	Time	Area
1	17.35	3276.7

<u>Catalytic version:</u>

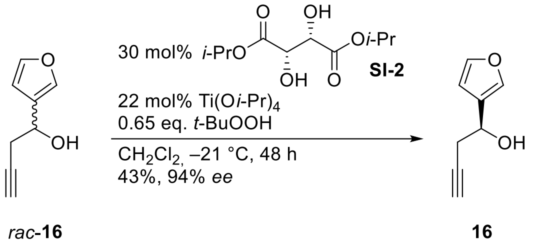

A mixture of titanium(IV) isopropoxide (154 mg, 0.542 mmol), molecular sieves 4Å (620 mg) and CH_2Cl_2 (12 mL) was treated at –20 °C with (–)-di-*iso*-propyl D-tartrate (**SI-2**; 174 mg, 0.743 mmol) and stirred for 30 min. After cooling to –28 °C, a solution of homopropargylic alcohol *rac*-**16**[6] (338 mg, 2.48 mmol) in CH_2Cl_2 (3 mL) was added, and stirring was continued for further 30 min at –23 °C. *tert*-Butyl hydroperoxide (293 µL, 5.5M in decane, 1.61 mmol) was added, and the mixture was stirred for 48 h at –21 °C. Subsequently, Me_2S (225 µL) was added, the mixture was warmed to RT and stirred for 30 min. Then, 10% aqueous tartaric acid (546 µL), Et_2O (12 mL) and NaF (3.3 g) were added. The mixture was filtered over Celite® (elution with Et_2O), and the filtrate was concentrated under reduced pressure. Purification of the residue by flash chromatography (pentane/Et_2O 7:3) afforded homopropargylic alcohol **16** (145 mg, 1.07 mmol, 43%, 94% *ee*, analytical data see chapter 2.4).

Chiral HPLC, hexane/isopropanol 95:5, flow rate 1.0 mL/min:

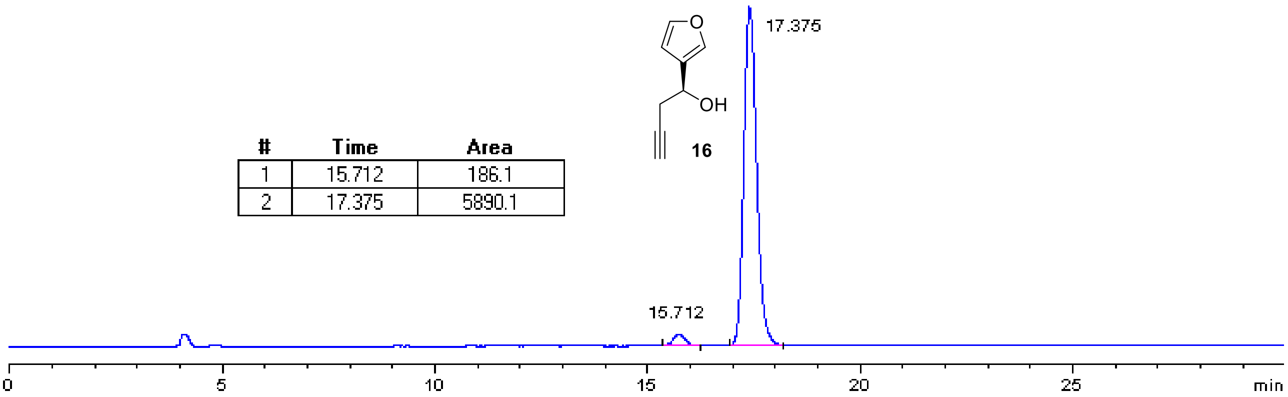

#	Time	Area
1	15.712	186.1
2	17.375	5890.1

2.2 DMP oxidation of dienol *rac*-17 to give dienone 18

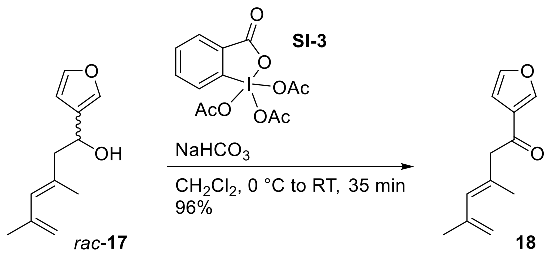

A solution of dienol *rac*-**17**[6,7] (231 mg, 1.20 mmol) in CH_2Cl_2 (12 mL) was treated at 0 °C with $NaHCO_3$ (363 mg, 4.32 mmol) and Dess–Martin periodinane (**SI-3**; 612 mg, 1.44 mmol) and stirred for 5 min at 0 °C and 30 min at RT. After addition of saturated aqueous $Na_2S_2O_3$ (4 mL) and saturated aqueous $NaHCO_3$ (4 mL), the layers were separated. The aqueous layer was extracted three times with Et_2O. The organic layers were combined, dried over $MgSO_4$, and concentrated under vacuum. The residue was purified by flash chromatography (pentane/Et_2O 6:1) to afford dienone **18** (218 mg, 1.15 mmol, 96%) as a colorless liquid.

18: R_f = 0.63 (isohexane/EtOAc 3:1); ^{1}H-NMR (300 MHz, $CDCl_3$): δ = 8.08 (dd, J = 1.5, 0.8 Hz, 1 H), 7.43 (dd, J = 1.9, 1.5 Hz, 1 H), 6.78 (dd, J = 1.9, 0.9 Hz, 1 H), 5.87–5.77 (m, 1 H), 5.03–4.98 (m, 1 H), 4.84–4.79 (m, 1 H), 3.46 (d, J = 1.1 Hz, 2 H), 1.86 (s, 3 H), 1.86 (s, 3 H); ^{13}C-NMR (75 MHz, $CDCl_3$): δ = 193.22 (s), 147.58 (d), 144.17 (d), 141.59 (s), 131.61 (d), 131.41 (s), 127.64 (s), 115.41 (t), 109.01 (d), 52.71 (t), 23.57 (q), 18.28 (q); IR (ATR) ν_{max} = 3134, 3081, 2965, 2939, 2916, 1674, 1593, 1565, 1510, 1445, 1313, 1273, 1153, 1053, 871, 814, 742 637; ESI-MS (+25 V): m/z = 191.3 $[M+H]^+$; elemental analysis calcd (%) for $C_{12}H_{14}O_2$: C 75.76, H 7.42; found: C 76.11, H 7.66.

2.3 Asymmetric Noyori reduction of dienone 18 to give 17

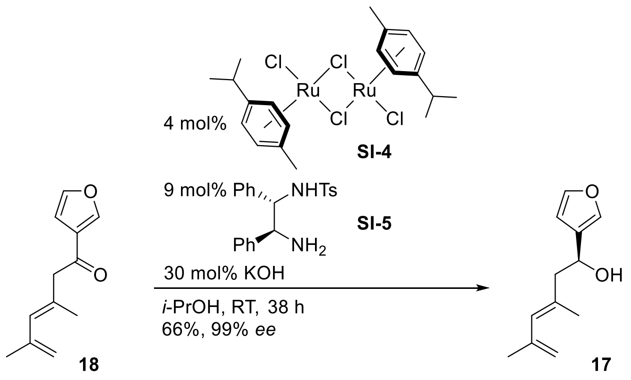

A solution of dichloro-*p*-cymol ruthenium(II) dimer (**SI-4**; 30.0 mg, 49.0 µmol) and (1*S*,2*S*)-(+)-*N*-(4-toluenesulfonyl)-1,2-diphenylethylenediamine (**SI-5**; 35.9 mg, 97.8 µmol) in CH_2Cl_2 (1.5 mL) was treated with freshly ground KOH (19.5 mg, 348 µmol) and H_2O (1 mL) and stirred for 5 min at RT. The layers were separated, and the organic layer was washed with water twice. Subsequently, the organic layer was dried carefully with CaH_2, filtered, concentrated under reduced pressure, and dried under vacuum. The solid was dissolved in isopropanol (15 mL) and added to a solution of dienone **18** (218 mg, 1.15 mmol) in isopropanol (10 mL). After stirring for 38 h at RT, the reaction mixture was concentrated under reduced pressure, filtered over a short pad of silica (elution with pentane/Et_2O 1:1), and purified by flash chromatography (pentane/Et_2O 4:1) to afford dienol **17** (146 mg, 0.761 mmol, 66%, 99% *ee*, analytical data see chapter 2.6).

Chiral GC:

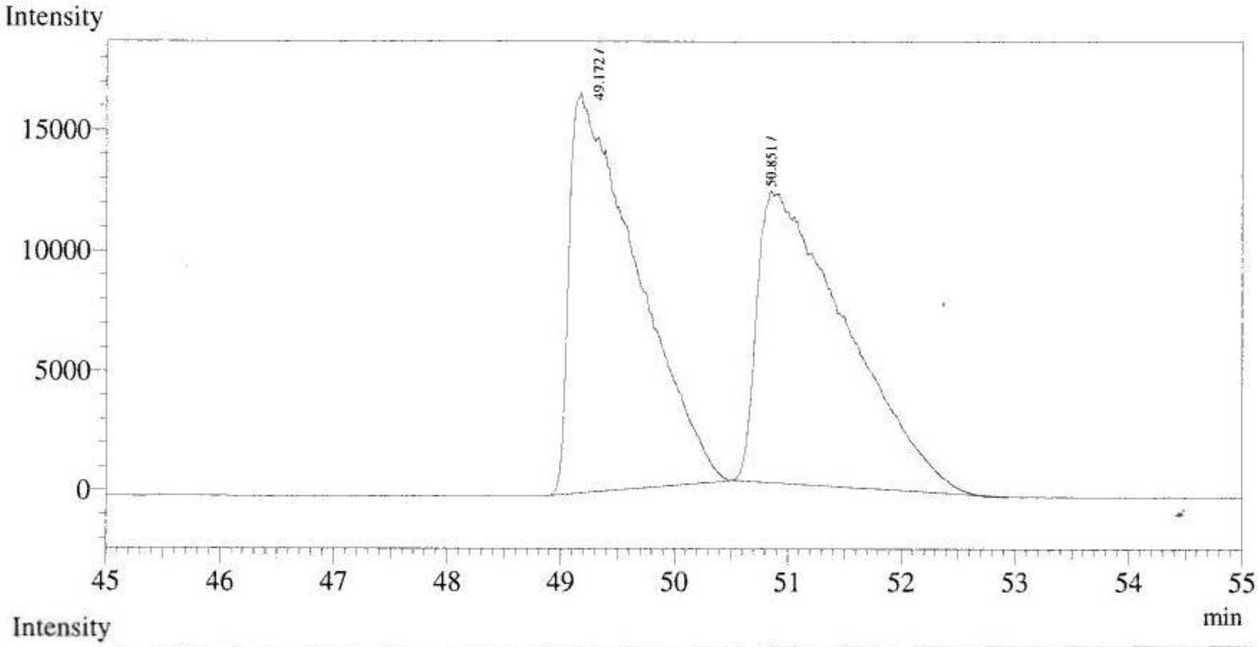

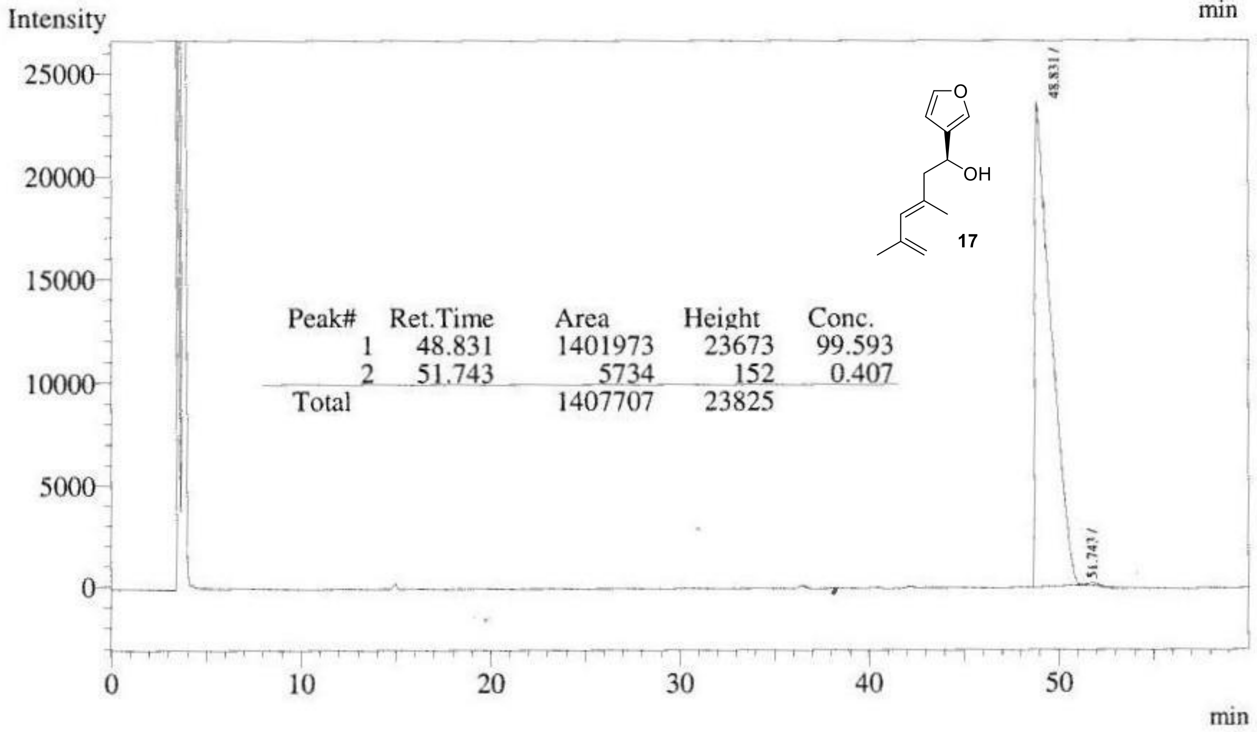

Peak#	Ret.Time	Area	Height	Conc.
1	48.831	1401973	23673	99.593
2	51.743	5734	152	0.407
Total		1407707	23825	

2.4 Asymmetric propargylation of 3-furaldehyde (6) to give 16

Methodology of Houk/Antilla et al. and Reddy:

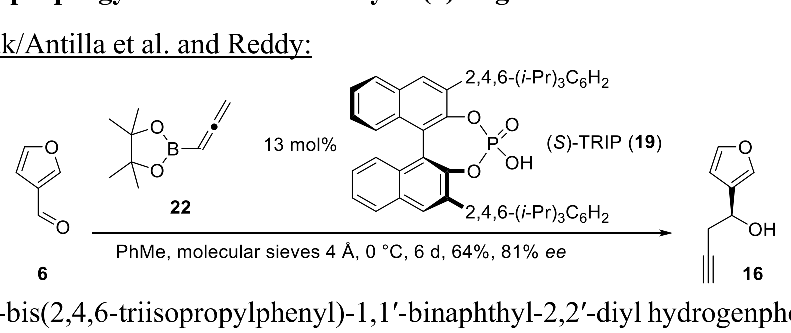

A solution of (*S*)-3,3′-bis(2,4,6-triisopropylphenyl)-1,1′-binaphthyl-2,2′-diyl hydrogenphosphate (**19**; 20.0 mg, 26.6 µmol) and 3-furaldehyde (**6**; 19.8 mg, 206 µmol) in toluene (1.5 mL) was treated with molecular sieves 4 Å (100 mg). Subsequently, allenylboronic acid pinacol ester (**22**; 62.5 mg, 365 µmol) was added dropwise at 0 °C, and stirring was continued for 6 d. The mixture was filtered, and the filtrate was concentrated under

vacuum. Flash chromatography (isohexane/EtOAc 8:1) of the residue afforded homopropargylic alcohol **16** (17.8 mg, 131 µmol, 64%, 81% *ee*, analytical data see below).

Chiral HPLC, hexane/isopropanol 90:10, flow rate 0.6 mL/min:

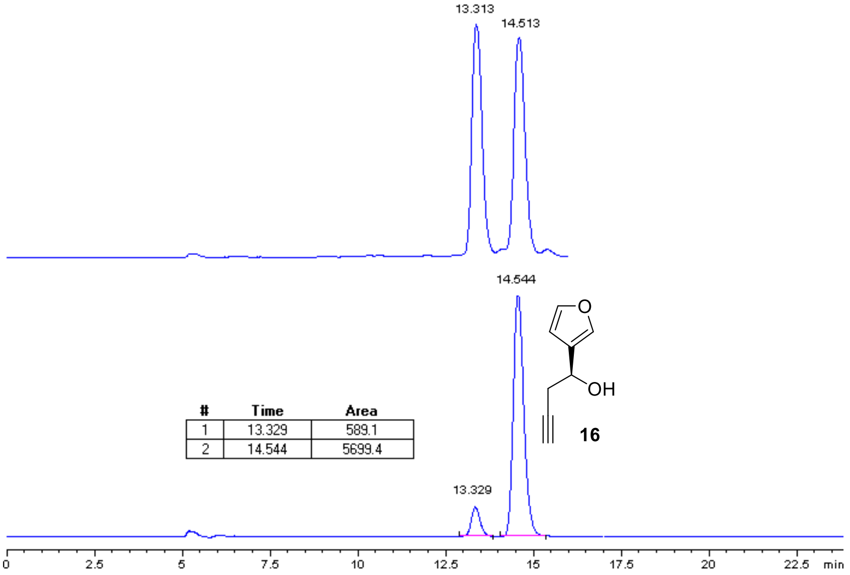

<u>Methodology of Denmark et al.:</u>

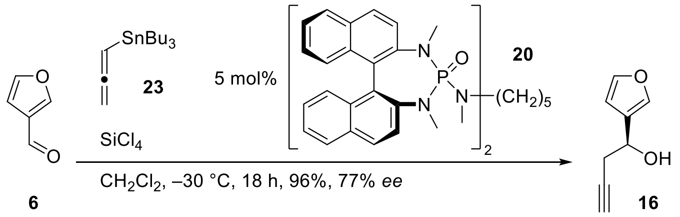

To a solution of Denmark's catalyst **20** (20.9 mg, 24.8 µmol) and allenyltributylstannane (**23**; 375 mg, 1.14 mmol) in CH_2Cl_2 (0.8 mL) were added silicon(IV) chloride (94.6 mg, 0.557 mmol) and 3-furaldehyde (**6**; 49.7 mg, 0.517 mmol) at –30 °C. After stirring for 18 h at this temperature, the mixture was poured into a vigorously stirred mixture of 1 M aqueous KH_2PO_4 (5 mL) and saturated aqueous KF (5 mL) and stirred for 45 min. After dilution with CH_2Cl_2, the aqueous layer was extracted three times with CH_2Cl_2. The organic layers were combined, dried over $MgSO_4$, and concentrated under vacuum. Flash chromatography (isohexane/EtOAc 4:1) afforded homopropargylic alcohol **16** (67.7 mg, 0.498 mmol, 96%, 77% *ee*, analytical data see below).

Chiral HPLC, hexane/isopropanol 90:10, flow rate 0.6 mL/min:

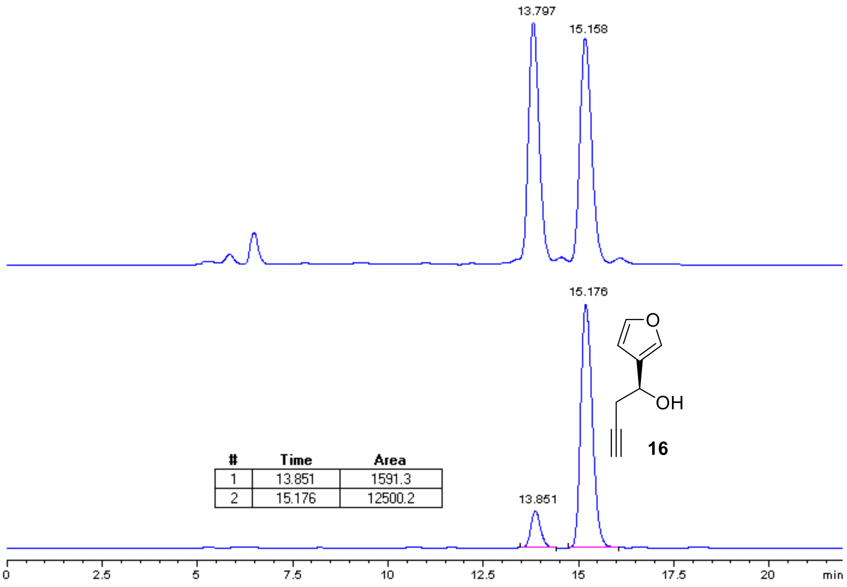

Methodology of Maruoka et al.:

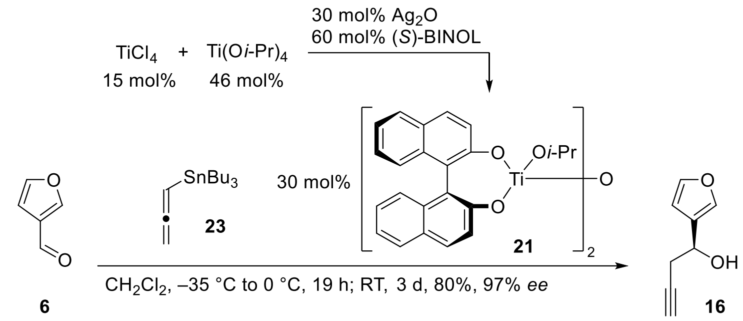

In situ preparation of Maruoka's catalyst **21** according to literature procedure:[8] To a solution of titanium(IV) chloride (1.16 g, 14% in CH_2Cl_2, 0.855 mmol) in CH_2Cl_2 (22 mL) was added titanium(IV) isopropoxide (746 mg, 2.62 mmol) at 0 °C. The mixture was stirred for 10 min at 0 °C and further 90 min at room temperature. After addition of silver(I) oxide (400 mg, 1.73 mmol), the solution was stirred for 4 h with exclusion of light. Subsequently, (*S*)-1,1′-bi-2-naphthol (985 mg, 3.44 mmol) and CH_2Cl_2 (15 mL) were added, and stirring was continued for 2 h.

To this solution were added CH_2Cl_2 (17 mL), 3-furaldehyde (**6**; 550 mg, 5.72 mmol), and allenyltributylstannane (**23**; 5.67 g, 17.2 mmol). After stirring at –35 °C for 10 min, the solution was stirred at 0 °C for 19 h and subsequently at room temperature for 3 d. After addition of saturated aqueous $NaHCO_3$, the layers were separated. The aqueous layer was extracted three times with CH_2Cl_2, the combined organic layers were washed with brine, dried over Na_2SO_4, and concentrated under vacuum. The residue was adsorbed on silica gel and purified by flash chromatography (pentane/Et_2O 2:1 + 5% NEt_3 to 1:1 + 5% NEt_3) to give homopropargylic alcohol **16** (626 mg, 4.60 mmol, 80%, 97% *ee* determined at the stage of diene **17**) as a pale-yellow liquid.

16: $[\alpha]_D^{22}$ = –27.1 (c 0.91 in $CHCl_3$); IR (ATR): ν_{max} = 3375, 3292, 2914, 1502, 1157, 1026, 874, 796, 634 cm^{-1}; ESI-MS (+10 V) m/z = 273.1 $[2M+H]^+$; elemental analysis calcd (%) for $C_8H_8O_2$: C 70.57, H 5.92; found: C 70.47, H 6.17; remaining analytical data see ref. [6].

Alkyne **16** was converted to diene **17** according to chapters 2.5 and 2.6. Then, the enantiomeric excess was determined by chiral GC:

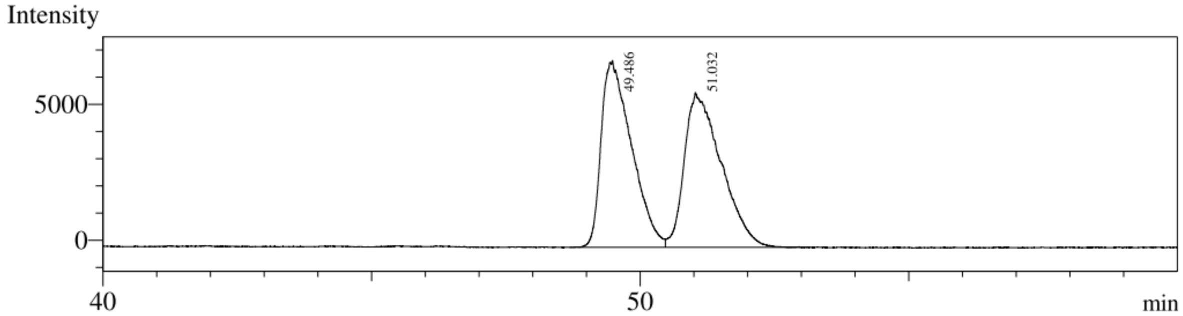

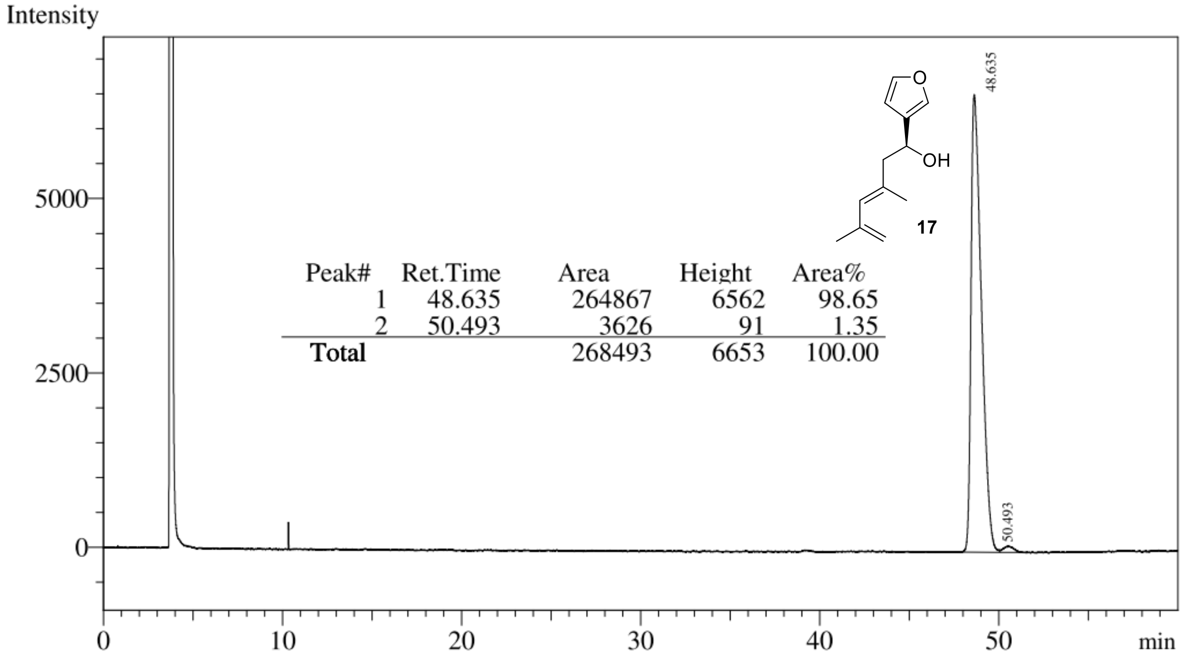

2.5 Carboalumination and subsequent iodolysis of 16 to give 24

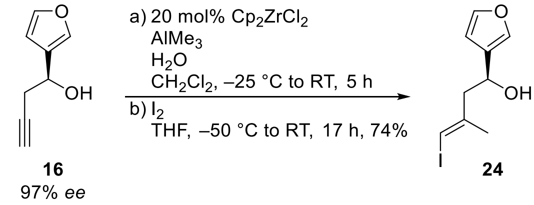

A solution of bis(cyclopentadienyl)zirconium(IV) dichloride (2.70 g, 9.25 mmol) in CH_2Cl_2 (100 mL) was treated dropwise with trimethylaluminum (68 mL, 2 M in toluene, 136 mmol) at –25 °C over a period of 20 min. After stirring for further 15 min, water (0.82 mL, 46 mmol) was added carefully over 10 min, and stirring was continued for 15 min. Subsequently, a solution (prepared at –25 °C) of alkyne **16** (6.21 g, 45.6 mmol, 97% *ee*) and trimethylaluminum (27 mL, 2 M in toluene, 54 mmol) in CH_2Cl_2 (48 mL) was added dropwise over 30 min. Subsequently, the solution was stirred at –25 °C for further 15 min, warmed to room temperature, and stirred for 5 h. After cooling to –50 °C, iodine (18.5 g, 73.0 mmol) in THF (50 mL) was added over 30 min. Stirring was continued for 17 h, while the solution was allowed to warm to room temperature slowly. After addition of a 1:1 mixture of saturated aqueous $Na_2S_2O_3$ and saturated aqueous $NaHCO_3$, the suspension was filtered (elution with Et_2O). The filtrate was diluted with water, and the aqueous layer was extracted three times with Et_2O. The organic layers were combined, washed with brine, dried over $MgSO_4$, and concentrated under vacuum. Purification by flash chromatography (isohexane/EtOAc 4:1) afforded vinyl iodide **24** (9.44 g, 33.9 mmol, 74%) as a pale-yellow liquid.

24: $[\alpha]_D^{24}$ = –35.2 (*c* 4.35 in CH_2Cl_2); remaining analytical data see ref. [6].

2.6 Liebeskind coupling of 24 to give 17

Liebeskind coupling of **24** to give **17** was conducted according to the literature procedure.[7]

17: colorless solid; m.p. 42–44 °C; $[\alpha]_D^{24}$ = –58.8 (*c* 1.26 in CH_2Cl_2); ^{13}C NMR (75 MHz, $CDCl_3$): δ = 143.36 (d), 141.70 (s), 139.09 (d), 133.51 (s), 131.09 (d), 128.70 (s), 115.13 (t), 108.68 (d), 64.66 (d), 49.84 (t), 23.79 (q), 18.06 (q); IR (ATR): ν_{max} = 3392, 3354, 2935, 1640, 1494, 1464, 1266, 1153, 1043, 1019, 956, 889, 871, 852, 788, 763, 740, 673 cm^{-1}; MS (70 eV): *m/z* (%) = 192 (3) $[M]^{\bullet+}$, 174 (7), 97 (100) [(Furyl)C^+HOH], 96 (56) [(Furyl)$C^+HO^\bullet$], 95 (13) $[C_7H_{11}]^+$, 81 (42) $[C_6H_9]^+$, 69 (27), 41 (27) $[C_3H_5]^+$; elemental analysis calcd (%) for $C_{12}H_{16}O_2$: C 74.97, H 8.39; found: C 74.89, H 8.47; remaining analytical data see ref. [6].

2.7 Carboalumination and subsequent cross-coupling of *rac*-16 to give *rac*-17

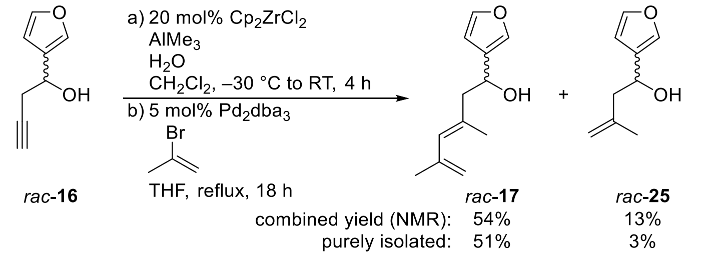

A solution of bis(cyclopentadienyl)zirconium(IV) dichloride (417 mg, 1.43 mmol) in CH_2Cl_2 (17 mL) was treated dropwise with trimethylaluminum (10.7 mL, 2 M in toluene, 21.4 mmol) at –30 °C over a period of 10 min. After stirring for further 20 min, water (0.13 mL, 7.2 mmol) was added carefully over 5 min, and stirring was continued for 20 min. Subsequently, a solution (prepared at –30 °C) of racemic alkyne *rac*-**16**[6] (972 mg, 7.14 mmol) and trimethylaluminum (4.3 mL, 2 M in toluene, 8.6 mmol) in CH_2Cl_2 (15 mL) was added dropwise over 10 min. Subsequently, the solution was stirred at –30 °C for further 15 min, warmed to room temperature, and stirred for 4 h. In a second flask, tris(dibenzylideneacetone)dipalladium(0) (325 mg, 355 µmol) was dissolved in THF (36 mL), and 2-bromopropene (1.04 g, 8.58 mmol) was added dropwise using a water bath for maintaining the temperature at RT, and the mixture was stirred for 20 min. Subsequently, the first solution containing the vinylalane was added dropwise and stirred at reflux for 18 h. After cooling to 0 °C, saturated aqueous $NaHCO_3$ was added. The aqueous layer was extracted three times with EtOAc. The organic layers were combined, washed with brine, dried over $MgSO_4$, and concentrated under vacuum. The residue was purified by flash chromatography (isohexane/EtOAc 8:1) to give *rac*-**17**[6] (580 mg) as a colorless liquid and a mixture of *rac*-**17** (154 mg) and *rac*-**25** (136 mg). The latter mixture was again subjected to flash chromatography (toluene/EtOAc 10:1) to provide *rac*-**17** (114 mg, combined with the first fraction: 114 mg + 580 mg = 694 mg, 3.61 mmol, 51%) and *rac*-**25** (31.6 mg, 0.208 mmol, 3%) as pure compounds and also a mixture of *rac*-**17** and *rac*-**25**, which was discarded.

rac-**25**: colorless liquid; R_f = 0.50 (isohexane/EtOAc 3:1); ^1H NMR (600 MHz, $CDCl_3$): δ = 7.43–7.40 (m, 1 H), 7.39 (t, *J* = 1.7 Hz, 1 H), 6.46–6.40 (m, 1 H), 4.95–4.91 (m, 1 H), 4.88–4.84 (m, 1 H), 4.81 (ddd, *J* = 7.9, 5.5, 2.8 Hz, 1 H), 2.49–2.42 (m, 2 H), 1.97 (d, *J* = 3.0 Hz, 1 H), 1.79 (s, 3 H); ^{13}C NMR (151 MHz, $CDCl_3$): δ = 143.40 (d), 142.14 (s), 139.14 (d), 128.67 (s), 114.29 (t), 108.67 (d), 64.39 (d), 46.90 (t), 22.49 (q); IR (ATR): ν_{max} = 3382, 2932, 2911, 1740, 1501, 1275, 1157, 1021, 960, 874, 791, 733, 664 cm^{-1}; MS (70 eV): *m/z* (%) = 152 (11) $[M]^{\bullet+}$, 137 (6) $[M–CH_3]^+$, 105 (3), 97 (100) [(Furyl)CHOH]$^+$, 95 (15), 69 (48), 55 (6) $[C_4H_7]^+$, 41 (34) [Allyl]$^+$, 39 (29); elemental analysis calcd (%) for $C_9H_{12}O_2$: C 71.03, H 7.95; found: C 71.12, H 7.86.

2.8 *O*-Acylation of 17 to give 5 and subsequent IMDA to give 4

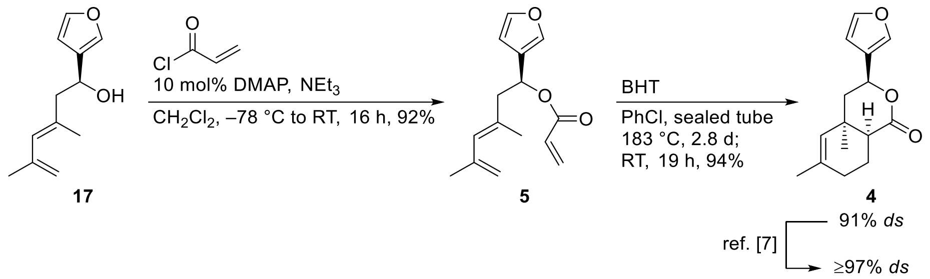

A solution of alcohol **17** (1.37 g, 7.12 mmol) and 4-dimethylaminopyridine (89.8 mg, 0.735 mmol) in CH_2Cl_2 (35 mL) was treated at –78 °C with triethylamine (5.9 mL, 42.6 mmol) and acryloyl chloride (1.21 mL, 14.3 mmol). The solution was stirred for 16 h and then allowed to warm to room temperature slowly. After addition of Et_2O, the mixture was filtered through a short pad of silica gel (elution with Et_2O) and concentrated under vacuum to give triene **5** (1.61 g, 6.54 mmol) as a pale-yellow liquid, which was immediately used for the next step without further purification.

A solution of crude triene **5** (1.61 g, 6.54 mmol) and 2,6-di-*tert*-butyl-4-methylphenol (1.61 g, 6.54 mmol) in chlorobenzene (220 mL) was heated in a sealed tube to 183 °C for 2.8 d and subsequently cooled to room temperature. After concentration under vacuum, flash chromatography over silica gel (isohexane/EtOAc 5:1) afforded lactone **4** (1.66 g, 6.12 mmol, 91% *ds* according to GC analysis, 94% from **5**, 86% over 2 steps from **17**) as a colorless solid. The diastereomeric purity of **4** was increased to *ds* ≥97% by de-/reprotonation with LiHMDS and MeOH according to the literature procedure.[6]

5: ^{13}C NMR (151 MHz, $CDCl_3$): δ = 165.56 (s), 143.30 (d), 141.86 (s), 140.29 (d), 132.43 (s), 130.93 (t), 130.89 (d), 128.70 (d), 124.92 (s), 114.59 (t), 109.12 (d), 67.06 (d), 46.31 (t), 23.63 (q), 18.09 (q), IR (ATR): ν_{max} = 3081, 2964, 2936, 2912, 2853, 1722, 1404, 1187, 1160, 1041, 1023, 983, 893, 873, 803, 769, 730, 664 cm^{-1}; ESI-MS (+10 V): *m/z* = 510.3 $[2M+NH_4]^+$; HRMS (ESI): *m/z* calcd for $C_{30}H_{36}O_6+Na^+$: 515.2404 $[2M+Na]^+$; found: 515.2412; remaining analytical data see ref. [6].

4: m.p. 57–59 °C; $[\alpha]_D^{21}$ = +3.9 (*c* 1.23 in CH_2Cl_2); remaining analytical data see ref. [6].

2.9 Dibromoolefination of keto aldehyde *rac*-28 to give *rac*-29

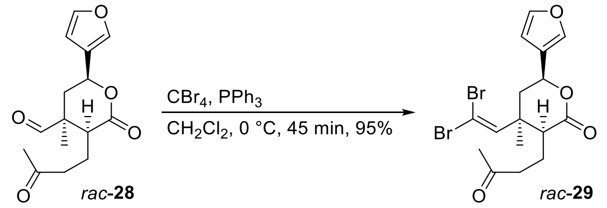

A solution of triphenylphosphine (787 mg, 3.00 mmol) in CH_2Cl_2 (15 mL) was treated with tetrabromomethane (497 mg, 1.50 mmol) at 0 °C and stirred for 1 h. Subsequently, solid *rac*-**28**[7] (210 mg, 0.755 mmol) was added, and the solution was stirred for further 45 min at 0 °C. Then, saturated aqueous NH_4Cl and saturated aqueous $NaHCO_3$ were added, and the aqueous layer was extracted three times with Et_2O. The organic layers were combined, dried over $MgSO_4$, and concentrated under reduced pressure. Purification by flash chromatography (CH_2Cl_2/Et_2O 20:1) afforded dibromoolefin *rac*-**29** (310 mg, 0.714 mmol, 95%) as a colorless oil.

rac-**29**: R_f = 0.62 (CH_2Cl_2/Et_2O 8:1); ^1H NMR (300 MHz, $CDCl_3$): δ = 7.54–7.45 (m, 1 H), 7.42 (t, *J* = 1.7 Hz, 1 H), 6.61 (s, 1 H), 6.45 (dd, *J* = 1.7, 0.8 Hz, 1 H), 5.33 (dd, *J* = 11.9, 3.4 Hz, 1 H), 2.80 (dt, *J* = 18.7, 6.0 Hz, 1 H), 2.65 (dt, *J* = 18.9, 7.4 Hz, 1 H), 2.54 (t, *J* = 6.7 Hz, 1 H), 2.43 (dd, *J* = 14.5, 11.9 Hz, 1 H), 2.26 (dd, *J* = 14.5, 3.4 Hz, 1 H), 2.15 (s, 3 H), 1.95–1.84 (m, 2 H), 1.59 (s, 3 H); ^{13}C NMR (75 MHz, $CDCl_3$): δ = 208.36 (s), 173.04 (s), 143.96 (d), 141.78 (d), 139.98 (d), 123.80 (s), 108.63 (d), 89.79 (s), 70.47 (d), 48.67 (d), 41.59 (s), 41.49 (t), 41.36 (t), 30.25 (q), 25.72 (q), 21.02 (t); IR (ATR): ν_{max} = 3444, 2962, 2935, 1710, 1652, 1160,

1023, 874, 804, 728 cm^{-1}; ESI-MS (+10 V): *m/z* = 433.2, 435.1, 437.1 [*M*+H]$^+$; HRMS (ESI): *m/z* calcd for $C_{16}H_{18}{}^{79}Br_2O_4$+$Na^+$: 454.9464 [*M*+Na]$^+$; found: 454.9463.

2.10 (*E*)-selective Sonogashira coupling of *rac*-29 to give *rac*-30

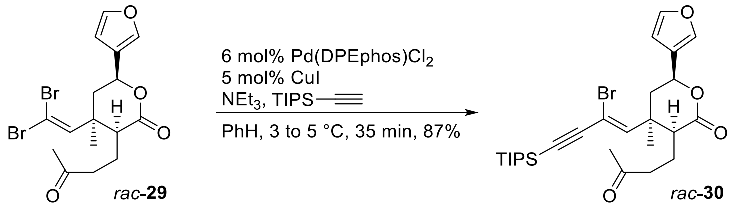

A solution of dibromoolefin *rac*-**29** (181 mg, 417 µmol) in benzene (4 mL) was cooled to 3 °C and treated with copper(I) iodide (3.97 mg, 20.9 µmol), (tri-*iso*-propylsilyl)acetylene (98.9 mg, 542 µmol), triethylamine (84.4 mg, 834 µmol), and dichloro(bis(2-(diphenylphosphino)phenyl)ether)palladium(II) (16.8 mg, 23.5 µmol). After stirring for 35 min at 3 to 5 °C, saturated aqueous NH_4Cl and Et_2O were added. The aqueous layer was extracted three times with Et_2O. The organic layers were combined, dried over $MgSO_4$, and concentrated under vacuum. Purification by flash chromatography (pentane/Et_2O 1:1) afforded enyne *rac*-**30** (194 mg, 362 µmol, 87%) as a colorless wax.

rac-**30**: R_f = 0.49 (pentane/Et_2O 3:7); ^{1}H NMR (300 MHz, $CDCl_3$): δ = 7.53–7.45 (m, 1 H), 7.41 (t, J = 1.7 Hz, 1 H), 6.45 (dd, J = 1.8, 0.7 Hz, 1 H), 6.43 (s, 1 H), 5.34 (dd, J = 11.9, 3.2 Hz, 1 H), 2.90–2.74 (m, 1 H), 2.71–2.57 (m, 2 H), 2.45 (dd, J = 14.6, 11.9 Hz, 1 H), 2.28 (dd, J = 14.5, 3.4 Hz, 1 H), 2.14 (s, 3 H), 2.01–1.81 (m, 2 H), 1.61 (s, 3 H), 1.12–1.03 (m, 21 H); ^{13}C NMR (75 MHz, $CDCl_3$): δ = 208.41 (s), 173.25 (s), 143.88 (d), 142.18 (d), 139.98 (d), 123.88 (s), 108.70 (d), 104.62 (s), 103.14 (s), 93.35 (s), 70.49 (d), 48.73 (d), 41.67 (t), 41.63 (t), 40.26 (s), 30.23 (q), 25.75 (q), 21.03 (t), 18.70 (q), 11.34 (d); IR (ATR): ν_{max} = 2942, 2865, 1739, 1714, 1460, 1368, 1189, 1161, 1070, 1024, 996 , 876, 784, 755, 665 cm^{-1}; ESI-MS (+10 V): *m/z* = 535.3 [*M*+H]$^+$; HRMS (ESI): *m/z* calcd for $C_{27}H_{39}{}^{79}BrO_4Si$+$Na^+$: 557.1693 [*M*+Na]$^+$; found: 557.1694.

2.11 Desilylation of TIPS-alkyne *rac*-30 to give *rac*-31

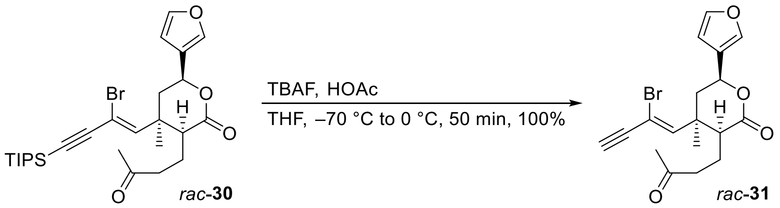

A solution of TIPS-alkyne *rac*-**30** (245 mg, 0.458 mmol) in THF (4.5 mL) was cooled to –70 °C and treated with tetrabutylammonium fluoride (0.68 mL, 1 M in THF, 0.68 mmol) and acetic acid (54 µL, 0.90 mmol). Subsequently, the solution was stirred for 10 min at –70 °C, 10 min at –20 °C, and 30 min at 0 °C. After addition of saturated aqueous NH_4Cl, saturated aqueous $NaHCO_3$, and Et_2O, the aqueous layer was extracted three times with Et_2O. The organic layers were combined, dried over $MgSO_4$, and concentrated under vacuum. Purification by flash chromatography (pentane/Et_2O 3:7) gave terminal alkyne *rac*-**31** (173 mg, 0.457 mmol, 100%) as a colorless oil.

rac-**31**: R_f = 0.26 (pentane/Et_2O 3:7); ^{1}H NMR (500 MHz, $CDCl_3$): δ = 7.52–7.45 (m, 1 H), 7.43 (t, J = 1.7 Hz, 1 H), 6.56 (s, 1 H), 6.45 (d, J = 0.9 Hz, 1 H), 5.35 (dd, J = 12.0, 3.5 Hz, 1 H), 3.10 (s, 1 H), 2.86–2.76 (m, 1 H), 2.70–2.61 (m, 1 H), 2.59 (dd, J = 10.4, 2.8 Hz, 1 H), 2.43 (dd, J = 14.5, 12.0 Hz, 1 H), 2.30 (dd, J = 14.5, 3.5 Hz, 1 H), 2.15 (s, 3 H), 1.97–1.83 (m, 2 H), 1.61 (s, 3 H); ^{13}C NMR (126 MHz, $CDCl_3$): δ = 208.37 (s), 173.10 (s), 144.31 (d), 143.96 (d), 140.01 (d), 123.84 (s), 108.67 (d), 101.92 (s), 82.17 (d), 78.11 (s), 70.47 (d), 48.75 (d), 41.61 (t), 41.50 (t), 40.40 (s), 30.25 (q), 25.66 (q), 21.06 (t); IR (ATR): ν_{max} = 3283, 2969, 2936, 1739, 1710, 1371, 1160, 1061, 1022, 968, 874, 783, 733, 664 cm^{-1}; ESI-MS (+10 V): *m/z* = 379.0 [*M*+H]$^+$, 401.1 [*M*+Na]$^+$; HRMS (ESI): *m/z* calcd for $C_{18}H_{19}{}^{79}BrO_4$+Na^+: 401.0359 [*M*+Na]$^+$; found: 401.0355.

2.12 Semi-reduction of alkyne *rac*-31 to give *rac*-32

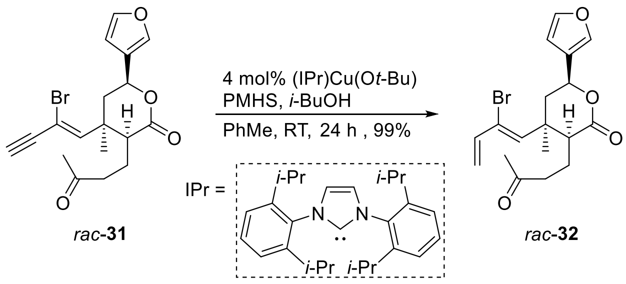

A solution of alkyne *rac*-**31** (173 mg, 0.457 mmol), polymethylhydrosiloxane (41 µL, 0.69 mmol), *iso*-butanol (50.9 mg, 0.687 mmol), and carbene complex (IPr)Cu(O*t*-Bu) (4.7 mg, 8.9 µmol) in toluene (4 mL) was stirred for 5.5 h, subsequently treated again with polymethylhydrosiloxane (41 µL, 0.69 mmol), *iso*-butanol (50.9 mg, 0.687 mmol), and carbene complex (IPr)Cu(O*t*-Bu) (4.8 mg, 9.1 µmol), and stirred for another 17 h. Then, polymethylhydrosiloxane (20 µL, 0.34 mmol) and *iso*-butanol (25.0 mg, 0.337 mmol) were added, and stirring was continued for 105 min. The reaction mixture was filtered over a short pad of silica gel (elution with CH_2Cl_2/Et_2O 4:1). Concentration of the filtrate under reduced pressure and subsequent flash chromatography (CH_2Cl_2/Et_2O 40:1) afforded diene *rac*-**32** (173 mg, 0.454 mmol, 99%) as a colorless wax.

rac-**32**: $R_f = 0.42$ (CH_2Cl_2/Et_2O 40:1); ^{1}H NMR (300 MHz, $CDCl_3$): δ = 7.54–7.45 (m, 1 H), 7.41 (t, J = 1.7 Hz, 1 H), 6.45 (dd, J = 1.7, 0.8 Hz, 1 H), 6.27 (dd, J = 16.3, 10.5 Hz, 1 H), 6.14 (s, 1 H), 5.59 (d, J = 16.2 Hz, 1 H), 5.37 (dd, J = 11.9, 3.4 Hz, 1 H), 5.21 (d, J = 10.4 Hz, 1 H), 2.89–2.73 (m, 1 H), 2.71–2.56 (m, 2 H), 2.55–2.42 (m, 1 H), 2.31 (dd, J = 14.5, 3.4 Hz, 1 H), 2.14 (s, 3 H), 1.95–1.82 (m, 2 H), 1.62 (s, 3 H); ^{13}C NMR (75 MHz, $CDCl_3$): δ = 208.45 (s), 173.51 (s), 143.86 (d), 139.95 (d), 137.78 (d), 136.61 (d), 125.88 (s), 124.03 (s), 118.62 (t), 108.71 (d), 70.61 (d), 49.08 (d), 41.93 (t), 41.76 (t), 39.72 (s), 30.22 (q), 26.32 (q), 21.11 (t); IR (ATR): ν_{max} = 3141, 2962, 2934, 1736, 1711, 1368, 1209, 1190, 1160, 1070, 1023, 969, 912, 874, 796, 733 cm^{-1}; ESI-MS (+10 V): m/z = 381.1, 383.1 $[M+H]^+$; HRMS (APCI): m/z calcd for $C_{18}H_{21}{}^{79}BrO_4+H^+$: 381.0696 $[M+H]^+$; found: 381.0689.

2.13 (*E*)-selective Stille coupling of dibromoolefin *rac*-29 to give *rac*-32

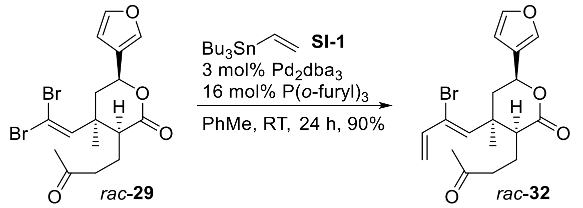

A solution of dibromoolefin *rac*-**29** (338 mg, 778 µmol), tris(dibenzylideneacetone)dipalladium(0) (24.2 mg, 26.4 µmol), tris(2-furyl)phosphine (28.3 mg, 122 µmol), and tributylvinylstannane (**SI-1**; 262 mg, 827 µmol) in toluene (6 mL) was stirred at room temperature for 24 h. The reaction mixture was filtered over a short pad of silica gel (elution with CH_2Cl_2/Et_2O 1:1). Concentration of the filtrate under reduced pressure and subsequent flash chromatography (CH_2Cl_2/Et_2O 20:1) afforded 2-bromo-1,3-diene *rac*-**32** (266 mg, 697 µmol, 90%).

2.14 HWE reaction of ketone *rac*-32 to give *rac*-33 and *rac*-34

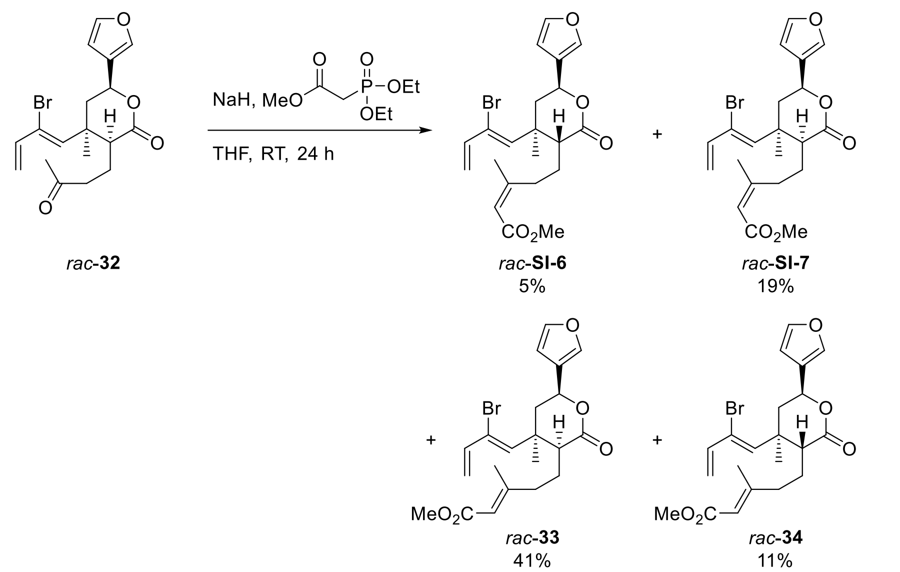

rac-**32**; *rac*-**SI-6** 5%; *rac*-**SI-7** 19%; *rac*-**33** 41%; *rac*-**34** 11%

To a suspension of sodium hydride (76.0 mg, 60 *w*% in mineral oil, 1.90 mmol) and THF (3 mL) was added methyl diethylphosphonoacetate (459 mg, 2.18 mmol) dropwise at 0 °C. After stirring for 25 min at room temperature, a solution of ketone *rac*-**32** (277 mg, 0.727 mmol) in THF (3.6 mL) was added, and the reaction mixture was stirred for 24 h. Subsequently, saturated aqueous NH_4Cl, Et_2O and water were added. The aqueous layer was extracted three times with Et_2O. The organic layers were combined, dried over $MgSO_4$, and concentrated under reduced pressure. The residue was purified by flash chromatography (pentane/Et_2O 3:2 to 1:2) affording the following fractions:

1. fraction: enone *rac*-**SI-6** (15 mg, 0.034 mmol, 5%) as a colorless wax
2. fraction: enone *rac*-**SI-7** (61.6 mg, 0.141 mmol, 19%) as a colorless wax
3. fraction: enone *rac*-**33** (107.8 mg, 0.247 mmol, 34%) as a colorless wax
4. fraction: mixture of enones *rac*-**33** (23.1 mg, 0.0528 mmol, 7%) and *rac*-**34** (33.9 mg, 0.0775 mmol, 11%), separation via semi-preparative HPLC (isohexane/EtOAc 86:14) is possible

rac-**SI-6**: R_f = 0.47 (pentane/Et_2O 1:1); ^{1}H NMR (600 MHz, $CDCl_3$): δ = 7.50–7.44 (m, 1 H), 7.42 (t, J = 1.7 Hz, 1 H), 6.47–6.41 (m, 1 H), 6.38 (dd, J = 16.2, 10.2 Hz, 1 H), 6.36 (s, 1 H), 5.68 (s, 1 H), 5.62 (d, J = 16.2 Hz, 1 H), 5.45 (dd, J = 11.9, 4.0 Hz, 1 H), 5.21 (d, J = 10.5 Hz, 1 H), 3.67 (s, 3 H), 3.12 (td, J = 11.3, 5.3 Hz, 1 H), 2.75 (dd, J = 8.8, 1.7 Hz, 1 H), 2.60–2.53 (m, 2 H), 2.35 (dd, J = 14.1, 11.9 Hz, 1 H), 2.07–1.99 (m, 1 H), 1.95 (d, J = 1.5 Hz, 3 H), 1.68–1.52 (m, 1 H), 1.46 (s, 3 H); ^{13}C NMR (151 MHz, $CDCl_3$): δ = 172.92 (s), 166.81 (s), 160.98 (s), 143.84 (d), 139.86 (d), 139.57 (d), 137.03 (d), 125.10 (s), 125.05 (s), 118.22 (t), 116.14 (d), 108.68 (d), 71.60 (d), 51.95 (d), 50.98 (q), 40.30 (s), 39.53 (t), 34.41 (t), 26.70 (t), 25.72 (q), 19.98 (q); IR (ATR): ν_{max} = 2924, 2852, 1709, 1646, 1437, 1376, 1236, 1203, 1141, 1072, 1021, 975, 917, 873, 853, 795, 731, 695 cm^{-1}; ESI-MS (+75 V): m/z = 437.5, 439.3 [M+H]$^+$, 459.3, 461.2 [M+Na]$^+$; HRMS (ESI): m/z calcd for $C_{21}H_{25}{}^{79}BrO_5$+Na$^+$: 459.0778 [M+Na]$^+$; found: 459.0777.

rac-**SI-7**: R_f = 0.43 (pentane/Et_2O 1:1); ^{1}H NMR (600 MHz, $CDCl_3$): δ = 7.53–7.46 (m, 1 H), 7.42 (t, J = 1.7 Hz, 1 H), 6.45 (dd, J = 1.9, 0.8 Hz, 1 H), 6.26 (dd, J = 16.2, 10.9 Hz, 1 H), 6.16 (s, 1 H), 5.71 (d, J = 1.1 Hz, 1 H), 5.59 (d, J = 16.6 Hz, 1 H), 5.36 (dd, J = 11.3, 3.8 Hz, 1 H), 5.19 (d, J = 10.2 Hz, 1 H), 3.67 (s, 3 H), 2.98 (ddd, J = 11.8, 9.5, 6.2 Hz, 1 H), 2.59 (dd, J = 9.8, 1.9 Hz, 1 H), 2.49–2.39 (m, 3 H), 2.14–2.07 (m, 1 H), 1.95 (d, J = 1.5 Hz, 3 H), 1.68–1.60 (m, 1 H), 1.63 (s, 3 H); ^{13}C NMR (151 MHz, $CDCl_3$): δ = 173.64 (s), 166.86 (s), 159.70 (s), 143.79 (d), 139.98 (d), 138.08 (d), 136.72 (d), 125.91 (s), 124.05 (s), 118.42 (t), 116.75 (d), 108.80 (d), 70.26 (d), 51.09 (q), 50.19 (d), 43.40 (t), 39.89 (s), 33.04 (t), 26.59 (q), 25.42 (q), 25.25 (t); IR (ATR): ν_{max} = 3144, 2946, 2866, 1735, 1709, 1651, 1436, 1283, 1240, 1202, 1160, 1141, 1070, 1022, 969, 914, 874 cm^{-1}; ESI-MS (+25 V): m/z = 437.4, 439.3 [M+H]$^+$; HRMS (ESI): m/z calcd for $C_{21}H_{25}{}^{79}BrO_5$+H$^+$: 437.0958 [M+H]$^+$; found: 437.0957.

rac-**33**: R_f = 0.37 (pentane/Et_2O 1:1); ^{1}H NMR (600 MHz, $CDCl_3$): δ = 7.54–7.45 (m, 1 H), 7.42 (t, J = 1.7 Hz, 1 H), 6.44 (d, J = 0.8 Hz, 1 H), 6.26 (dd, J = 16.0, 10.7 Hz, 1 H), 6.10 (s, 1 H), 5.74–5.68 (m, 1 H), 5.62 (d, J = 16.2 Hz, 1 H), 5.38 (dd, J = 12.0, 3.4 Hz, 1 H), 5.23 (d, J = 10.5 Hz, 1 H), 3.69 (s, 3 H), 2.53 (dd, J = 10.4, 1.7 Hz, 1 H), 2.51–2.43 (m, 2 H), 2.33 (dd, J = 14.5, 3.2 Hz, 1 H), 2.23–2.16 (m, 1 H), 2.18 (d, J = 1.1 Hz, 3 H) 2.06–1.98 (m, 1 H), 1.65 (dddd, J = 13.6, 9.6, 7.2, 2.1 Hz, 1 H), 1.60 (s, 3 H); ^{13}C NMR (126 MHz, $CDCl_3$): δ = 173.08 (s), 167.17 (s), 158.99 (s), 143.89 (d), 139.96 (d), 137.57 (d), 136.52 (d), 126.07 (s), 123.95 (s), 118.87 (t), 116.20 (d), 108.69 (d), 70.42 (d), 51.03 (q), 49.47 (d), 42.78 (t), 39.77 (s), 39.68 (t), 26.45 (q), 24.93 (t), 19.00 (q); IR (ATR): ν_{max} = 3144, 2947, 2866, 1732, 1715, 1651, 1435, 1220, 1146, 1069, 1023, 696, 914, 873, 823, 783 cm^{-1}; ESI-MS (+50 V): *m/z* = 437.3, 439.2 $[M+H]^+$, 454.3, 456.3 $[M+NH_4]^+$, 459.3, 461.1 $[M+Na]^+$; HRMS (ESI): *m/z* calcd for $C_{21}H_{25}{}^{79}BrO_5+H^+$: 437.0958 $[M+H]^+$; found: 437.0960.

rac-**34**: analytical data see section 2.18.

2.15 HWE reaction of ketone *rac*-29 to give *rac*-35 and *rac*-36

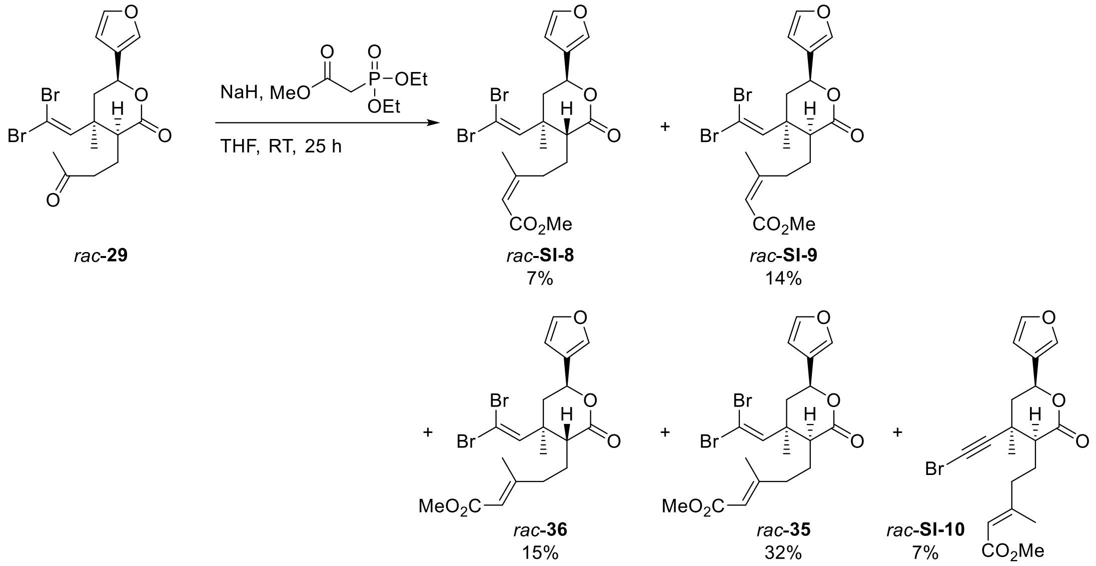

To a suspension of sodium hydride (370.0 mg, 60 *w*% in mineral oil, 9.26 mmol) and THF (10 mL) was added methyl diethylphosphonoacetate (2.23 g, 10.6 mmol) dropwise over a period of 55 min at 0 °C. After stirring for 2 h at room temperature, a solution of ketone *rac*-**29** (1.53 g, 3.53 mmol) in THF (23 mL) was added, and the reaction mixture was stirred for 25 h. Subsequently, saturated aqueous NH_4Cl, EtOAc, and water were added. The aqueous layer was extracted three times with EtOAc. The organic layers were combined, dried over $MgSO_4$, and concentrated under reduced pressure. The residue was purified by flash chromatography (pentane/Et_2O 6:3 to 6:4 to 6:6) affording the following fractions:

1. fraction:[1] mixture of enones *rac*-**SI-8** (126 mg, 0.257 mmol, 7%) and *rac*-**SI-9** (244 mg, 0.498 mmol, 14%)
2. fraction: enone *rac*-**36** (256 mg, 0.522 mmol, 15%)
3. fraction: mixture of enone *rac*-**35** (556 mg, 1.13 mmol, 32%) and bromo alkyne *rac*-**SI-10** (107 mg, 0.261 mmol, 7%)

Fractions 1 and 2 also contained small amounts of byproducts, probably several isomeric bromo alkynes.

rac-**SI-8**: colorless wax; R_f = 0.45 (pentane/Et_2O 3:2); ^{1}H NMR (600 MHz, $CDCl_3$): δ = 7.49–7.47 (m, 1 H), 7.44–7.42 (m, 1 H), 6.81–6.78 (m, 1 H), 6.45–6.42 (m, 1 H), 5.73–5.68 (m, 1 H), 5.40 (dd, J = 11.7, 4.1 Hz, 1 H), 3.70 (s, 3 H), 3.12 (dt, J = 11.1, 5.3 Hz, 1 H), 2.69 (dd, J = 9.0, 1.9 Hz, 1 H), 2.58 (dt, J = 11.3, 5.3 Hz, 1 H), 2.46–2.41 (m, 1 H), 2.33 (dd, J = 14.1, 11.9 Hz, 1 H), 2.06–1.99 (m, 1 H), 1.97 (d, J = 1.1 Hz, 3 H), 1.58–

1 In this specific experiment, *rac*-**SI-8** and *rac*-**SI-9** were not collected separately, since both isomers cannot be further applied in our synthesis. However, separation is easily achieved by flash chromatography as described above and has been done once for characterization of both compounds.

1.51 (m, 1 H), 1.40 (s, 3 H); ^{13}C NMR (151 MHz, $CDCl_3$): δ = 172.37 (s), 166.77 (s), 160.70 (s), 143.94 (d), 143.24 (d), 139.87 (d), 124.87 (s), 116.37 (d), 108.59 (d), 88.94 (s), 71.33 (d), 51.28 (d), 51.13 (q), 42.16 (s), 39.16 (t), 34.16 (t), 26.70 (t), 25.77 (q), 19.39 (q); IR (ATR): ν_{max} = 2967, 2953, 2912, 2850, 2055, 2030, 2009, 1725, 1705, 1635, 1439, 1374, 1355, 1209, 1149, 1074, 1021, 943, 873, 803 cm^{-1}; ESI-MS (+10 V): *m/z* = 489.2, 491.2, 493.1 $[M+H]^+$, 506.3, 508.3, 510.2 $[M+NH_4]^+$; HRMS (APCI): *m/z* calcd for $C_{19}H_{22}{}^{79}Br_2O_5+H^+$: 488.9907 $[M+H]^+$; found: 488.9914.

rac-**SI-9**: colorless wax; R_f = 0.39 (pentane/Et_2O 3:2); ^{1}H NMR (600 MHz, $CDCl_3$): δ = 7.53–7.48 (m, 1 H), 7.43 (t, *J* = 1.7 Hz, 1 H), 6.61 (s, 1 H), 6.45 (d, *J* = 1.1 Hz, 1 H), 5.73 (s, 1 H), 5.33 (dd, *J* = 11.9, 3.2 Hz, 1 H), 3.67 (s, 3 H), 2.97 (ddd, *J* = 11.9, 9.6, 6.4 Hz, 1 H), 2.53 (dd, *J* = 9.8, 1.5 Hz, 1 H), 2.47–2.43 (m, 1 H), 2.40 (dd, *J* = 14.3, 11.7 Hz, 1 H), 2.32 (dd, *J* = 14.7, 3.0 Hz, 1 H), 2.12–2.05 (m, 1 H), 1.97 (d, *J* = 1.1 Hz, 3 H), 1.66–1.60 (m, 1 H), 1.58 (s, 3 H); ^{13}C NMR (151 MHz, $CDCl_3$): δ = 173.10 (s), 166.86 (s), 159.56 (s), 143.89 (d), 142.04 (d), 139.99 (d), 123.81 (s), 116.83 (d), 108.70 (d), 89.81 (s), 70.10 (d), 51.12 (q), 49.79 (d), 42.75 (t), 41.70 (s), 32.94 (t), 25.91 (q), 25.44 (q), 25.19 (t); IR (ATR): ν_{max} = 3144, 2930, 2846, 1739, 1709, 1649, 1436, 1376, 1200, 1142, 1070, 1022, 918, 874, 805, 730 cm^{-1}; ESI-MS (+10 V): *m/z* = 489.2, 491.1, 493.1 $[M+H]^+$, 506.3, 508.2, 510.2 $[M+NH_4]^+$; HRMS (APCI): *m/z* calcd for $C_{19}H_{22}{}^{79}Br_2O_5+H^+$: 488.9907 $[M+H]^+$; found: 488.9915.

rac-**36**: colorless wax; R_f = 0.30 (pentane/Et_2O 3:2); ^{1}H NMR (600 MHz, $CDCl_3$): δ = 7.48–7.45 (m, 1 H), 7.44–7.42 (m, 1 H), 6.50 (s, 1 H), 6.43 (dd, *J* = 1.9, 0.8 Hz, 1 H), 5.75–5.69 (m, 1 H), 5.41 (dd, *J* = 11.7, 4.1 Hz, 1 H), 3.70 (s, 3 H), 2.73 (dd, *J* = 9.6, 1.3 Hz, 1 H), 2.66 (ddd, *J* = 14.2, 9.3, 4.7 Hz, 1 H), 2.47 (dd, *J* = 14.3, 11.7 Hz, 1 H), 2.35–2.30 (m, 1 H), 2.27 (dd, *J* = 14.3, 4.1 Hz, 1 H), 2.20 (d, *J* = 1.5 Hz, 3 H), 2.05–1.98 (m, 1 H), 1.57–1.52 (m, 1 H), 1.38 (s, 3 H); ^{13}C NMR (151 MHz, $CDCl_3$): δ = 172.14 (s), 167.17 (s), 159.21 (s), 144.01 (d), 142.61 (d), 139.85 (d), 124.73 (s), 116.33 (d), 108.54 (d), 89.31 (s), 71.29 (d), 51.03 (q), 48.89 (d), 42.10 (s), 40.60 (t), 39.15 (t), 25.52 (t), 20.71 (q), 19.04 (q); IR (ATR): ν_{max} = 3147, 2946, 2853, 1711, 1651, 1435, 1220, 1144, 1072, 1023, 873, 802, 732, 689 cm^{-1}; ESI-MS (+10 V): *m/z* = 506.2, 508.1, 510.1 $[M+NH_4]^+$; HRMS (ESI): *m/z* calcd for $C_{19}H_{22}{}^{79}Br_2O_5+H^+$: 488.9907 $[M+H]^+$; found: 488.9913.

rac-**35**: colorless wax; R_f = 0.24 (pentane/Et_2O 3:2); ^{1}H NMR (600 MHz, $CDCl_3$): δ = 7.50–7.48 (m, 1 H), 7.44–7.42 (m, 1 H), 6.56 (s, 1 H), 6.45–6.42 (m, 1 H), 5.73–5.71 (m, 1 H), 5.34 (dd, *J* = 11.9, 3.2 Hz, 1 H), 3.69 (s, 3 H), 2.47 (ddd, *J* = 14.2, 9.7, 4.7 Hz, 1 H), 2.44–2.38 (m, 1 H), 2.40 (dd, *J* = 14.7, 11.9 Hz, 1 H), 2.26 (dd, *J* = 14.7, 3.0 Hz, 1 H), 2.22–2.15 (m, 1 H), 2.19 (s, 3 H), 2.06–1.98 (m, 1 H), 1.69–1.62 (m, 1 H), 1.54 (s, 3 H); ^{13}C NMR (151 MHz, $CDCl_3$): δ = 172.53 (s), 167.12 (s), 158.67 (s), 143.99 (d), 141.63 (d), 139.97 (d), 123.71 (s), 116.39 (d), 108.58 (d), 90.04 (s), 70.27 (d), 51.06 (q), 49.05 (d), 42.09 (t), 41.57 (s), 39.53 (t), 25.79 (q), 24.85 (t), 18.97 (q); IR (ATR): ν_{max} = 3147, 2947, 1736, 1710, 1651, 1435, 1220, 1145, 1069, 1023, 873, 805, 733 cm^{-1}; ESI-MS (+25 V): *m/z* = 506.4, 508.2, 510.2 $[M+NH_4]^+$; HRMS (ESI): *m/z* calcd for $C_{19}H_{22}{}^{79}Br_2O_5+H^+$: 488.9907 $[M+H]^+$; found: 488.9908.

rac-**SI-10**: analytical data see section 2.17.

2.16 Epimerization of lactone *rac*-35 to give *rac*-36

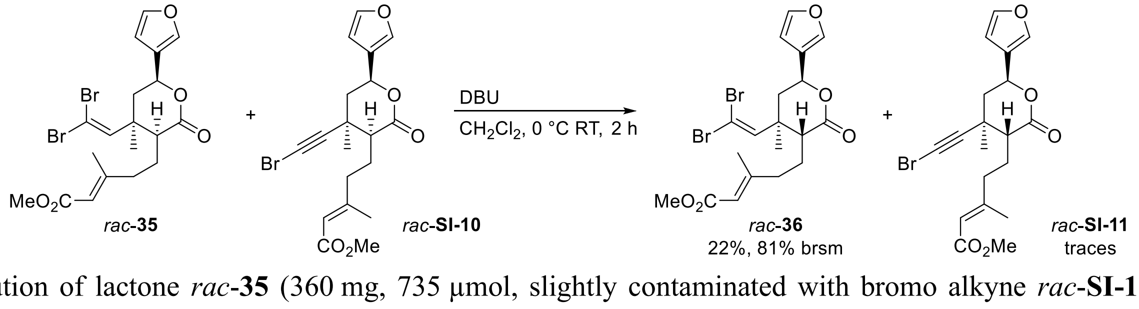

A solution of lactone *rac*-**35** (360 mg, 735 µmol, slightly contaminated with bromo alkyne *rac*-**SI-10**, see section 2.15) in CH_2Cl_2 (8.5 mL) was treated with 1,8-diazabicyclo[5.4.0]undec-7-ene (317 mg, 2.08 mmol) at 0 °C and stirred for 2 h at room temperature. The mixture was filtered over a short pad of silica gel (elution

with Et_2O), and the filtrate was concentrated under vacuum. Flash chromatography (pentane/Et_2O 2:1) of the residue afforded the following fractions:

1. fraction: traces of bromo alkyne *rac*-**SI-11**, collected from several runs for characterization
2. fraction: lactone *rac*-**36** (79.1 mg, 161 µmol, 22%, 81% brsm)
3. fraction: lactone *rac*-**35** (265 mg, 540 µmol, 73%, still contaminated with bromo alkyne *rac*-**SI-10**)

rac-**SI-11**: colorless solid; m.p. 88–90 °C; R_f = 0.51 (pentane/Et_2O 1:1); ^{1}H NMR (600 MHz, $CDCl_3$): δ = 7.48–7.46 (m, 1 H), 7.42 (t, J = 1.7 Hz, 1 H), 6.43 (dd, J = 1.9, 1.1 Hz, 1 H), 5.73 (q, J = 1.1 Hz, 1 H), 5.38 (dd, J = 11.5, 4.3 Hz, 1 H), 3.69 (s, 3 H), 2.61 (dddd, J = 13.9, 8.4, 4.9, 0.8 Hz, 1 H), 2.45 (dd, J = 9.6, 2.1 Hz, 1 H), 2.43–2.37 (m, 1 H), 2.39 (dd, J = 14.3, 11.7 Hz, 1 H), 2.28 (dd, J = 14.3, 4.3 Hz, 1 H), 2.19 (d, J = 1.1 Hz, 3 H), 2.05–1.98 (m, 1 H), 1.95–1.88 (m, 1 H), 1.35 (s, 3 H); ^{13}C NMR (151 MHz, $CDCl_3$): δ = 171.16 (s), 167.23 (s), 159.24 (s), 144.00 (d), 139.94 (d), 124.44 (s), 116.28 (d), 108.56 (d), 83.82 (s), 71.14 (d), 50.97 (q), 49.22 (d), 42.75 (t), 42.51 (s), 40.19 (t), 36.09 (s), 25.10 (t), 21.13 (q), 18.78 (q); IR (ATR): ν_{max} = 2946, 2056, 1734, 1704, 1652, 1436, 1377, 1313, 1208, 1148, 1073, 1023, 954, 892, 874, 800, 782, 733, 693 cm^{-1}; ESI-MS (+10 V): m/z = 426.4, 428.3 $[M+NH_4]^+$; elemental analysis calcd (%) for $C_{19}H_{21}BrO_5$: C 55.76, H 5.17; found: C 56.13, H 5.29.

rac-**35** and *rac*-**36**: analytical data see section 2.15.

2.17 Epimerization of bromo alkyne *rac*-SI-11 to give *rac*-SI-10

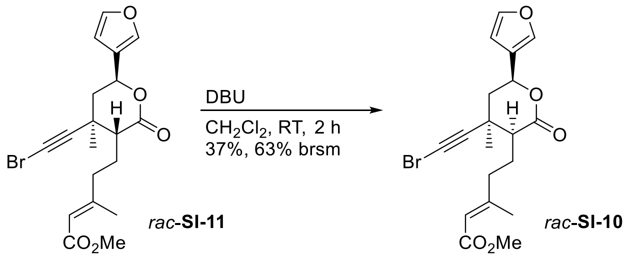

This reaction was carried out for analytical characterization of *rac*-**SI-10**: A solution of bromo alkyne *rac*-**SI-11** (17.2 mg, 42.0 µmol, collected from several runs of epimerization as described in section 2.16) and 1,8-diazabicyclo[5.4.0]undec-7-ene (13.2 mg, 86.7 µmol) in CH_2Cl_2 (1 mL) was stirred for 2 h at room temperature. The mixture was filtered over a short pad of silica gel (elution with Et_2O), and the filtrate was concentrated under vacuum. Flash chromatography (pentane/Et_2O 2:1) of the residue afforded unreacted starting material *rac*-**SI-11** (7.1 mg, 17 µmol, 41%) and bromo alkyne *rac*-**SI-10** (6.3 mg, 15 µmol, 37%, 63% brsm) as a colorless wax.

rac-**SI-10**: R_f = 0.26 (pentane/Et_2O 3:2); ^{1}H NMR (600 MHz, $CDCl_3$): δ = 7.50–7.47 (m, 1 H), 7.43 (t, J = 1.7 Hz, 1 H), 6.45–6.42 (m, 1 H), 5.73 (q, J = 1.1 Hz, 1 H), 5.30 (dd, J = 11.7, 3.0 Hz, 1 H), 3.70 (s, 3 H), 2.51 (ddd, J = 14.0, 9.1, 4.3 Hz, 1 H), 2.42 (dd, J = 14.7, 12.0 Hz, 1 H), 2.27–2.21 (m, 2 H), 2.20 (d, J = 1.1 Hz, 3 H), 2.18 (dd, J = 14.5, 3.2 Hz, 1 H), 2.16–2.10 (m, 1 H), 1.88–1.81 (m, 1 H), 1.50 (s, 3 H); ^{13}C NMR (151 MHz, $CDCl_3$): δ = 171.94 (s), 167.23 (s), 159.04 (s), 143.99 (d), 140.07 (d), 123.49 (s), 116.29 (d), 108.62 (d), 82.63 (s), 69.84 (d), 51.05 (q), 48.19 (d), 43.68 (t), 43.57 (s), 39.21 (t), 35.18 (s), 28.50 (q), 25.41 (t), 18.92 (q); IR (ATR): ν_{max} = 2961, 2922, 2852, 1710, 1259, 1223, 1144, 1071, 1021, 873, 797, 734, 698 cm^{-1}; ESI-MS (+25 V): m/z = 431.8, 433.0 $[M+Na]^+$; HRMS (ESI): m/z calcd for $C_{19}H_{21}{}^{79}BrO_5+Na^+$: 431.0465 $[M+Na]^+$; found: 431.0469.

2.18 (*E*)-selective Stille coupling of dibromoolefin *rac*-36 to give *rac*-34

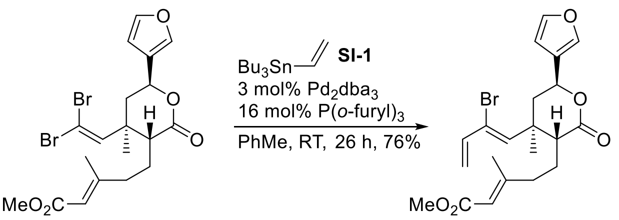

rac-**36** *rac*-**34**

Dibromoolefin *rac*-**36** (220 mg, 448 µmol), obtained by combining the products described in chapters 2.15 and 2.16, was dissolved in toluene (3 mL) and treated with tris(dibenzylideneacetone)dipalladium(0) (12.6 mg, 13.8 µmol), tris(2-furyl)phosphine (16.7 mg, 71.9 µmol), and tributylvinylstannane (**SI-1**; 153 mg, 483 µmol). After stirring for 26 h at room temperature, the reaction mixture was filtered over a short pad of silica gel (elution with CH_2Cl_2/Et_2O 1:1) and concentrated under reduced pressure. The residue was purified twice by flash chromatography (1. column: 100% pentane to pentane/Et_2O 1:1; 2. column pentane/Et_2O 3:2 + 5% triethylamine) to afford 2-bromo-1,3-diene *rac*-**34** (149 mg, 340 µmol, 76%) as a colorless wax.

rac-**34**: R_f = 0.35 (pentane/Et_2O 1:1); ^{1}H NMR (300 MHz, $CDCl_3$): δ = 7.50–7.45 (m, 1 H), 7.42 (t, J = 1.7 Hz, 1 H), 6.44 (dd, J = 1.7, 0.8 Hz, 1 H), 6.28 (dd, J = 16.2, 10.4 Hz, 1 H), 5.99 (s, 1 H), 5.69 (q, J = 1.1 Hz, 1 H), 5.64 (d, J = 16.1 Hz, 1 H), 5.44 (dd, J = 11.7, 4.2 Hz, 1 H), 5.25 (d, J = 10.2 Hz, 1 H), 3.69 (s, 3 H), 2.95 (dd, J = 9.4, 1.3 Hz, 1 H), 2.74–2.59 (m, 1 H), 2.59 (dd, J = 14.2, 11.7 Hz, 1 H), 2.36–2.23 (m, 1 H), 2.29 (dd, J = 14.2, 4.2 Hz, 1 H), 2.16 (d, J = 1.1 Hz, 3 H), 2.00 (dtd, J = 13.9, 9.3, 4.9 Hz, 1 H), 1.59–1.49 (m, 1 H), 1.42 (s, 3 H); ^{13}C NMR (75 MHz, $CDCl_3$): δ = 172.73 (s), 167.21 (s), 159.57 (s), 143.89 (d), 139.84 (d), 138.55 (d), 136.66 (d), 125.27 (s), 124.97 (s), 119.06 (t), 116.07 (d), 108.64 (d), 71.51 (d), 50.99 (q), 49.23 (d), 40.69 (t), 40.18 (s), 39.45 (t), 25.58 (t), 21.61 (q), 19.06 (q); IR (ATR): ν_{max} = 2946, 1710, 1651, 1435, 1221, 1145, 1071, 1023, 972, 947, 917, 873, 794, 732, 696 cm^{-1}; ESI-MS (+25 V): *m/z* = 437.5, 439.4 [*M*+H]$^+$, 454.4, 456.2 [*M*+NH$_4$]$^+$; HRMS (APCI): *m/z* calcd for $C_{21}H_{25}{}^{81}BrO_5$+H$^+$: 439.0938 [*M*+H]$^+$; found: 439.0943.

2.19 IMDA of triene *rac*-33 to give *rac*-37

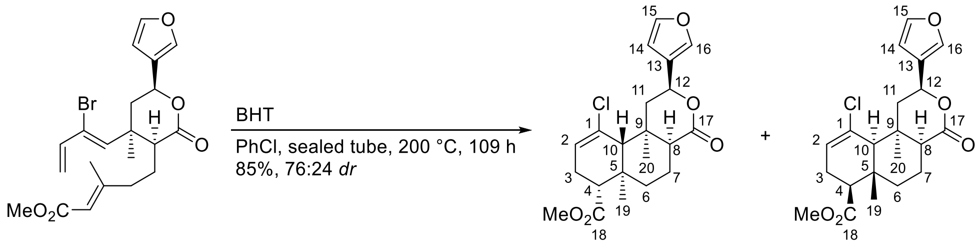

rac-**33** *rac*-**37** *rac*-**38**

A solution of triene *rac*-**33** (10.3 mg, 23.6 µmol) and 2,6-di-*tert*-butyl-4-methylphenol (74.8 mg, 339 µmol) in chlorobenzene (12 mL) was degassed through two freeze-pump-thaw cycles and then heated in a sealed tube to 200 °C for 109 h (4.5 d). After cooling to room temperature and removal of the solvent under vacuum, quick flash chromatography over silica gel (pentane/Et_2O 1:1) afforded a mixture of cycloadducts *rac*-**37** and *rac*-**38** (7.9 mg, 20 µmol, 85%, *dr* 76:24 according to ^{1}H NMR integration). For full characterization, both compounds can be further purified by flash chromatography to give *rac*-**37** and *rac*-**38** with 92% and 74% *ds* as colorless waxes, respectively.

rac-**37**: R_f = 0.54 (pentane/Et_2O 1:2); ^{1}H NMR (600 MHz, $CDCl_3$): δ = 7.49–7.47 (m, 1 H, H-16), 7.43–7.41 (m, 1 H, H-15), 6.46–6.42 (m, 1 H, H-14), 6.01–5.95 (m, 1 H, H-2), 5.24 (d, J = 12.0 Hz, 1 H, H-12), 3.64 (s, 3 H, OC***H***$_3$), 2.65 (dd, J = 15.1, 1.9 Hz, 1 H, H-11α), 2.58–2.55 (m, 1 H, H-8), 2.51–2.45 (m, 2 H, H-3α/β, H-4), 2.42–2.39 (m, 1 H, H-10), 2.22–2.15 (m, 2 H, H-3α/β, H-7β), 2.09 (dd, J = 14.9, 12.2 Hz, 1 H, H-11β), 1.97–1.88 (m, 1 H, H-7α), 1.87 (ddd, J = 13.9, 13.6, 3.2 Hz, 1 H, H-6β), 1.48 (s, 3 H, H-20), 1.40–1.35 (m, 1 H, H-6α), 1.20 (s, 3 H, H-19); ^{13}C NMR (151 MHz, $CDCl_3$): δ = 174.23 (s, C-17), 174.01 (s, C-18), 143.76 (d, C-15), 139.93 (d, C-16), 131.33 (s, C-1), 128.86 (d, C-2), 124.05 (s, C-13), 108.78 (d, C-14), 69.66 (d, C-12), 58.42 (d, C-10), 51.60 (q, O*C*H$_3$), 51.54 (d, C-4) (C-4 and O*C*H$_3$ might be interchanged), 51.51 (t, C-11), 45.91

(d, C-8), 38.52 (s, C-5), 37.37 (s, C-9), 32.59 (t, C-6), 27.62 (t, C-3), 24.96 (q, C-20), 17.93 (t, C-7), 13.50 (q, C-19); IR (ATR): ν_{max} = 2924, 2852, 1727, 1437, 1366, 1322, 1208, 1163, 1096, 1068, 1012, 995, 968, 927, 825 cm^{-1}; ESI-MS (+25 V): *m/z* = 393.6, 395.5 [M+H]$^+$, 410.4, 412.4 [M+NH$_4$]$^+$; HRMS (ESI): *m/z* calcd for $C_{21}H_{25}{}^{35}ClO_5+H^+$: 393.1463 [*M*+H]$^+$; found: 393.1470.

rac-**38**: R_f = 0.43 (pentane/Et$_2$O 1:2); ^{1}H NMR (600 MHz, CDCl$_3$): δ = 7.49–7.47 (m, 1 H, H-16), 7.42 (t, *J* = 1.7 Hz, 1 H, H-15), 6.43 (dd, *J* = 1.9, 0.8 Hz, 1 H, H-14), 5.98–5.95 (m, 1 H, H-2), 5.40 (dd, *J* = 12.2, 3.6 Hz, 1 H, H-12), 3.67 (s, 3 H, OC***H***$_3$), 2.53–2.44 (m, 3 H, H-3β, H-4, H-11α), 2.42–2.37 (m, 2 H, H-8, H-10), 2.28–2.21 (m, 1 H, H-3α), 2.21 (dd, *J* = 14.1, 12.2 Hz, 1 H, H-11β), 1.99 (dq, *J* = 13.9, 4.3 Hz, 1 H, H-7α), 1.95–1.89 (m, 1 H, H-7β), 1.64–1.60 (m, 1 H, H-6β), 1.60 (s, 3 H, H-20), 1.52–1.46 (m, 1 H, H-6α), 1.20 (s, 3 H, H-19); ^{13}C NMR (151 MHz, CDCl$_3$): δ = 173.75 (s, C-17), 173.64 (s, C-18), 143.82 (d, C-15), 139.63 (d, C-16), 131.16 (s, C-1), 128.62 (d, C-2), 125.33 (s, C-13), 108.58 (d, C-14), 72.43 (d, C-12), 57.82 (d, C-10), 52.50 (d, C-8), 52.27 (d, C-4), 51.74 (q, O***C***H$_3$), 38.30 (s, C-5), 36.51 (s, C-9), 36.03 (t, C-6), 32.63 (t, C-11), 30.98 (q, C-20), 27.75 (t, C-3), 23.46 (t, C-7), 14.65 (q, C-19); IR (ATR): ν_{max} = 3395, 2923, 2852, 1725, 1652, 1458, 1437, 1370, 1250, 1211, 1165, 1117, 1069, 1010, 985, 934 cm^{-1}; ESI-MS (+25 V): *m/z* = 393.6, 395.4 [M+H]$^+$; HRMS (APCI): *m/z* calcd for $C_{21}H_{25}{}^{37}ClO_5+H^+$: 395.1434 [*M*+H]$^+$; found: 395.1428.

2.20 IMDA of triene *rac*-34 to give *rac*-7 and *rac*-8

Example 1:

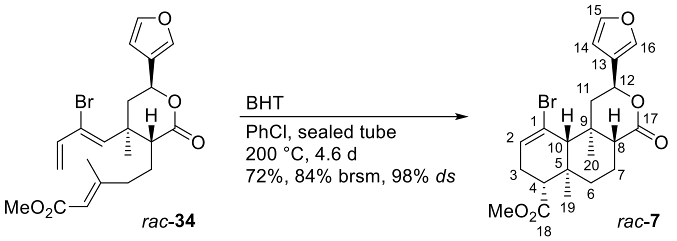

A solution of triene *rac*-**34** (18.3 mg, 41.9 µmol) and 2,6-di-*tert*-butyl-4-methylphenol (131 mg, 595 µmol) in chlorobenzene (20 mL) was filtered over sand and transferred into a sealed tube. The reaction mixture was degassed through two freeze-pump-thaw cycles and heated for 111 h (4.6 d) at 200 °C. After cooling to room temperature, the solvents were removed under reduced pressure, and the residue was purified twice by flash chromatography (1. column: isohexane/EtOAc 5:2; 2. column: isohexane/EtOAc 4:1) to afford unreacted starting material *rac*-**34** (2.7 mg, 6.0 µmol, 14%) and vinyl bromide *rac*-**7** (13.2 mg, 30.2 µmol, 72%, 84% brsm, 98% *ds* according to GC analysis) as a colorless solid. GC analysis of the product showed traces of vinyl chloride *rac*-**8**.

rac-**7**: R_f = 0.36 (pentane/Et$_2$O 1:1); m.p. 225–227 °C; ^{1}H NMR (600 MHz, CDCl$_3$): δ = 7.45–7.43 (m, 1 H, H-16), 7.42 (t, *J* = 1.7 Hz, 1 H, H-15), 6.42 (dd, *J* = 1.7, 0.9 Hz, 1 H, H-14), 6.32–6.26 (m, 1 H, H-2), 5.50 (dd, *J* = 10.4, 5.8 Hz, 1 H, H-12), 3.76 (dd, *J* = 14.1, 5.8 Hz, 1 H, H-11α), 3.66 (s, 3 H, OC***H***$_3$), 2.53 (dd, *J* = 11.8, 5.0 Hz, 1 H, H-4), 2.50–2.42 (m, 2 H, H-3α, H-10), 2.24–2.18 (m, 2 H, H-3β, H-8), 2.06 (dq, *J* = 14.5, 3.6 Hz, 1 H, H-7β), 1.78–1.70 (m, 1 H, H-7α), 1.75 (dd, *J* = 14.1, 10.7 Hz, 1 H, H-11β), 1.59 (dt, *J* = 13.6, 3.4 Hz, 1 H, H-6α), 1.38 (td, *J* = 13.6, 3.4 Hz, 1 H, H-6β), 1.32 (s, 3 H, H-20), 1.22 (s, 3 H, H-19); ^{13}C NMR (151 MHz, CDCl$_3$): δ = 173.61 (s, C-18), 172.17 (s, C-17), 143.94 (d, C-15), 139.47 (d, C-16), 133.91 (d, C-2), 126.11 (s, C-13), 121.14 (s, C-1), 108.70 (d, C-14), 71.17 (d, C-12), 58.64 (d, C-10), 52.70 (d, C-8), 52.44 (d, C-4), 51.71 (q, O***C***H$_3$), 45.29 (t, C-11), 38.96 (s, C-5), 38.24 (s, C-9), 36.72 (t, C-6), 29.52 (t, C-3), 18.10 (t, C-7), 15.53 (q, C-20), 14.33 (q, C-19); IR (ATR): ν_{max} = 3139, 2920, 2852, 1725, 1652, 1434, 1377, 1251, 1219, 1152, 1071, 1020, 1002, 933, 872, 820, 789, 739, 729, 692, 627 cm^{-1}; ESI-MS (+25 V): *m/z* = 437.5, 439.3 [*M*+H]$^+$; HRMS (ESI): *m/z* calcd for $C_{21}H_{25}{}^{79}BrO_5+Na^+$: 459.0778 [*M*+Na]$^+$; found: 459.0769.

Example 2:

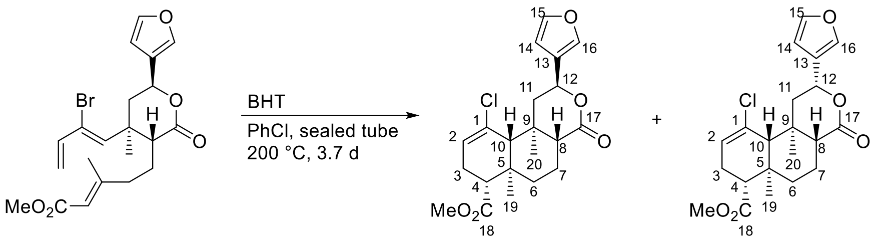

rac-**34** *rac*-**8** 44%, 90% purity *rac*-**39** 14%, >99% *ds*

Experimental procedure analogous to example 1: Heating of *rac*-**34** (149 mg, 340 µmol) and 2,6-di-*tert*-butyl-4-methylphenol (1.06 g, 4.81 mmol) in chlorobenzene (124 mL) at 200 °C for 88 h (3.7 d) afforded two isomeric vinyl chlorides *rac*-**8** (59.2 mg, 151 µmol, 44%, 90% purity according to ^{1}H NMR integration) and *rac*-**36** (18.5 mg, 47.1 µmol, 14%, >99% *ds* according to GC analysis) as colorless solids.

rac-**8**: R_f = 0.53 (pentane/Et$_2$O 1:1); m.p. 163–165 °C; ^{1}H NMR (600 MHz, CDCl$_3$): δ = 7.44–7.43 (m, 1 H, H-16), 7.42 (t, J = 1.7 Hz, 1 H, H-15), 6.41 (dd, J = 1.9, 0.8 Hz, 1 H, H-14), 5.96 (dt, J = 5.0, 2.6 Hz, 1 H, H-2), 5.50 (dd, J = 10.2, 6.0 Hz, 1 H, H-12), 3.67 (s, 3 H, OC***H***$_3$), 3.54 (dd, J = 14.3, 6.0 Hz, 1 H, H-11α), 2.54–2.47 (m, 2 H, H-3α, H-4), 2.38–2.35 (m, 1 H, H-10), 2.28–2.20 (m, 1 H, H-3β), 2.23 (dd, J = 12.6, 4.0 Hz, 1 H, H-3β, H-8), 2.07 (dq, J = 14.6, 3.4 Hz, 1 H, H-7β), 1.80 (dd, J = 13.9, 10.5 Hz, 1 H, H-11β), 1.75 (dddd, J = 14.6, 13.2, 12.4, 3.4 Hz, 1 H, H-7α), 1.59 (dt, J = 13.6, 3.4 Hz, 1 H, H-6α), 1.37 (ddd, J = 13.6, 13.2, 3.8 Hz, 1 H, H-6β), 1.30 (s, 3 H, H-20), 1.21 (s, 3 H, H-19); ^{13}C NMR (151 MHz, CDCl$_3$): δ = 173.62 (s, C-18), 172.16 (s, C-17), 143.95 (d, C-15), 139.47 (d, C-16), 131.45 (s, C-1), 128.69 (d, C-2), 126.16 (s, C-13), 108.71 (d, C-14), 71.28 (d, C-12), 58.35 (d, C-10), 52.44 (d, C-8), 52.39 (d, C-4) (C-4 and C-8 might be interchanged), 51.71 (q, O***C***H$_3$), 45.69 (t, C-11), 38.45 (s, C-5), 37.98 (s, C-9), 36.62 (t, C-6), 27.86 (t, C-3), 18.23 (t, C-7), 15.70 (q, C-20), 14.33 (q, C-19); IR (ATR): $\nu_{\max}$ = 2950, 2919, 2843, 1725, 1434, 1370, 1220, 1160, 1080, 1020, 875, 787, 735 cm^{-1}; ESI-MS (+25 V): *m/z* = 393.9, 395.5 [*M*+H]$^+$, 410.5, 412.4 [*M*+NH$_4$]$^+$; HRMS (ESI): *m/z* calcd for $C_{21}H_{25}{}^{35}ClO_5$+Na$^+$: 415.1283 [*M*+Na]$^+$; found: 415.1278.

rac-**39**: R_f = 0.30 (pentane/Et$_2$O 1:1); m.p. 167–169 °C; ^{1}H NMR (600 MHz, CDCl$_3$): δ = 7.48–7.44 (m, 1 H, H-16), 7.42 (t, J = 1.7 Hz, 1 H, H-15), 6.45–6.42 (m, 1 H, H-14), 5.97 (dt, J = 5.0, 2.6 Hz, 1 H, H-2), 5.30 (dd, J = 11.3, 6.0 Hz, 1 H, H-12), 3.68 (s, 3 H, OC***H***$_3$), 2.65–2.58 (m, 2 H, H-8, H-10), 2.55–2.49 (m, 2 H, H-3β, H-4), 2.46 (dd, J = 14.7, 11.3 Hz, 1 H, H-11β), 2.39 (dd, J = 14.7, 6.0 Hz, 1 H, H-11α), 2.27–2.18 (m, 1 H, H-3α), 1.97–1.89 (m, 2 H, H-7α, H-7β), 1.61 (dt, J = 13.6, 3.4 Hz, 1 H, H-6β), 1.48–1.40 (m, 1 H, H-6α), 1.24 (s, 3 H, H-20), 1.17 (s, 3 H, H-19); ^{13}C NMR (151 MHz, CDCl$_3$): δ = 173.94 (s, C-17), 173.61 (s, C-18), 143.86 (d, C-15), 139.76 (d, C-16), 131.52 (s, C-1), 128.21 (d, C-2), 124.47 (s, C-13), 108.84 (d, C-14), 70.63 (d, C-12), 60.02 (d, C-10), 52.17 (d, C-4), 51.69 (q, O***C***H$_3$), 48.79 (t, C-11), 48.15 (d, C-8), 38.79 (s, C-5), 37.98 (s, C-9), 36.17 (t, C-6), 27.76 (t, C-3), 21.57 (q, C-20), 18.42 (t, C-7), 14.12 (q, C-19); IR (ATR): $\nu_{\max}$ = 2945, 2927, 2853, 1727, 1651, 1436, 1388, 1367, 1252, 1206, 1152, 1071, 1026, 1011, 968, 875, 799, 736 cm^{-1}; ESI-MS (+25 V): *m/z* = 393.3, 395.3 [M+H]$^+$, 410.3, 412.2 [M+NH$_4$]$^+$; elemental analysis calcd (%) for $C_{21}H_{25}ClO_5$: C 64.20, H 6.41; found: C 64.44, H 6.45.

2.21 Dihydroxylation of vinyl bromide *rac*-7 to give *rac*-13

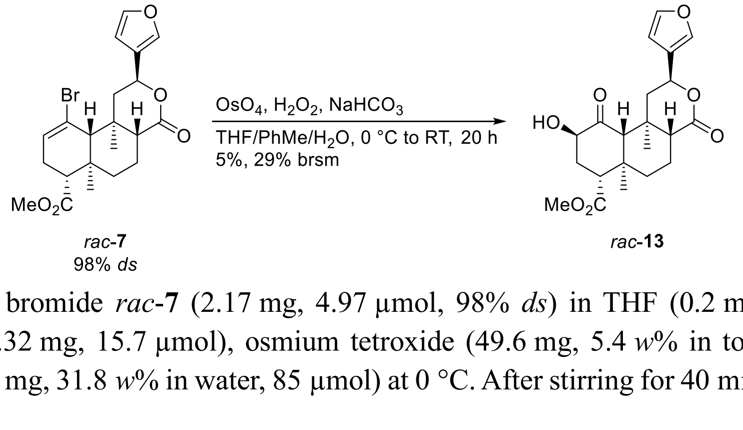

rac-**7** 98% *ds* *rac*-**13**

To a solution of vinyl bromide *rac*-**7** (2.17 mg, 4.97 µmol, 98% *ds*) in THF (0.2 mL) was added sodium hydrogen carbonate (1.32 mg, 15.7 µmol), osmium tetroxide (49.6 mg, 5.4 *w*% in toluene, 10.5 µmol) and hydrogen peroxide (9.1 mg, 31.8 *w*% in water, 85 µmol) at 0 °C. After stirring for 40 min, the reaction mixture

was warmed to room temperature and stirred for 19 h. Subsequently, saturated aqueous Na_2SO_3 and EtOAc were added. The aqueous layer was extracted three times with EtOAc. The combined organic layers were dried over $MgSO_4$ and concentrated under reduced pressure. Flash chromatography (CH_2Cl_2/Et_2O 3:1) of the residue afforded vinyl bromide *rac*-**7** (1.8 mg, 4.1 µmol, 83%) and α-hydroxy ketone *rac*-**13** (0.10 mg, 0.26 µmol, 5%, 29% brsm, analytical data see ref. [7]).

2.22 Dihydroxylation of vinyl chloride *rac*-8 to give *rac*-13

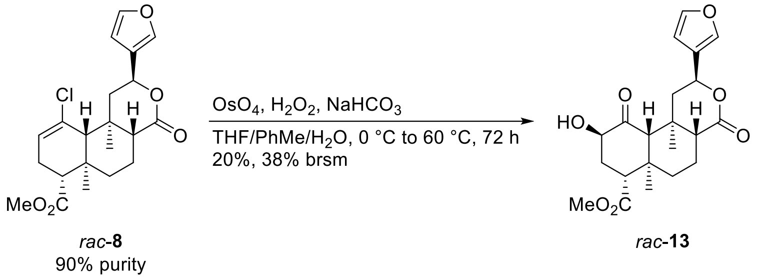

To a solution of vinyl chloride *rac*-**8** (20.9 mg, 53.2 µmol, 90% purity) in THF (2.8 mL) was added sodium hydrogen carbonate (12.4 mg, 148 µmol), osmium tetroxide (0.54 mL, 0.20 M in toluene, 0.10 mmol), and hydrogen peroxide (69.2 mg, 31.8 *w*% in water, 647 µmol) at 0 °C. The reaction mixture was stirred at 60 °C for 72 h. Subsequently, saturated aqueous Na_2SO_3 and EtOAc were added. The aqueous layer was extracted three times with EtOAc. The combined organic layers were dried over $MgSO_4$ and concentrated under reduced pressure. Flash chromatography (CH_2Cl_2/Et_2O 3:1) of the residue afforded vinyl chloride *rac*-**8** (10.1 mg, 25.7 µmol, 48%) and α-hydroxy ketone *rac*-**13** (4.2 mg, 10.8 µmol, 20%, 38% brsm, analytical data see ref. [7])

2.23 Conversion of lactone 4 to diol 14

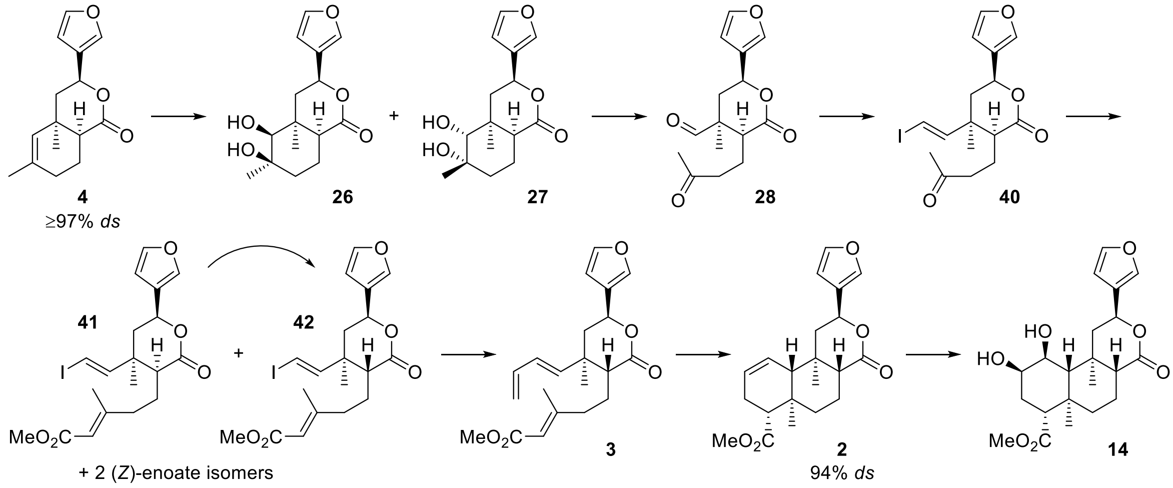

Lactone **4** was converted to diol **14** in 8 steps according to our synthesis in the racemic series.[7] Specific rotations $[\alpha]_D$ are listed below. The remaining analytical data for each compound can be found in ref. [7].

28: colorless wax; $[\alpha]_D^{23}$ = +22.0 (*c* 1.55 in CH_2Cl_2).
40: colorless wax; $[\alpha]_D^{21}$ = +2.6 (*c* 0.94 in CH_2Cl_2).
41: colorless solid; m.p. 84–86 °C; $[\alpha]_D^{24}$ = +29.6 (*c* 0.91 in CH_2Cl_2).
42: colorless wax; $[\alpha]_D^{23}$ = +35.9 (*c* 1.19 in CH_2Cl_2).
3: pale-yellow liquid; $[\alpha]_D^{21}$ = +27.7 (*c* 1.10 in CH_2Cl_2).
2: colorless solid; m.p. 150–152 °C; $[\alpha]_D^{20}$ = +11.3 (*c* 1.01 in CH_2Cl_2).
14: colorless solid; m.p. 216–220 °C; $[\alpha]_D^{20}$ = –39.5 (*c* 0.55 in CH_2Cl_2).

2.24 Chemoselective Mitsunobu inversion of diol 14 to give 15

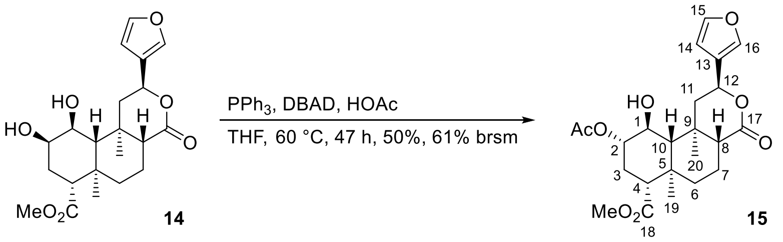

A solution of diol **14** (5.4 mg, 14 µmol), triphenylphosphine (36.8 mg, 140 µmol), di-*tert*-butyl azodicarboxylate (20.1 mg, 87.3 µmol), and acetic acid (20.1 mg, 335 µmol) in THF (1.5 mL) was heated at 60 °C. After 3 h, 6 h, 24 h, and 27 h (total reaction time), the three reagents triphenylphosphine, di-*tert*-butyl azodicarboxylate, and acetic acid were added again in the quantities mentioned above. After a total reaction time of 47 h, the solution was cooled to room temperature, diluted with EtOAc and saturated aqueous $NaHCO_3$. The aqueous layer was extracted three times with EtOAc. The organic layers were combined, dried over $MgSO_4$, and concentrated under vacuum. Purification by flash chromatography (CH_2Cl_2/Et_2O 8:1 to 4:1 to 1:1) afforded pure alcohol **15** (3.0 mg, 6.9 µmol, 50%, 61% brsm) as a colorless solid and a mixture of unreacted diol **14** (0.97 mg, 2.5 µmol, 18%) and triphenylphosphine oxide. Pure starting material **14** can be obtained by purification via flash chromatography (isohexane/EtOAc 1:2).

15: R_f = 0.30 (CH_2Cl_2/Et_2O 8:1); m.p. 77–80 °C; $[\alpha]_D^{21}$ = +3.4 (*c* 0.94 in $CDCl_3$); ^{1}H NMR (600 MHz, $CDCl_3$): δ = 7.43–7.40 (m, 1 H, H-16), 7.40–7.37 (m, 1 H, H-15), 6.46–6.34 (m, 1 H, H-14), 5.44 (dd, *J* = 11.1, 5.8 Hz, 1 H, H-12), 4.66 (ddd, *J* = 11.5, 9.0, 5.8 Hz, 1 H, H-2), 4.06 (t, *J* = 10.0 Hz, 1 H, H-1), 3.65 (s, 3 H, OC***H***3), 3.40 (dd, *J* = 14.1, 5.8 Hz, 1 H, H-11α), 2.27 (dd, *J* = 13.6, 3.4 Hz, 1 H, H-4), 2.23 (br. s, 1 H, O***H***), 2.21 (dd, *J* = 12.4, 3.4 Hz, 1 H, H-8), 2.10 (s, 3 H, C***H***3C=O), 2.13–2.03 (m, 2 H, H-3β, H-7β), 1.95 (td, *J* = 13.4, 11.7 Hz, 1 H, H-3α), 1.76 (dd, *J* = 14.1, 11.1 Hz, 1 H, H-11β), 1.67–1.57 (m, 2 H, H-6α, H-7α), 1.37–1.30 (m, 1 H, H-6β), 1.25 (s, 3 H, H-20), 1.22 (d, *J* = 10.9 Hz, 1 H, H-10), 1.16 (s, 3 H, H-19); ^{13}C NMR (151 MHz, $CDCl_3$): δ = 172.66 (s, C-18), 172.47 (s, C-17), 171.34 (s, ***C***OCH3), 143.78 (d, C-15), 139.39 (d, C-16), 126.28 (s, C-13), 108.74 (d, C-14), 78.20 (d, C-2), 71.96 (d, C-1), 71.78 (d, C-12), 56.40 (d, C-10), 53.56 (d, C-4), 51.99 (d, C-8), 51.75 (q, O***C***H3), 46.34 (t, C-11), 38.72 (t, C-6), 38.31 (s, C-5), 37.63 (s, C-9), 28.68 (t, C-3), 21.30 (q, CO***C***H3), 18.40 (t, C-7), 16.24 (q, C-19), 15.01 (q, C-20); IR (ATR): ν_{max} = 3497, 2922, 2852, 1724, 1708, 1686, 1652, 1437, 1376, 1279, 1233, 1197, 1160, 1083, 1023, 991, 950, 893, 873, 784, 732, 695, 650 cm^{-1}; ESI-MS (+10 V): *m/z* = 435.7 $[M+H]^+$, 886.7 $[2M+NH_4]^+$; HRMS (ESI): *m/z* calcd for $C_{23}H_{30}O_8+H^+$: 435.2013 $[M+H]^+$; found: 435.2020.

2.25 Oxidation of alcohol 15 to give (–)-salvinorin A (1)

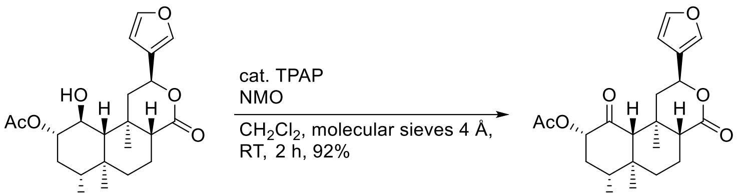

Alcohol **15** (7.0 mg, 16 µmol) was treated with molecular sieves 4 Å (80 mg). In a second flask, $MgSO_4$ (12.2 mg) was added to a solution of *N*-methylmorpholine-*N*-oxide monohydrate (13.5 mg, 99.9 µmol) in CH_2Cl_2 (0.9 mL), and the mixture was stirred for 1 h at room temperature. Subsequently, this NMO solution was transferred to the first flask with filtration. The resultant mixture was stirred for 45 min, treated with tetrapropylammonium perruthenate (1 crystal), and stirring was continued for 2 h at room temperature. Then the mixture was filtered through silica gel (elution with CH_2Cl_2/Et_2O 1:1), and concentrated under vacuum. Purification by flash chromatography (CH_2Cl_2/Et_2O 6:1) gave (–)-salvinorin A (**1**; 6.4 mg, 15 µmol, 92%) as a colorless solid.

1: R_f = 0.58 (CH_2Cl_2/Et_2O 6:1); m.p. 236–238 °C (Lit.:[9,10] 238–240 °C, 242–244 °C); $[\alpha]_D^{21}$ = –42.3 (*c* 0.74 in $CHCl_3$) (Lit.:[9,10] –41, –45.3); ^1H NMR (600 MHz, $CDCl_3$): δ = 7.42–7.40 (m, 1 H), 7.39 (t, *J* = 1.7 Hz, 1 H), 6.41–6.33 (m, 1 H), 5.53 (dd, *J* = 11.7, 5.3 Hz, 1 H), 5.19–5.11 (m, 1 H), 3.73 (s, 3 H), 2.78–2.71 (m, 1 H), 2.51 (dd, *J* = 13.6, 5.3 Hz, 1 H), 2.34–2.26 (m, 2 H), 2.20–2.13 (m, 2 H), 2.17 (s, 3 H), 2.07 (dd, *J* = 11.9, 2.8 Hz, 1 H), 1.80 (dt, *J* = 13.2, 3.0 Hz, 1 H), 1.70–1.61 (m, 1 H), 1.61–1.54 (m, 2 H), 1.45 (s, 3 H), 1.12 (s, 3 H); ^{13}C NMR (151 MHz, $CDCl_3$): δ = 202.14 (s), 171.68 (s), 171.24 (s), 170.11 (s), 143.87 (d), 139.57 (d), 125.39 (s), 108.53 (d), 75.18 (d), 72.19 (d), 64.28 (d), 53.77 (d), 52.12 (q), 51.58 (d), 43.59 (t), 42.25 (s), 38.33 (t), 35.62 (s), 30.93 (t), 20.72 (q), 18.30 (t), 16.55 (q), 15.36 (q); IR (ATR): ν_{max} = 3128, 2952, 2926, 2853, 1724, 1652, 1447, 1396, 1374, 1274, 1231, 1198, 1155, 1107, 1045, 1020, 996, 949, 891, 872, 825, 880, 741, 687 cm^{-1}; ESI-MS (+10 V): *m/z* = 433.3 $[M+H]^+$, 450.3 $[M+NH_4]^+$; HRMS (ESI): *m/z* calcd for $C_{46}H_{56}O_{16}+Na^+$: 887.3461 $[2M+Na]^+$; found: 887.3465.

Table S1: Comparison of ^{13}C NMR Data (values in ppm, solvent $CDCl_3$) for **1** with Literature Data: ref. [9] 51 MHz, ref. [10] 91 MHz.

This work	Ref. [9]	Ref. [10]	This work	Ref. [9]	Ref. [10]
202.14	202.04	202.02	52.12	51.90	51.85
171.68	171.57	171.57	51.58	51.26	51.36
171.24	171.15	171.1	43.59	43.23	43.41
170.11	169.94	169.86	42.25	42.06	42.15
143.87	143.66	143.69	38.33	38.08	38.22
139.57	139.46	139.51	35.62	35.41	35.54
125.39	125.25	125.48	30.93	30.75	30.92
108.53	108.41	108.49	20.72	20.56	20.53
75.18	75.03	75.12	18.30	18.11	18.26
72.19	72.00	72.03	16.55	16.36	16.41
64.28	63.90	64.05	15.36	15.19	15.25
53.77	53.47	53.63			

3 References

[1] T. Carofiglio, D. Marton, G. Tagliavini, *Organometallics* **1992**, *11*, 2961–2963.
[2] S. Guo, M. H. Lim, H. V. Huynh, *Organometallics* **2013**, *32*, 7225–7233.
[3] P. Tang, W. Wang, T. Ritter, *J. Am. Chem. Soc.* **2011**, *133*, 11482–11484.
[4] S. E. Denmark, T. Wynn, *J. Am. Chem. Soc.* **2001**, *123*, 6199–6200.
[5] D. Seyferth, F. G. A. Stone, *J. Am. Chem. Soc.* **1957**, *79*, 515–517.
[6] Y. Wang, V. Rogachev, M. Wolter, M. Gruner, A. Jäger, P. Metz, *Eur. J. Org. Chem.* **2014**, 4083–4088.
[7] Y. Wang, P. Metz, *Org. Lett.* **2018**, 20, 3418–3421.
[8] K. Maruoka, H. Hanawa, T. Hashimoto, *J. Am. Chem. Soc.* **2003**, *125*, 1708–1709.
[9] A. Ortega, J. F. Blount, P. S. Manchand, *J. Chem. Soc. Perkin Trans. 1* **1982**, 2505–2508.
[10] L. J. Valdes, W. M. Butler, G. M. Hatfield, A. G. Paul, M. Koreeda, *J. Org. Chem.* **1984**, *49*, 4716–4720.

4 NMR spectra

4.1 Compound 18

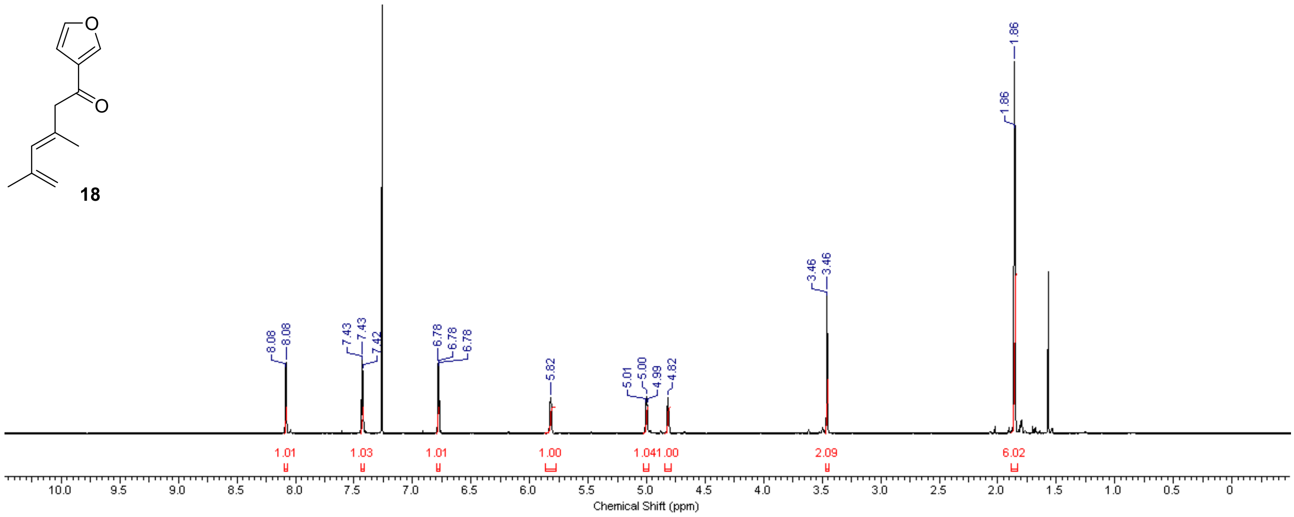

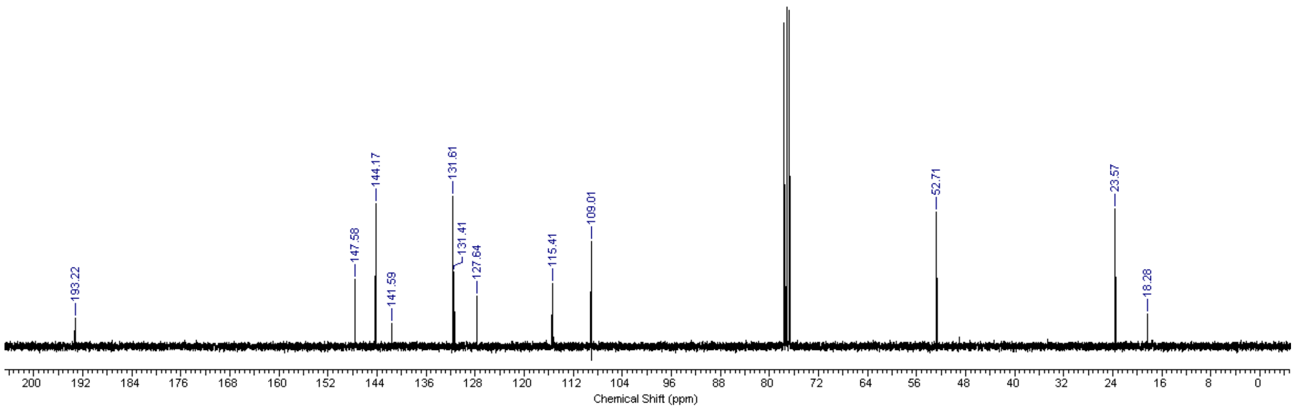

4.2 Compound 17

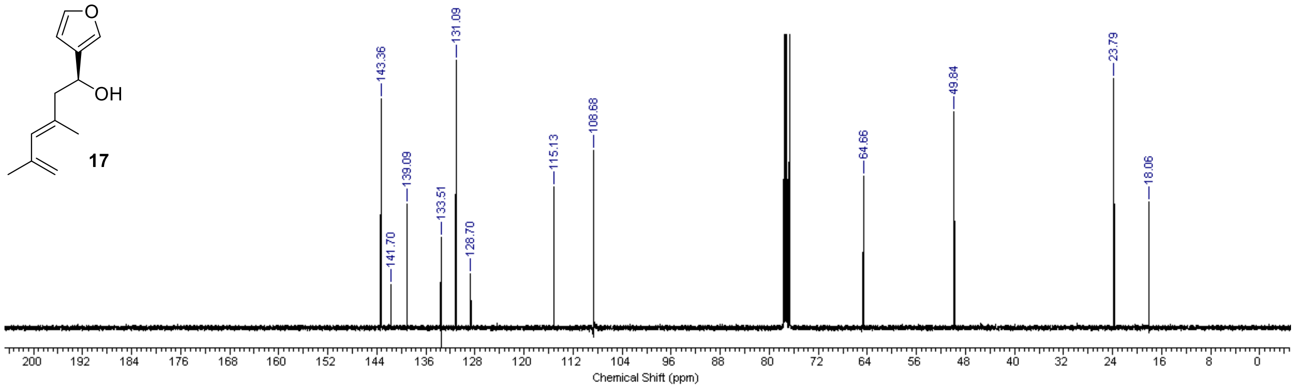

4.3 Compound *rac*-25

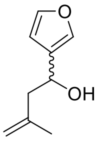

rac-**25**

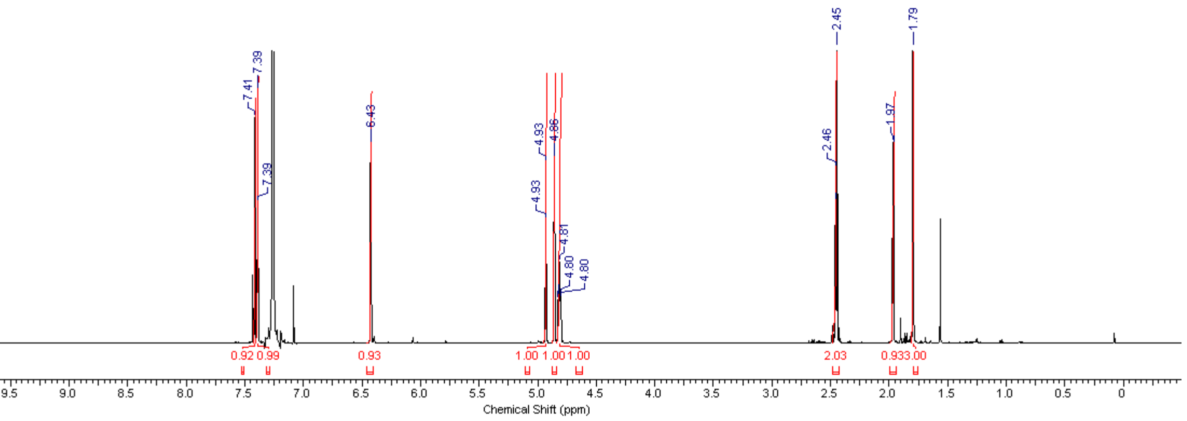

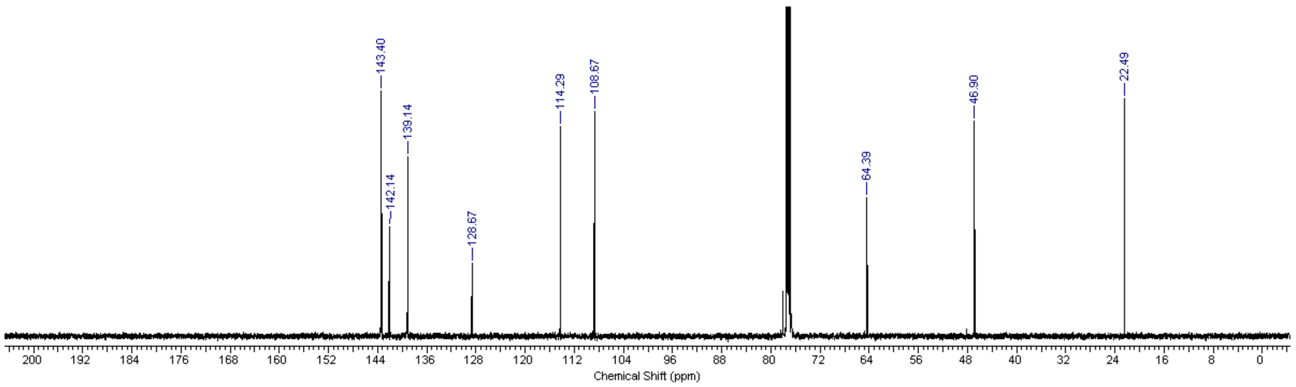

4.4 Compound 5

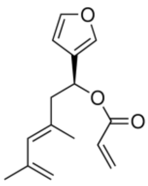

5

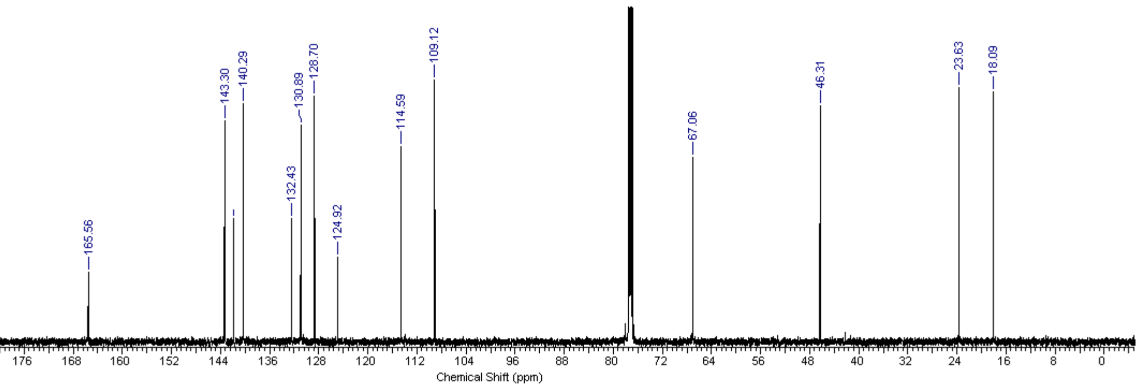

4.5 Compound *rac*-29

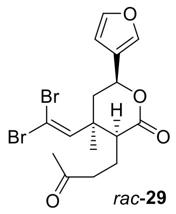

rac-**29**

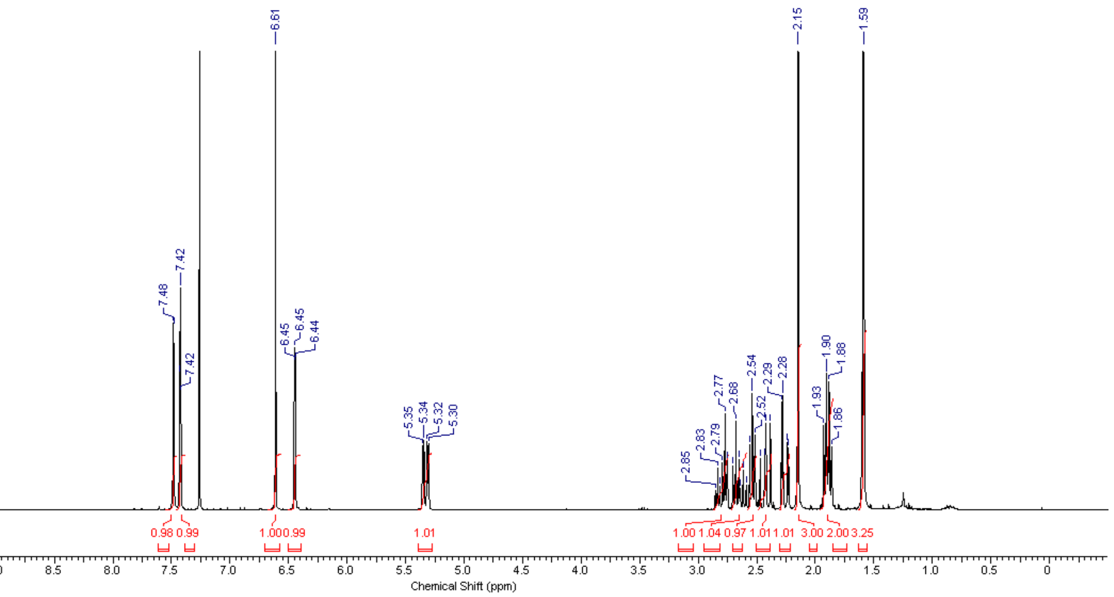

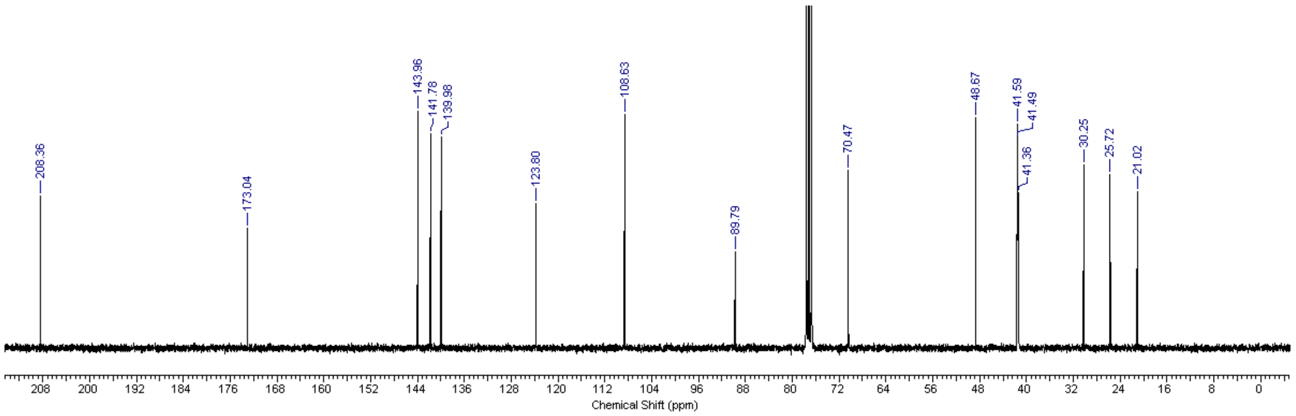

4.6 Compound *rac*-30

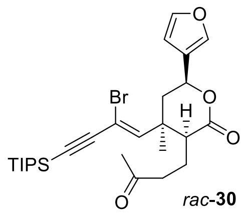

rac-**30**

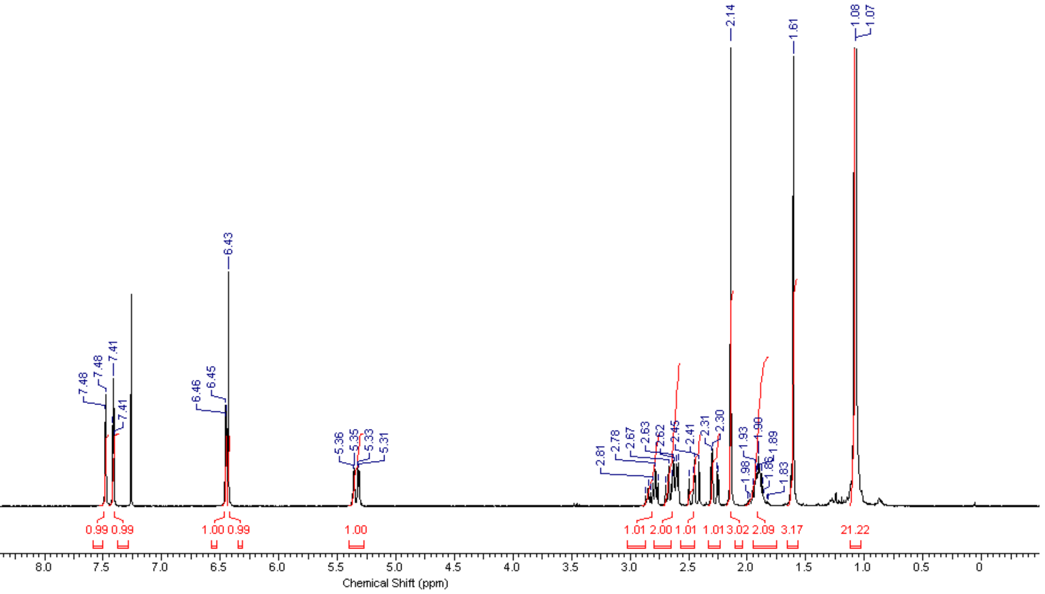

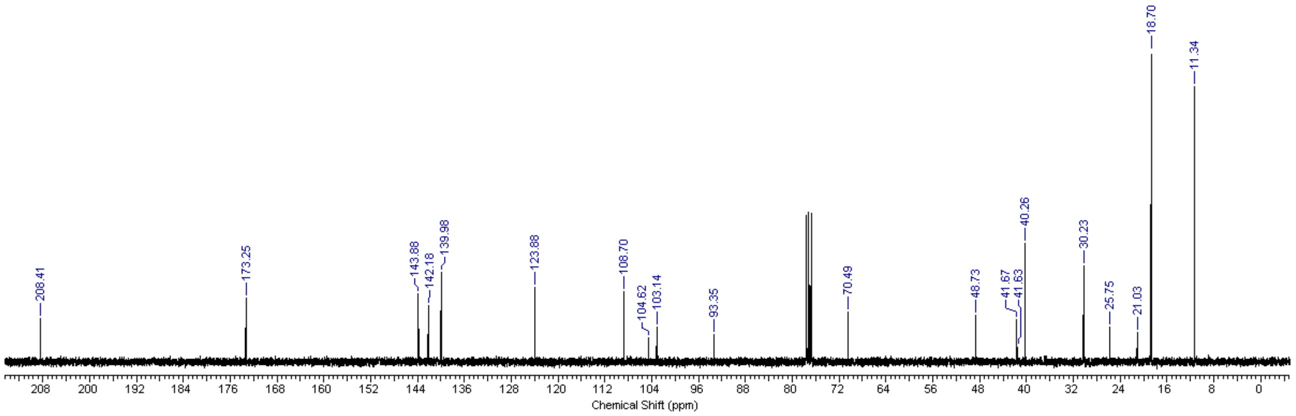

4.7 Compound *rac*-31

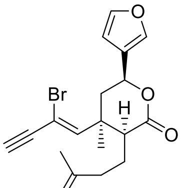

rac-**31**

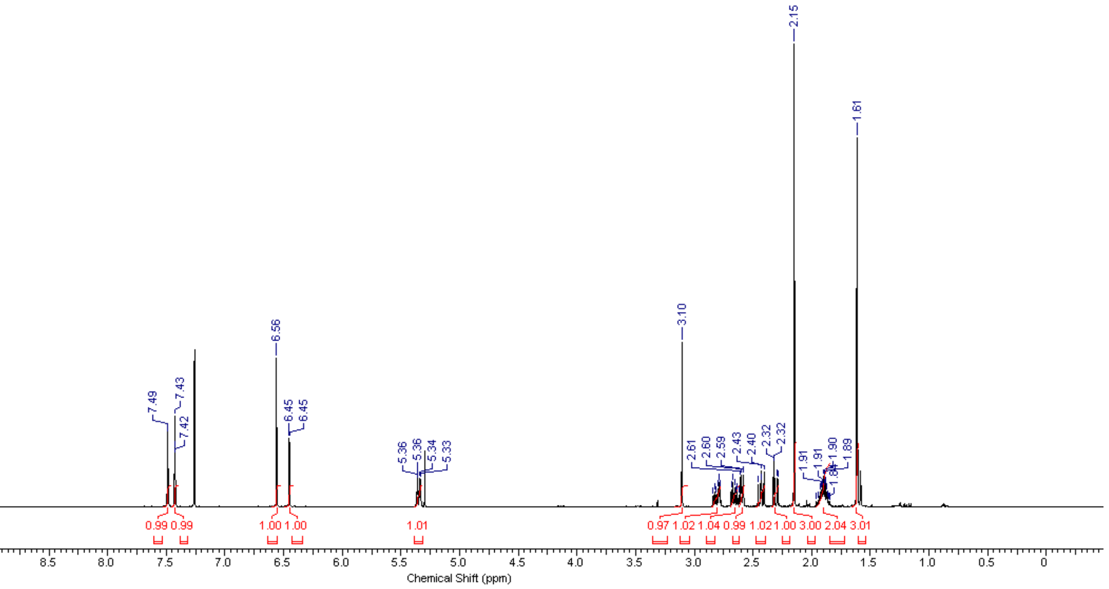

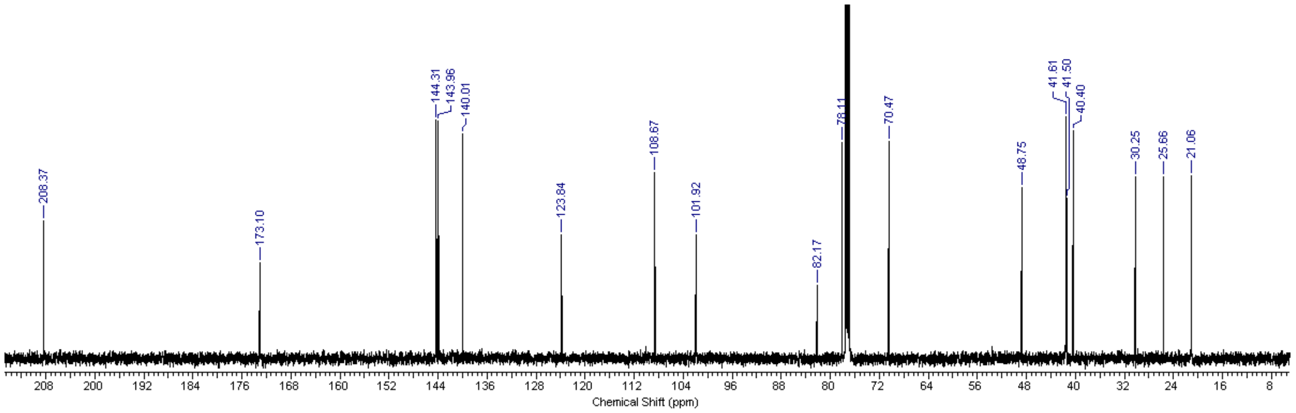

4.8 Compound *rac*-32

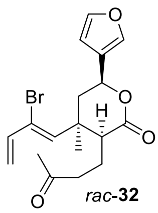

rac-**32**

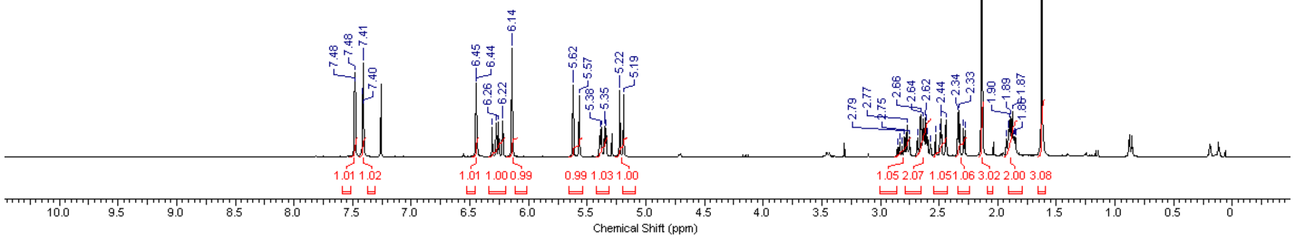

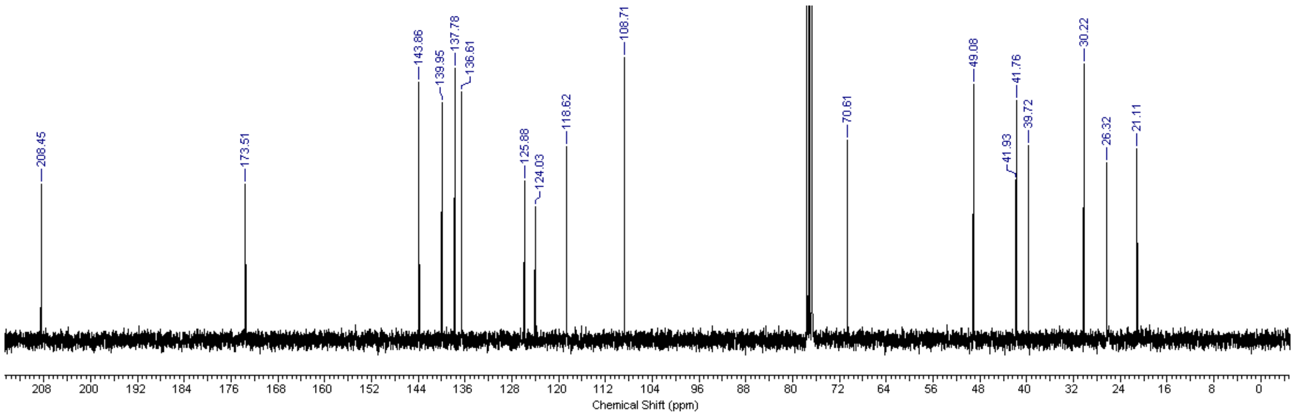

4.9 Compound *rac*-SI-6

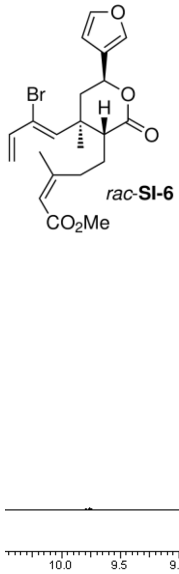

rac-**SI-6**

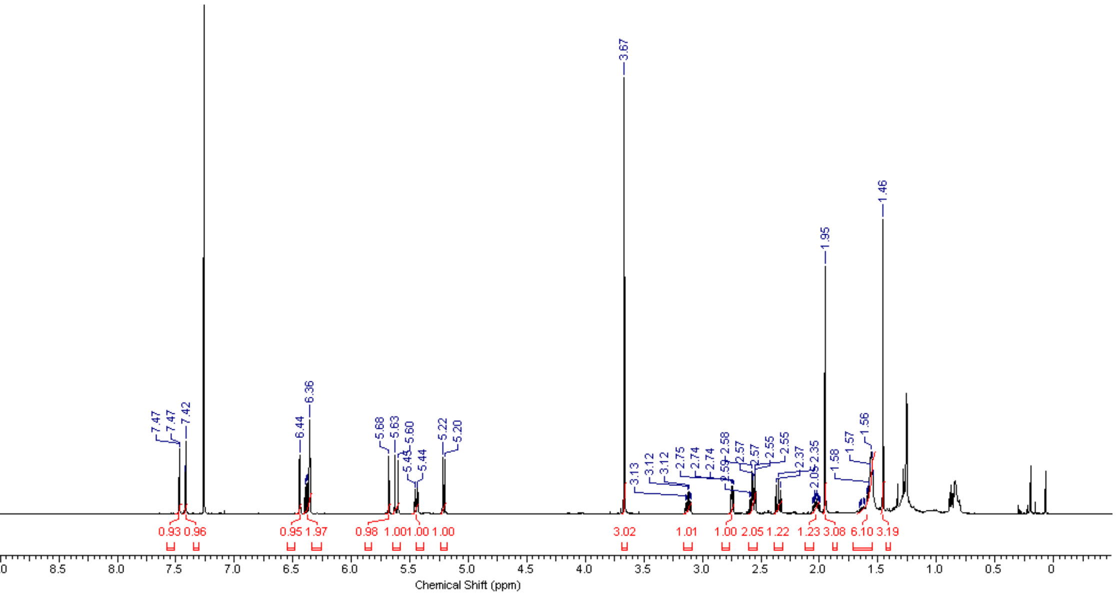

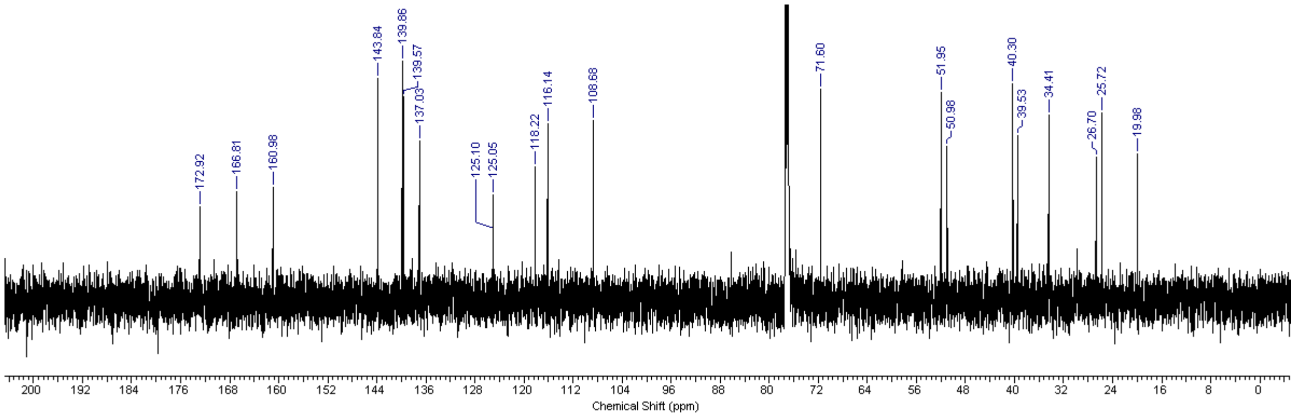

4.10 Compound *rac*-SI-7

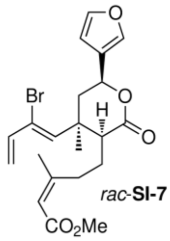

rac-**SI-7**

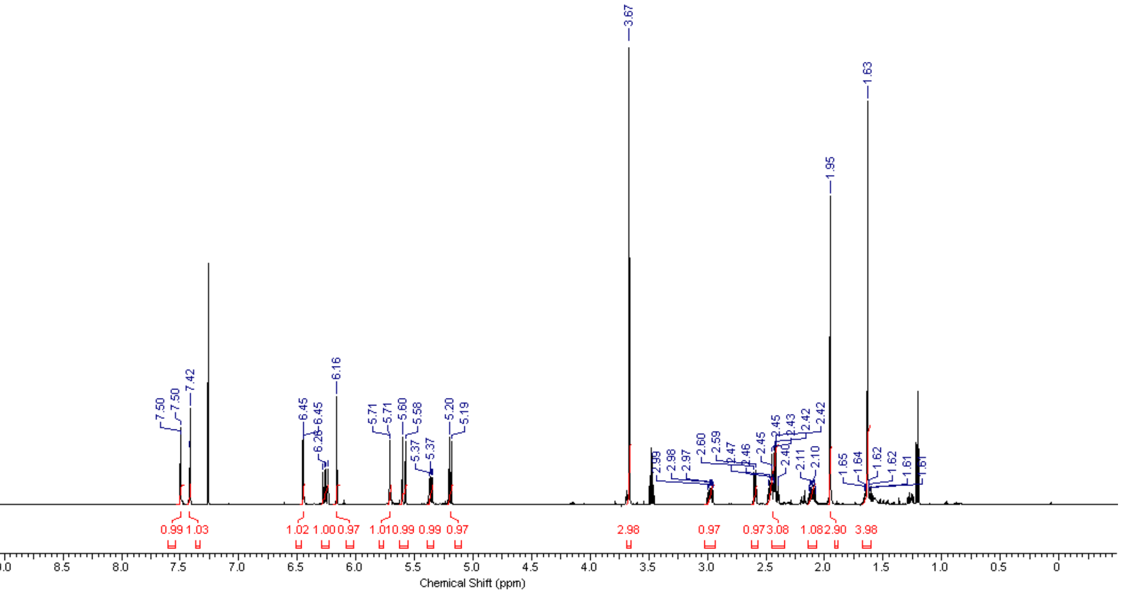

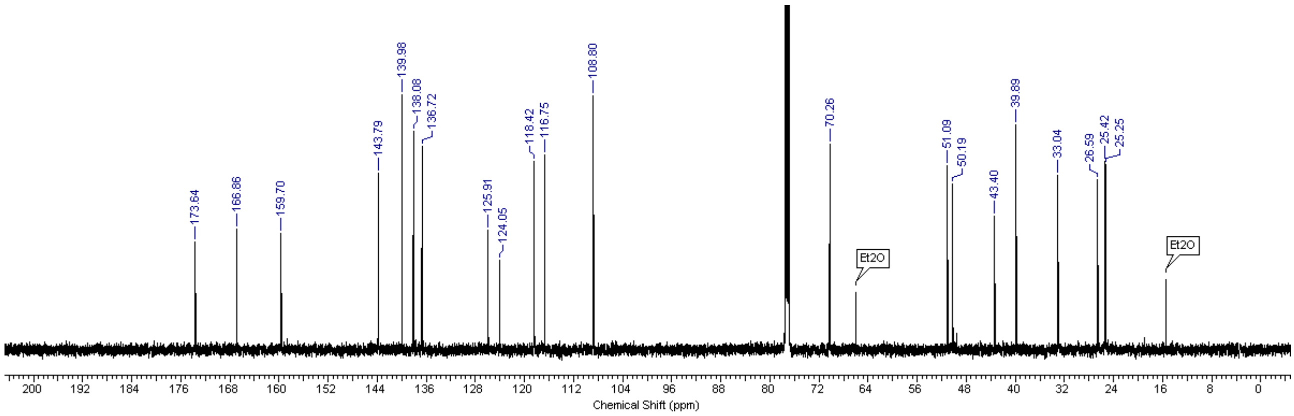

4.11 Compound *rac*-33

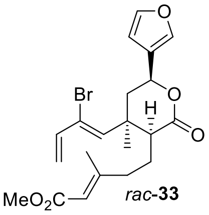

rac-**33**

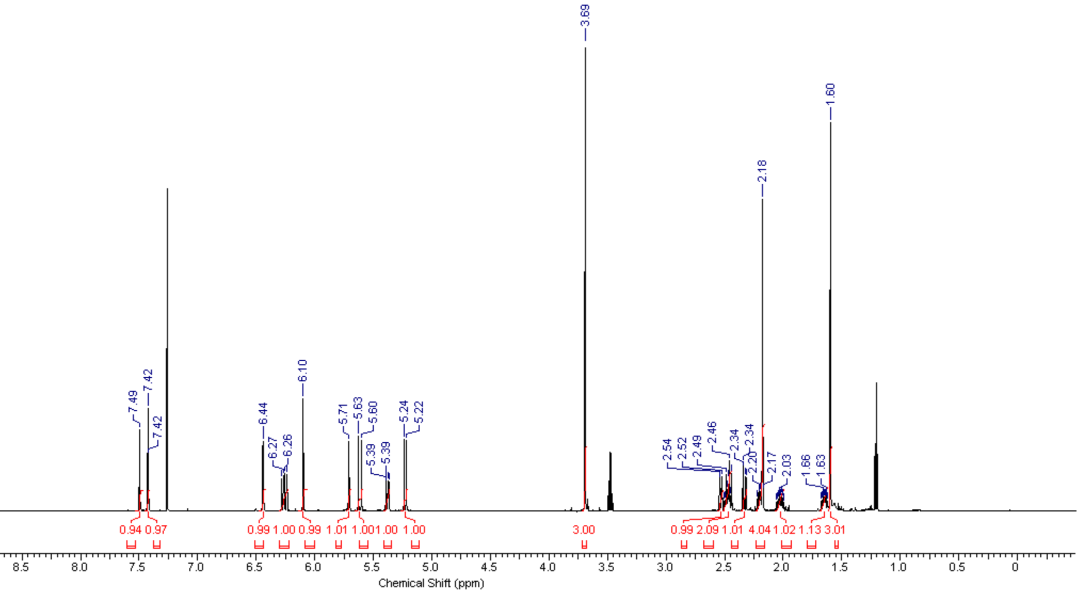

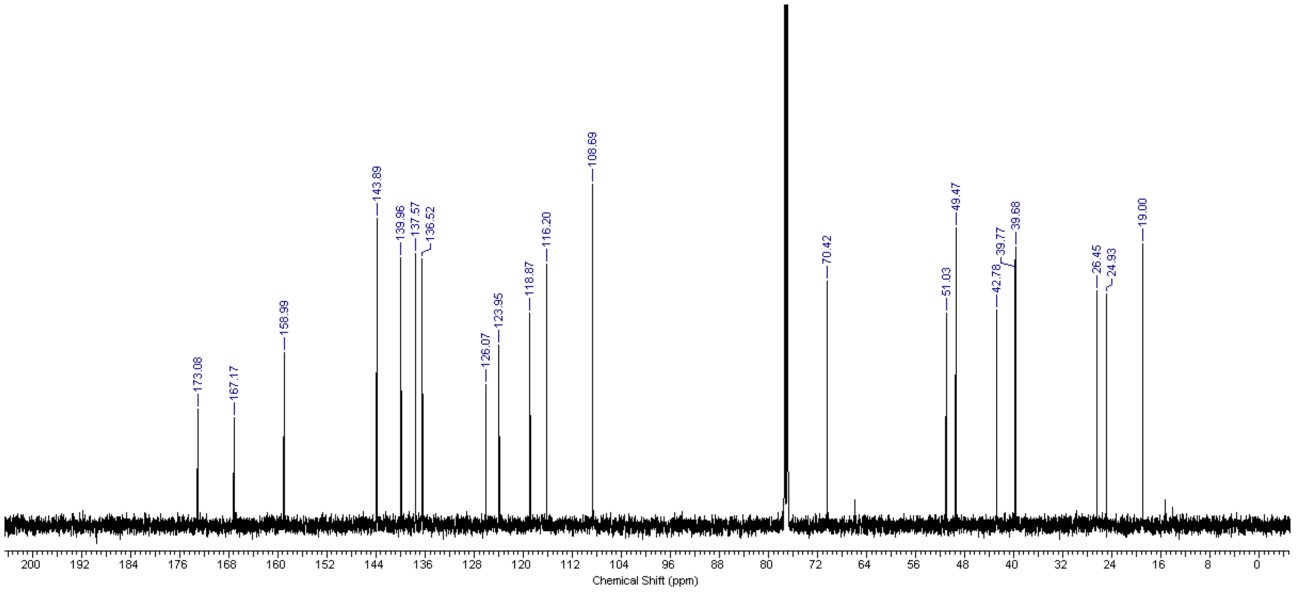

4.12 Compound *rac*-34

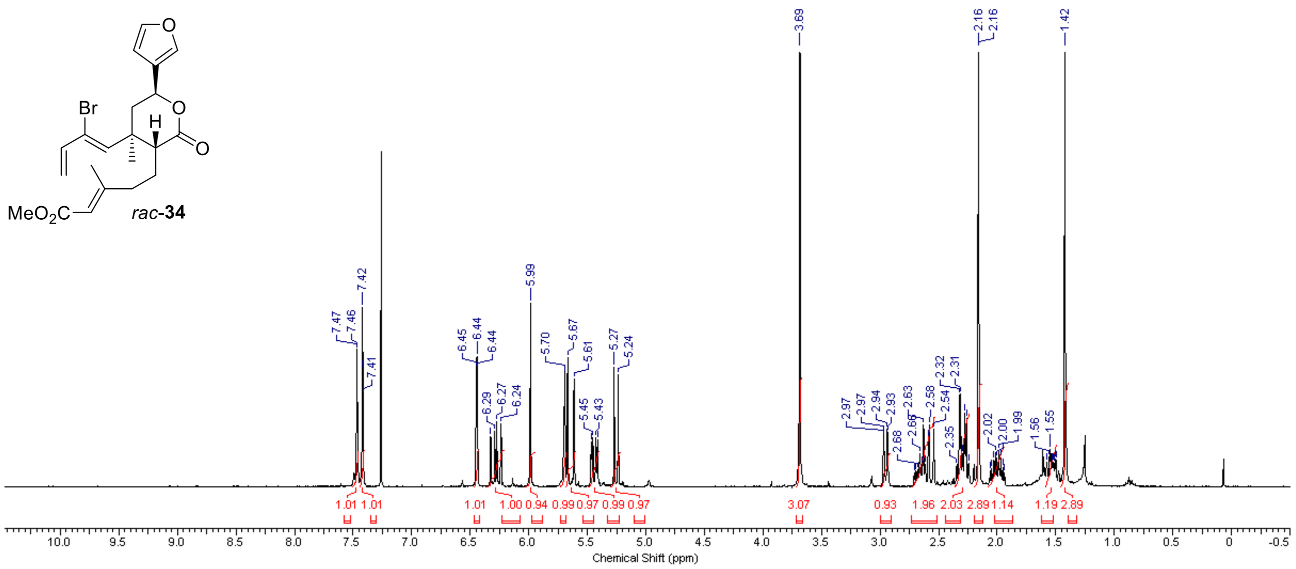

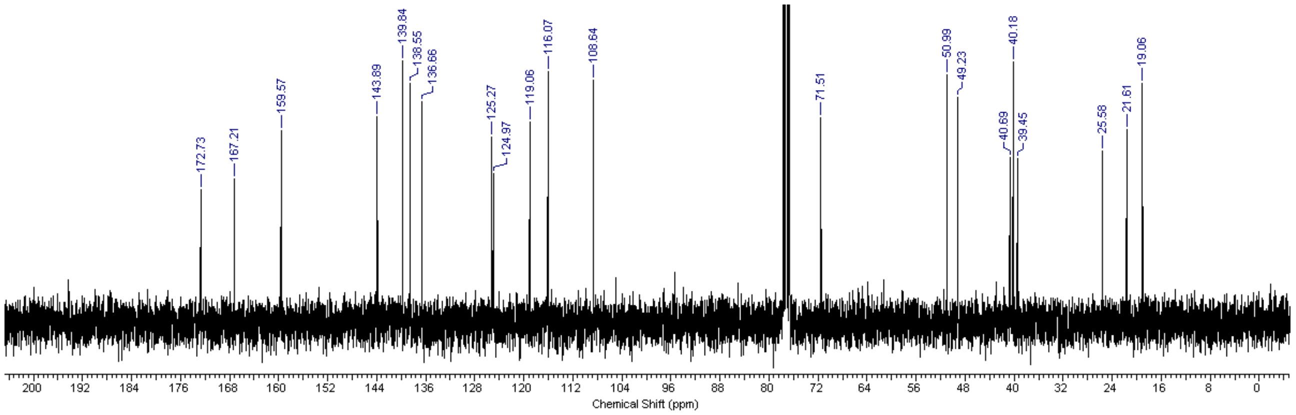

4.13 Compound *rac*-SI-8

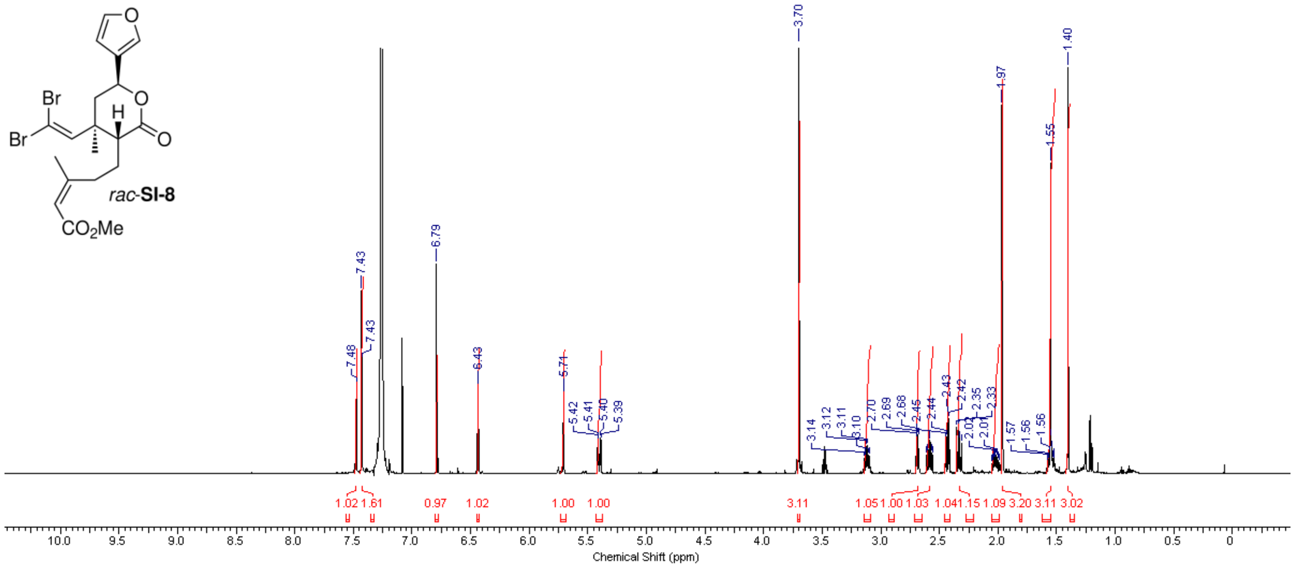

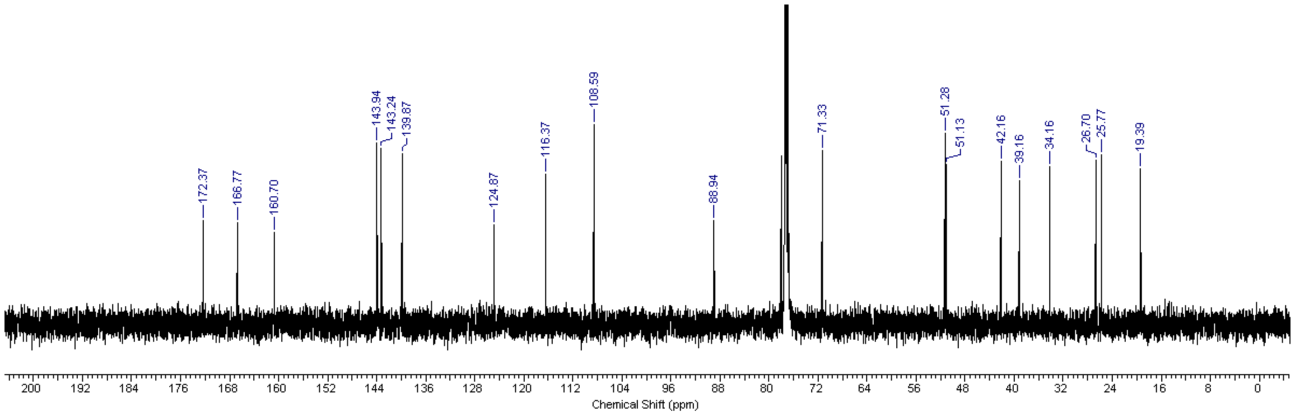

4.14 Compound *rac*-SI-9

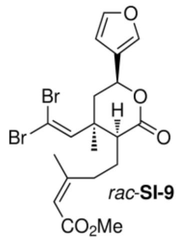

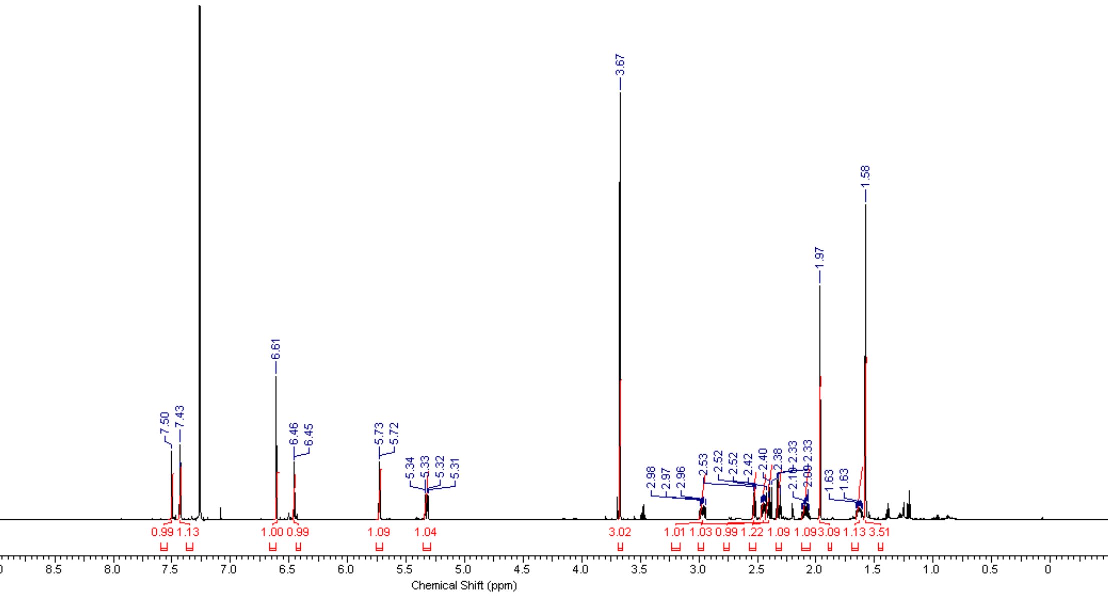

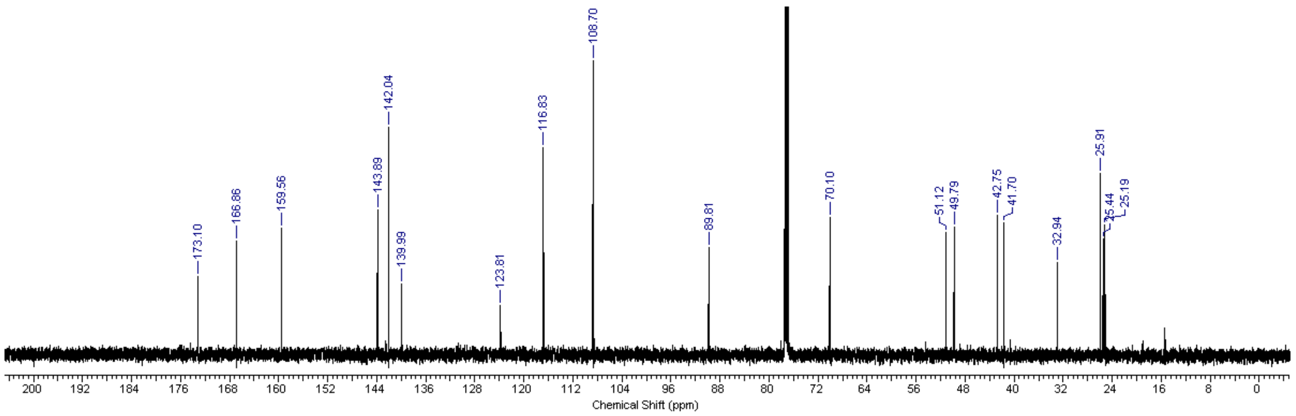

4.15 Compound *rac*-36

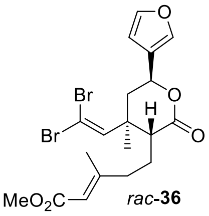

rac-**36**

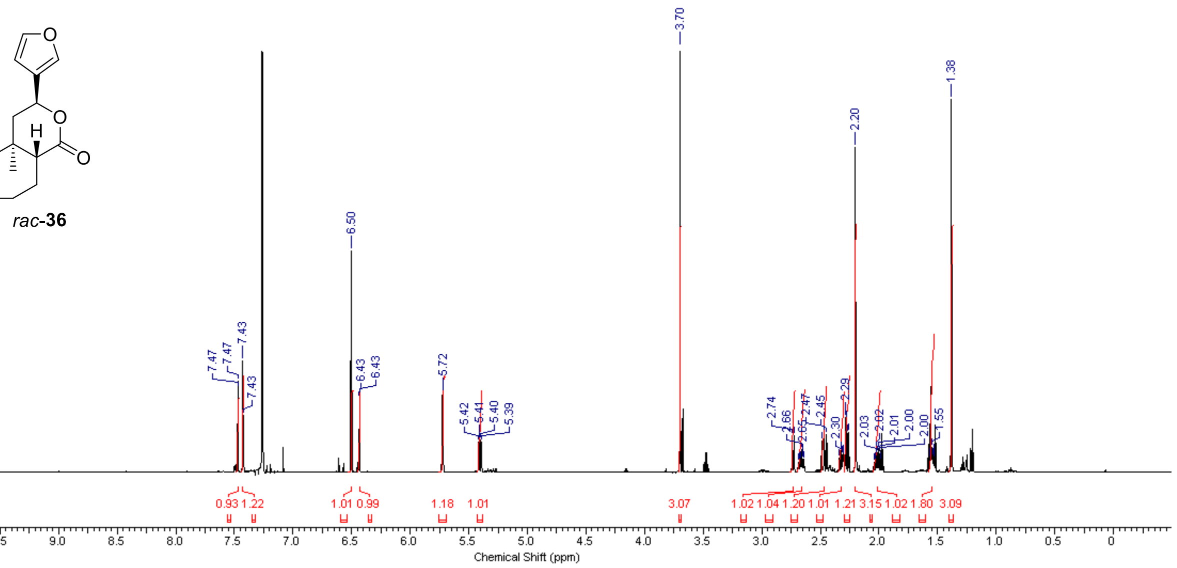

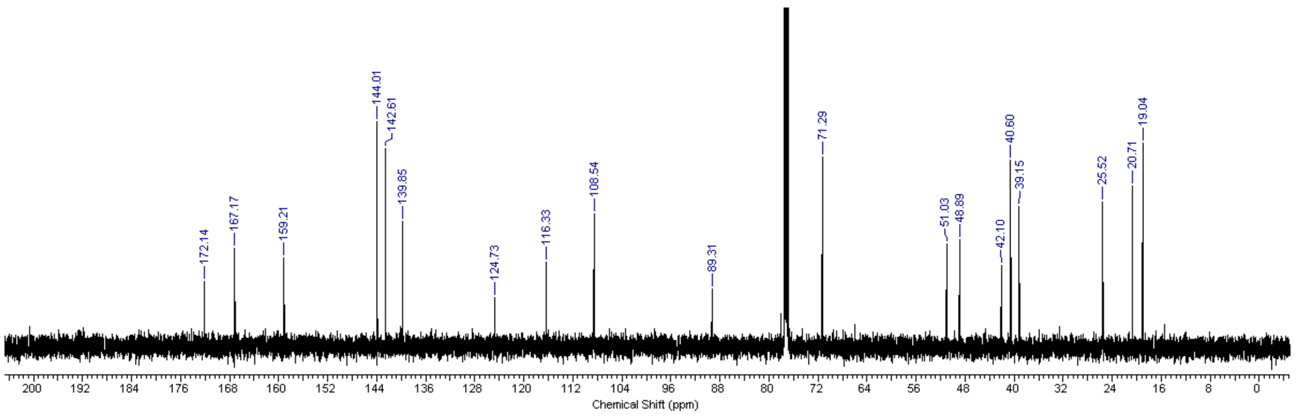

4.16 Compound *rac*-35

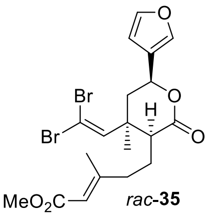

rac-**35**

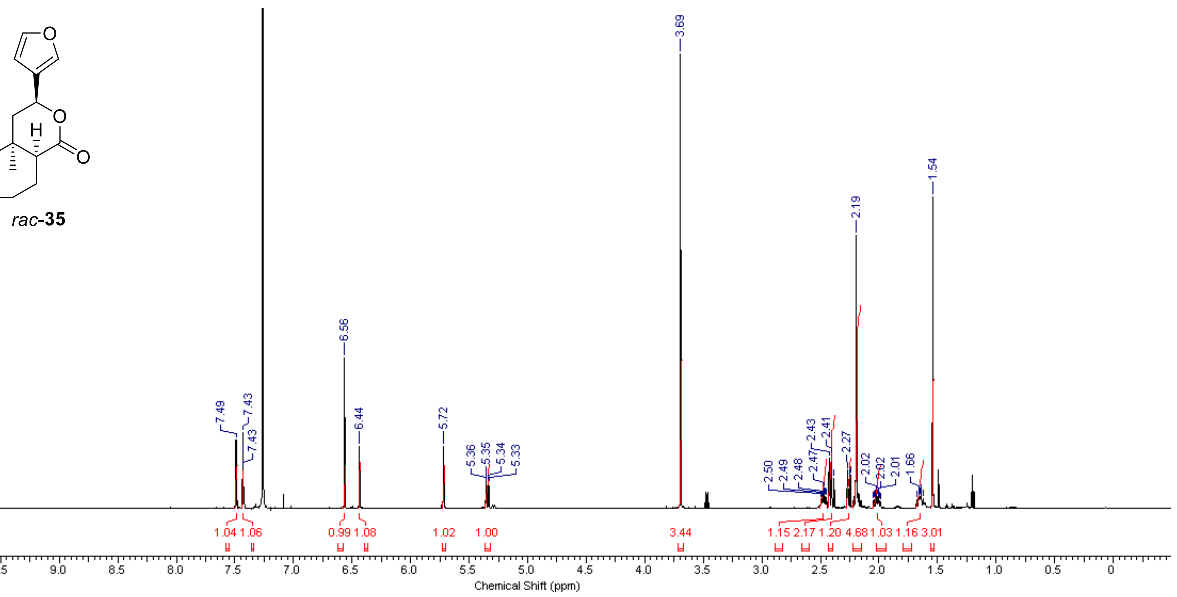

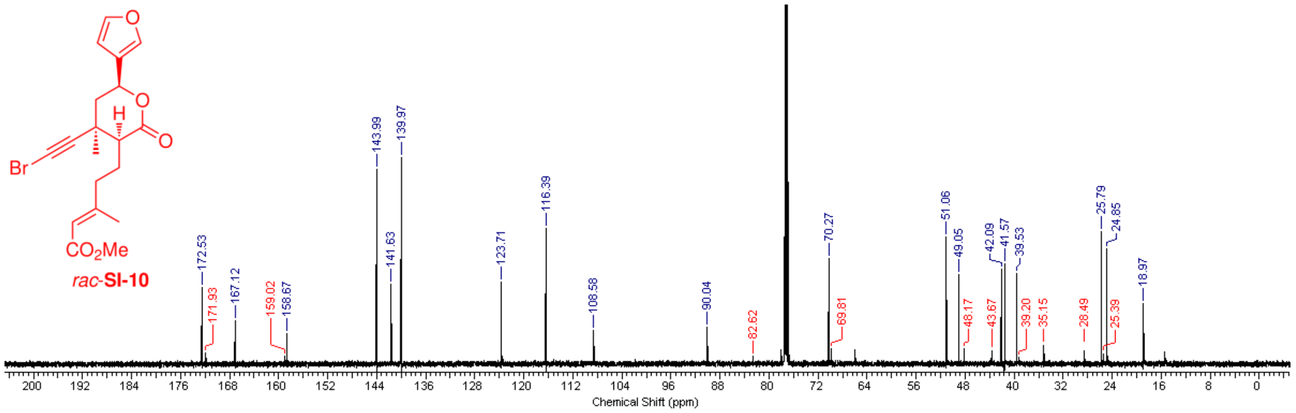

4.17 Compound *rac*-SI-11

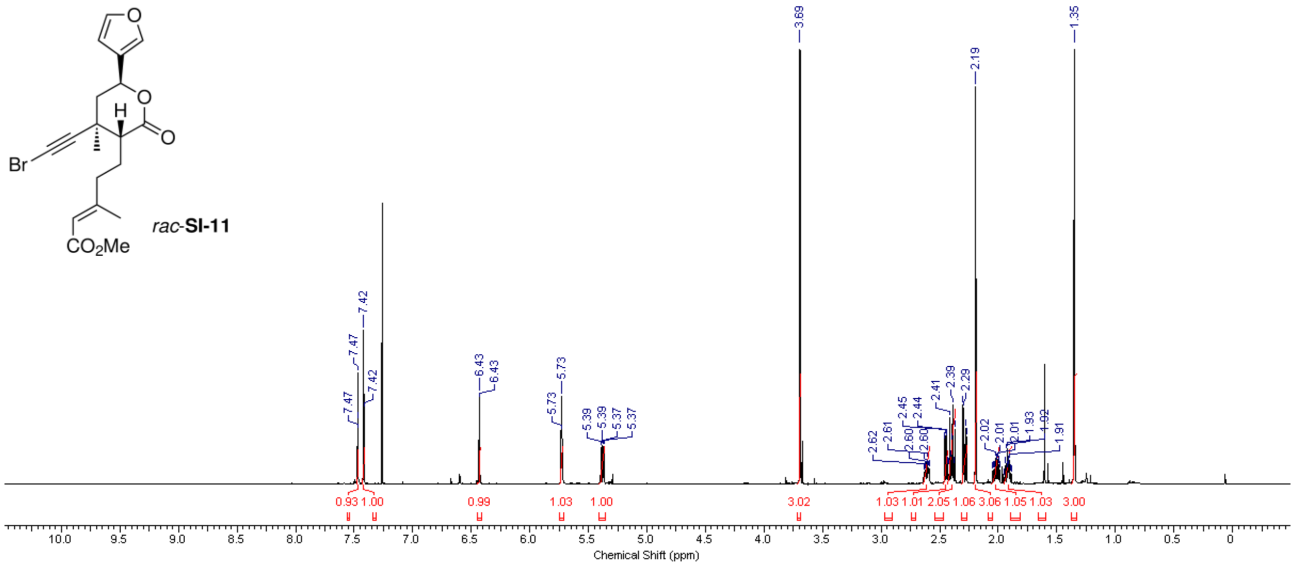

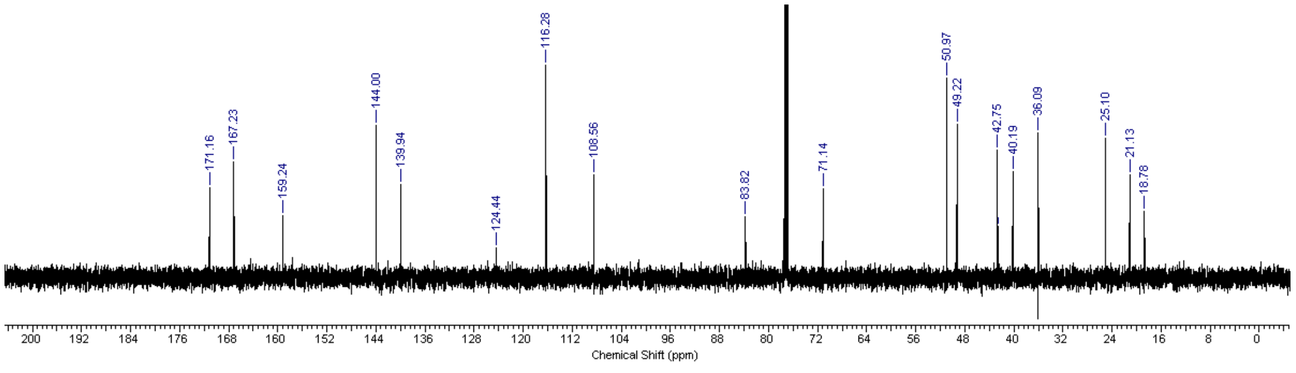

4.18 Compound *rac*-SI-10

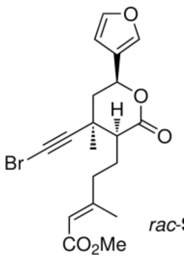

rac-**SI-10**

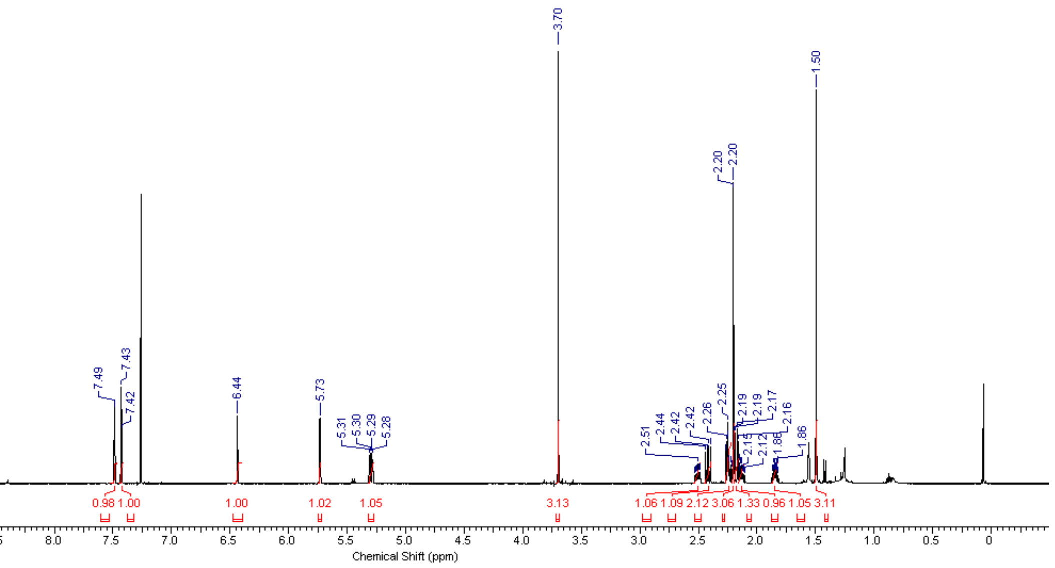

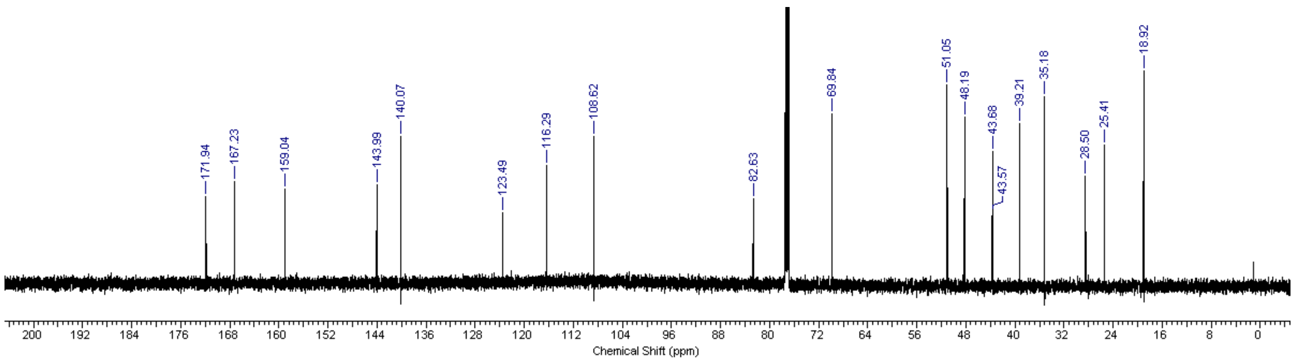

4.19 Compound *rac*-37

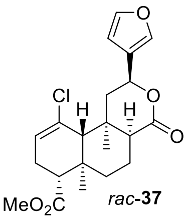

rac-**37**

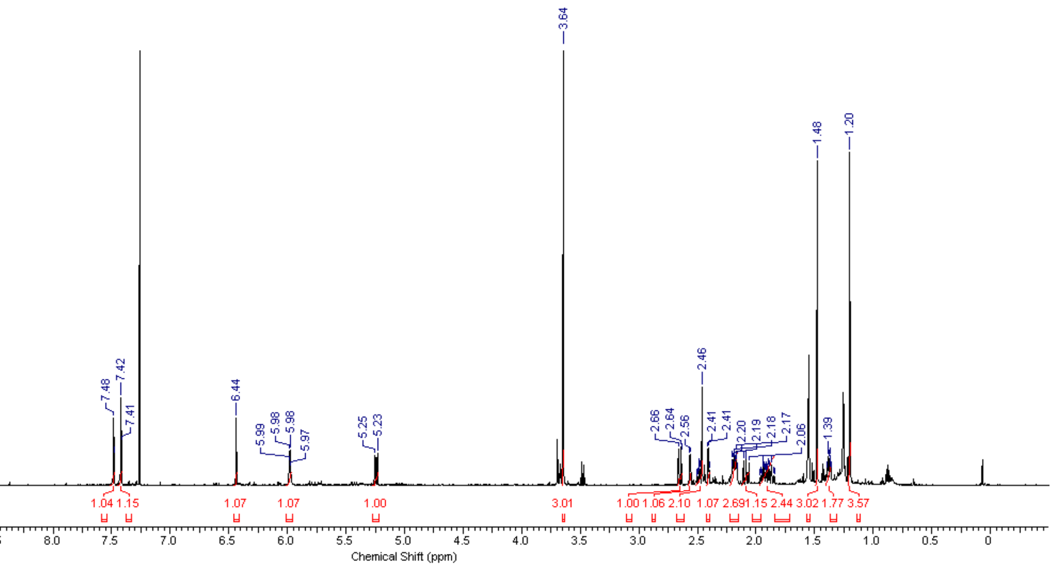

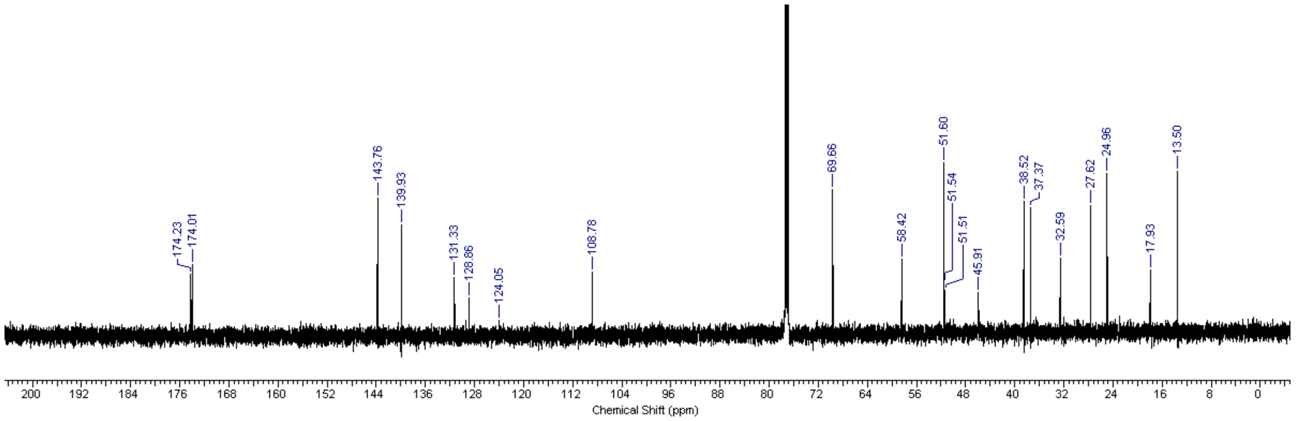

4.20 Compound *rac*-38

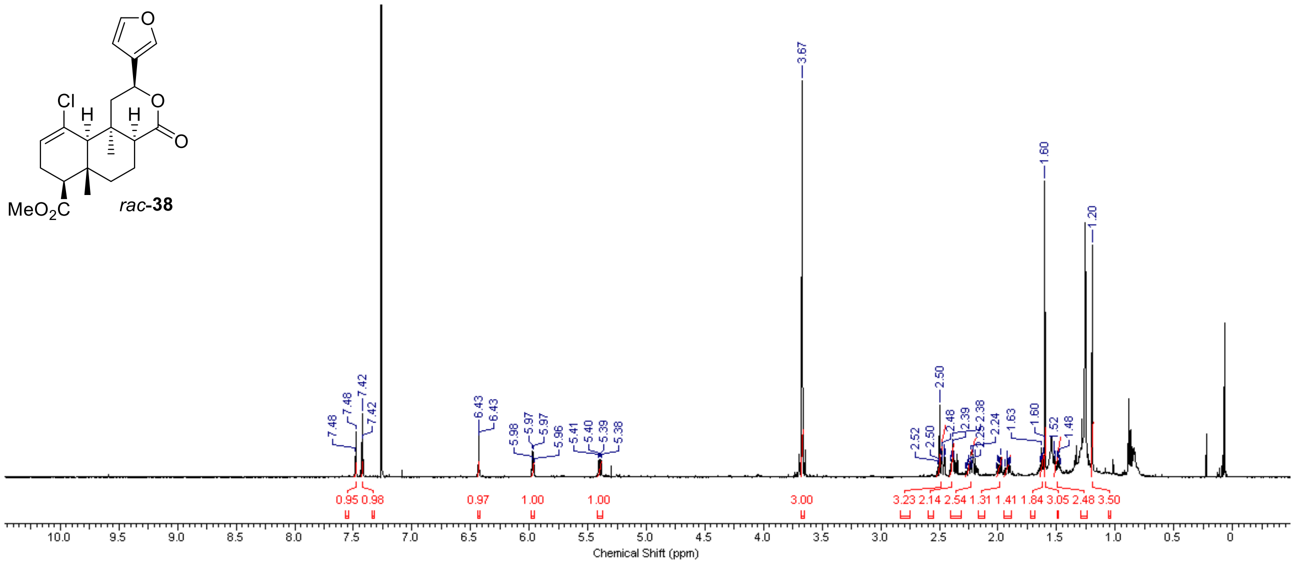

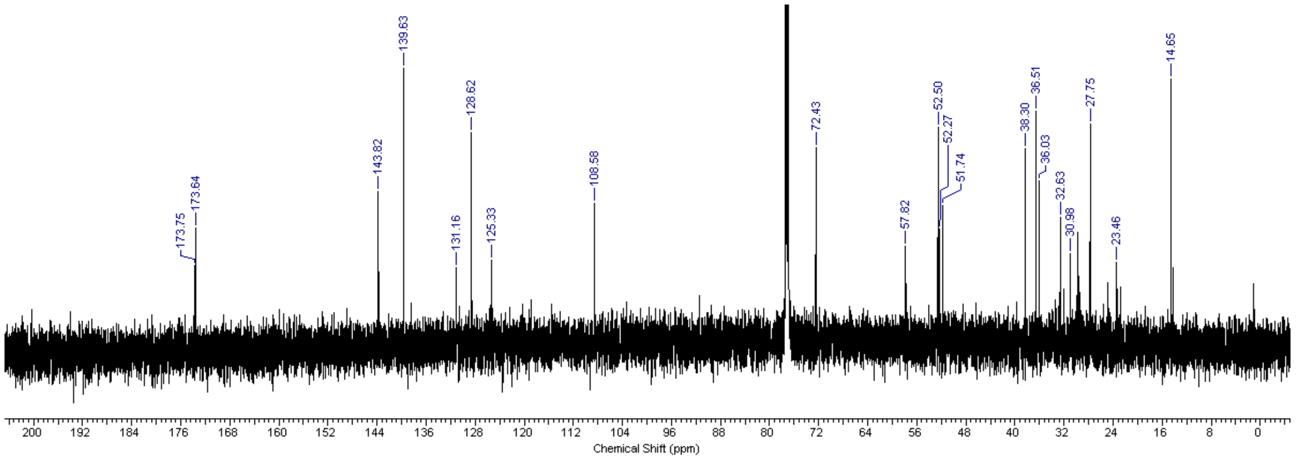

4.21 Compound *rac*-7

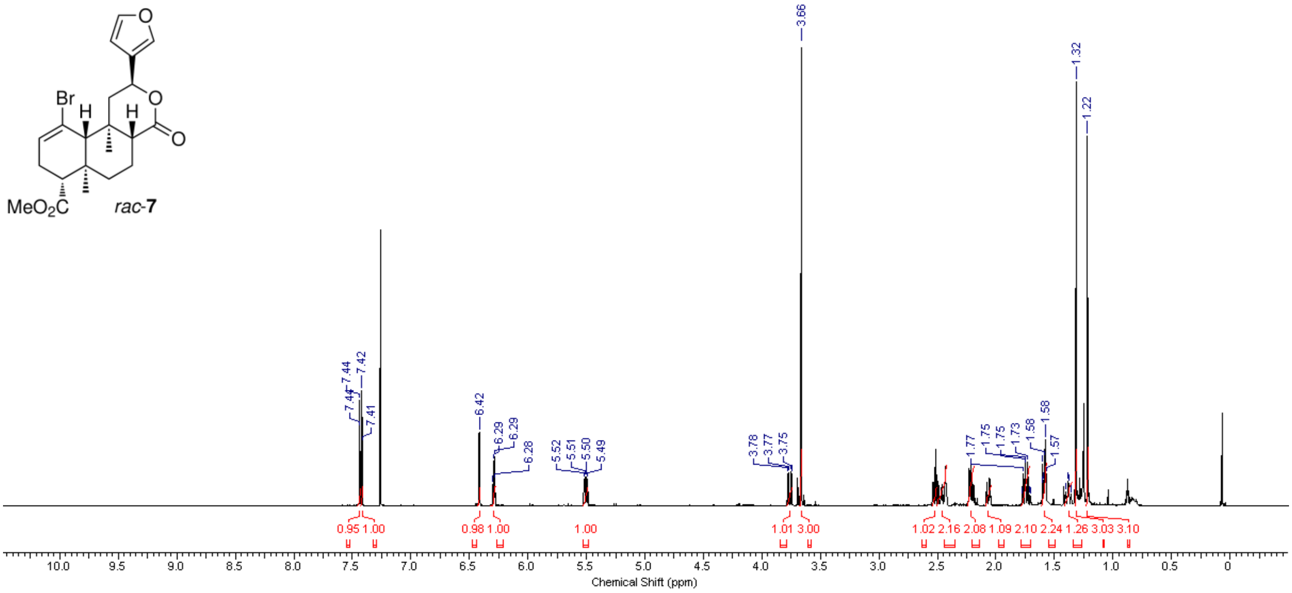

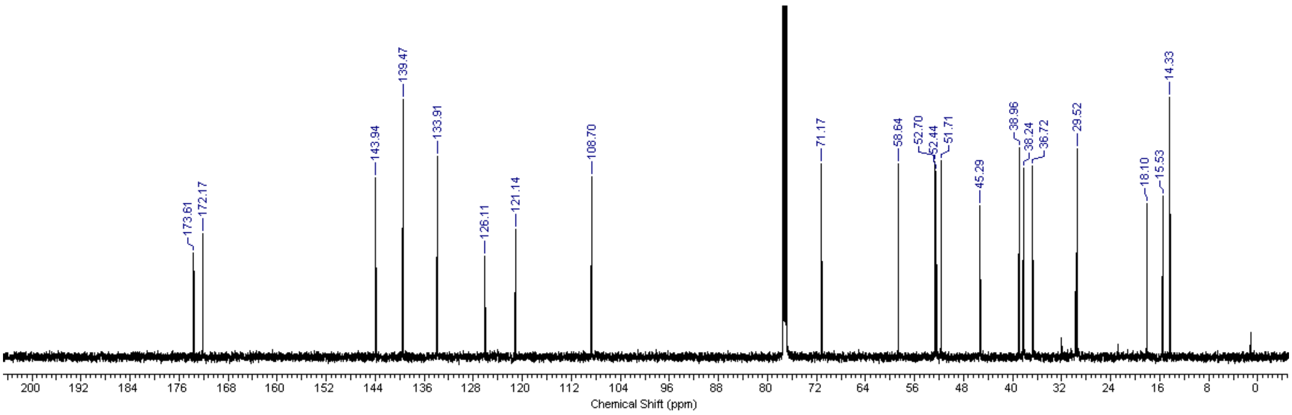

4.22 Compound *rac*-8

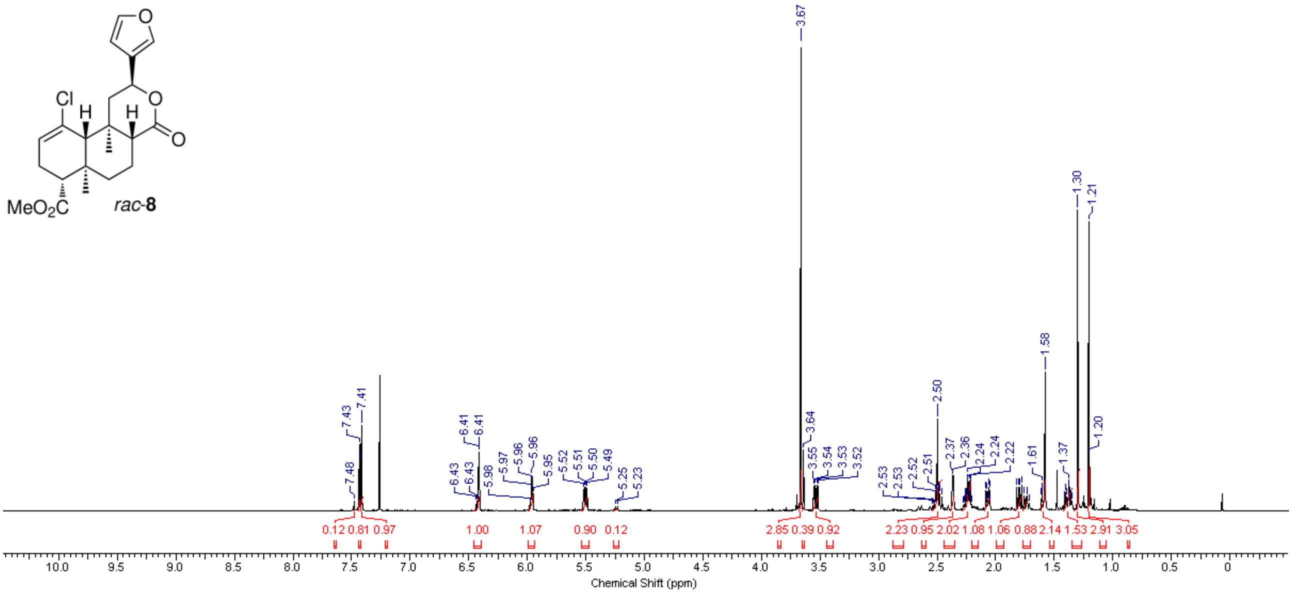

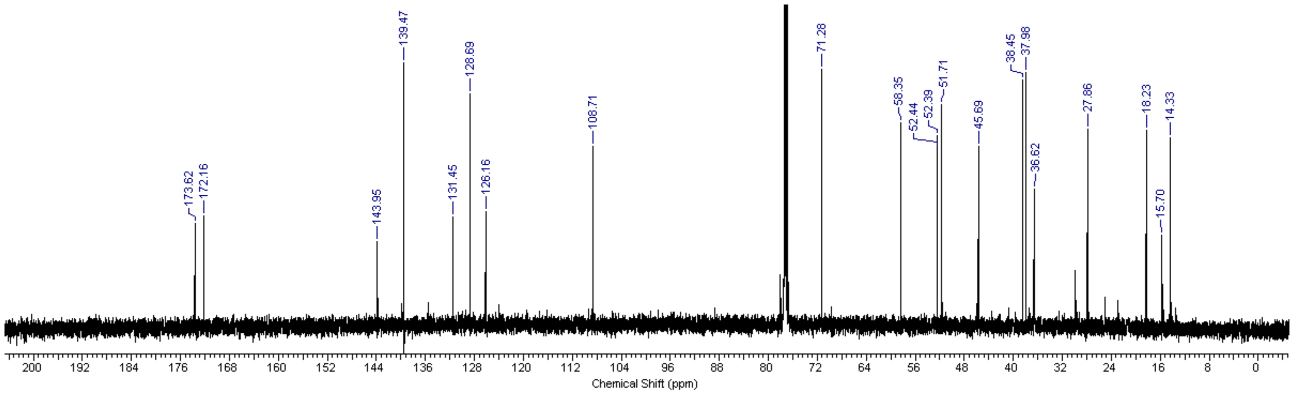

4.23 Compound *rac*-39

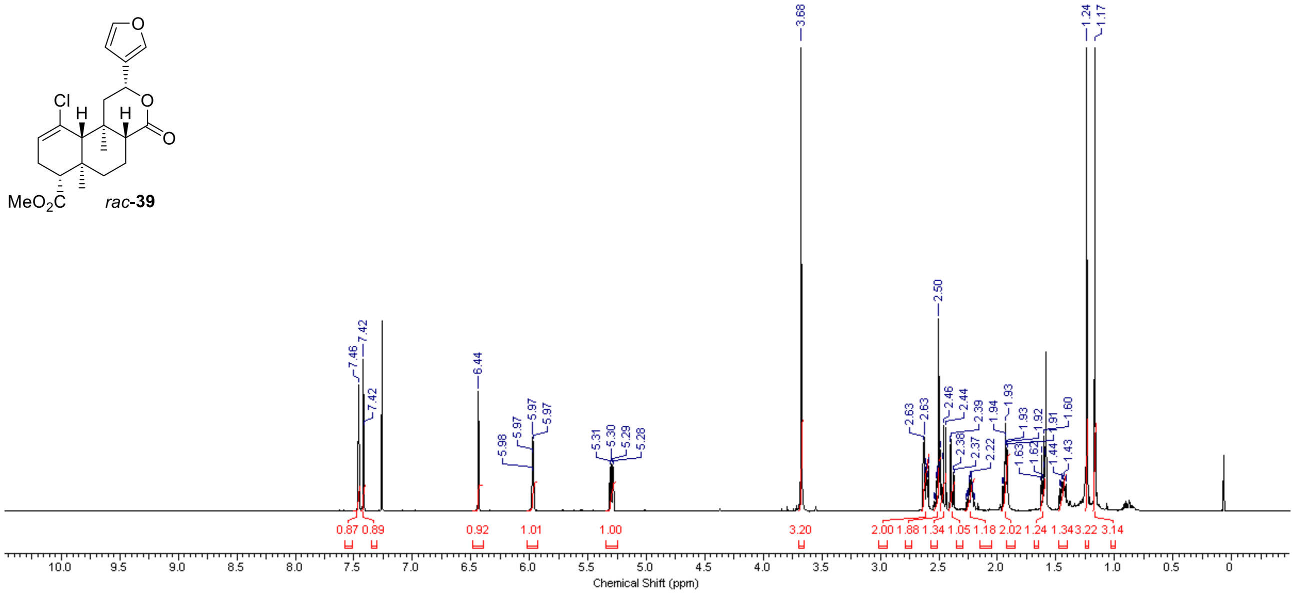

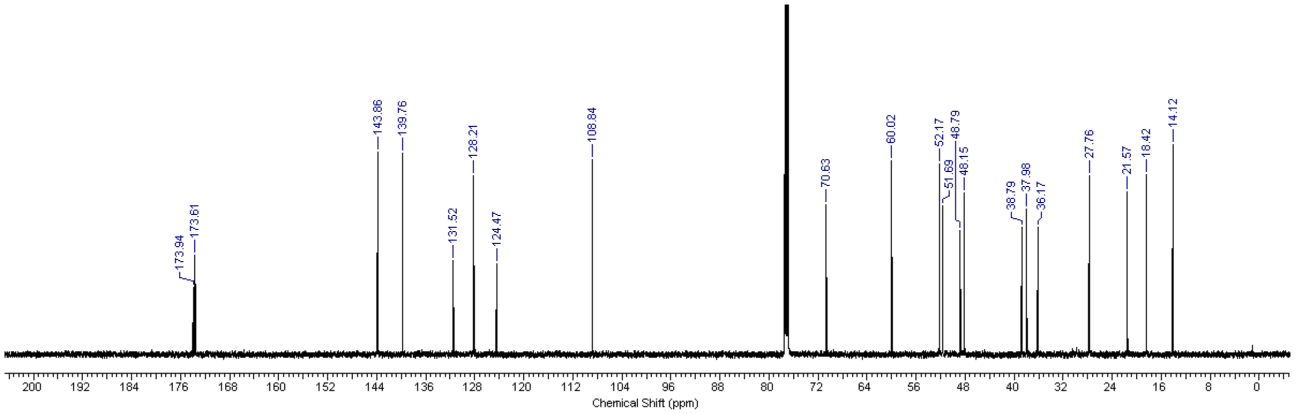

4.24 Compound 15

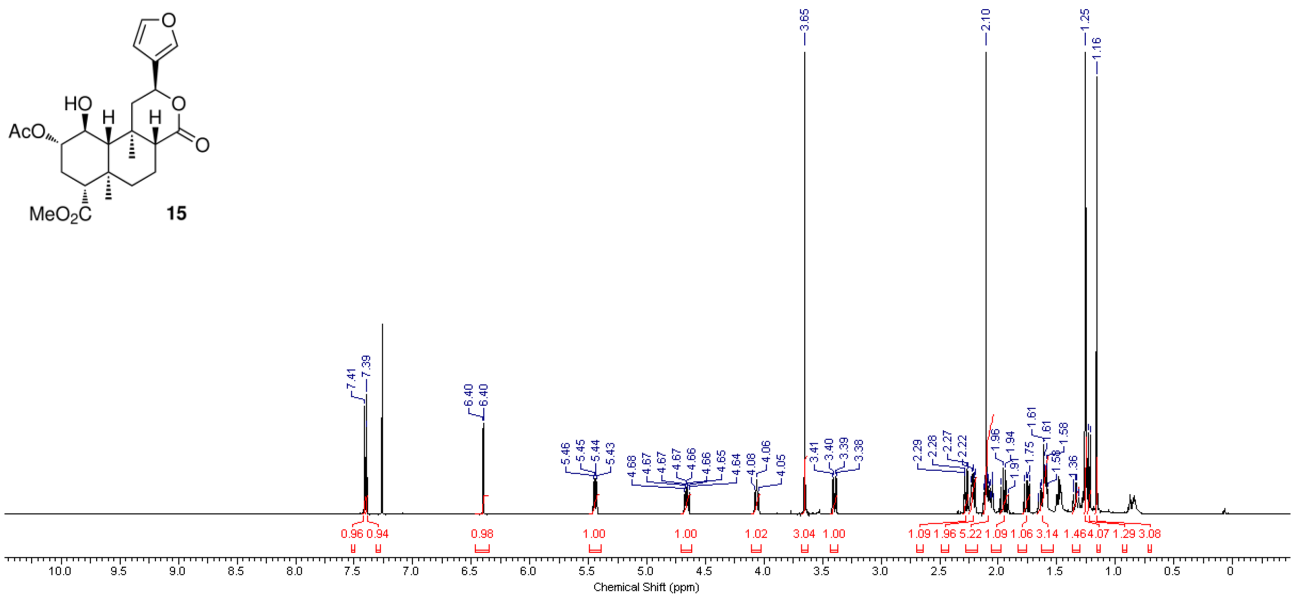

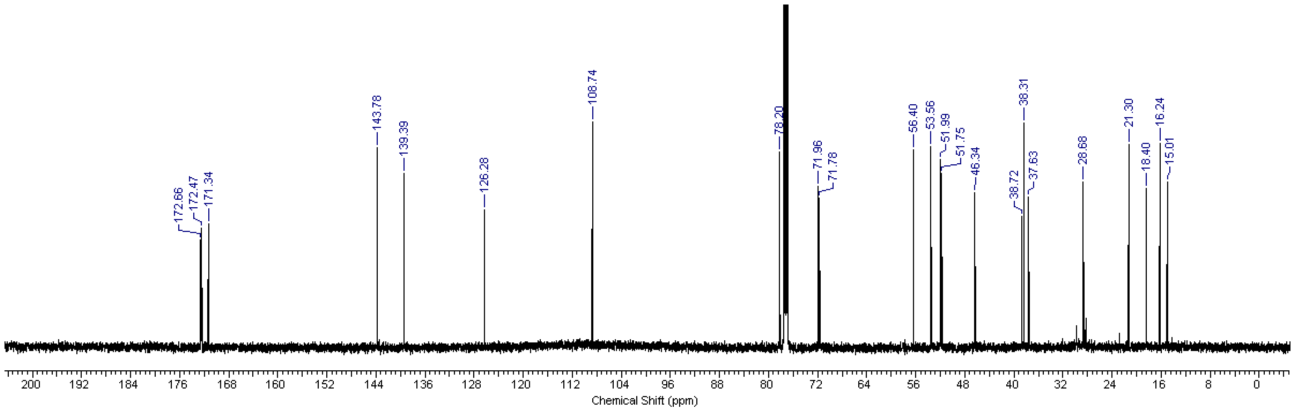

4.25 Compound 1

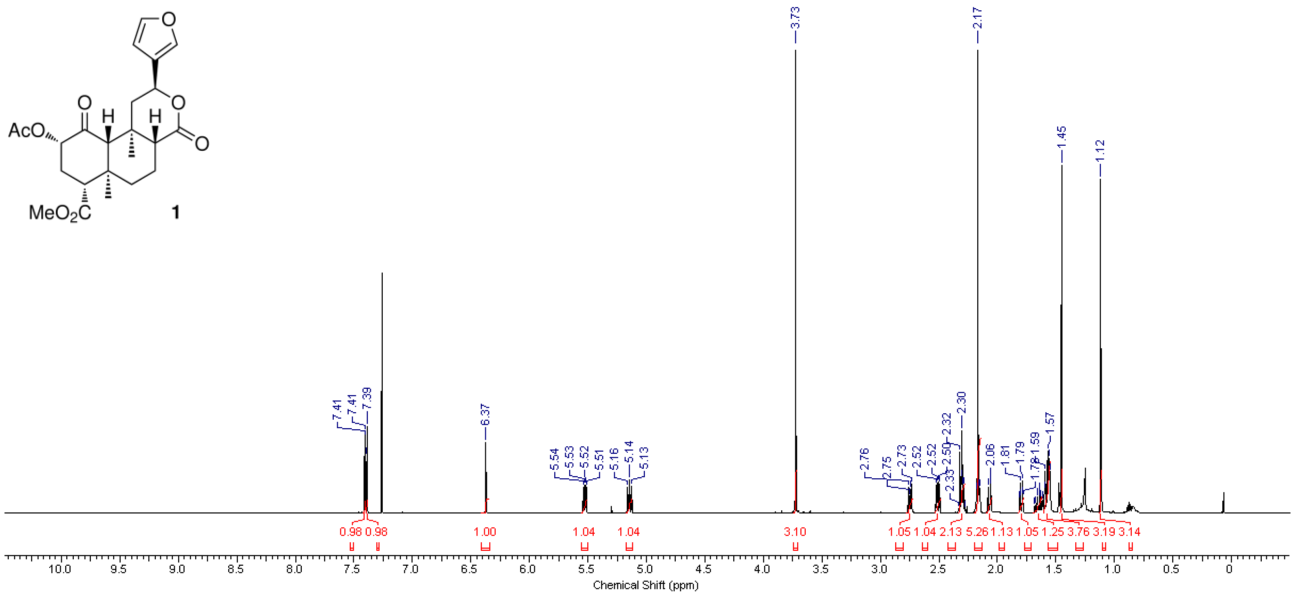

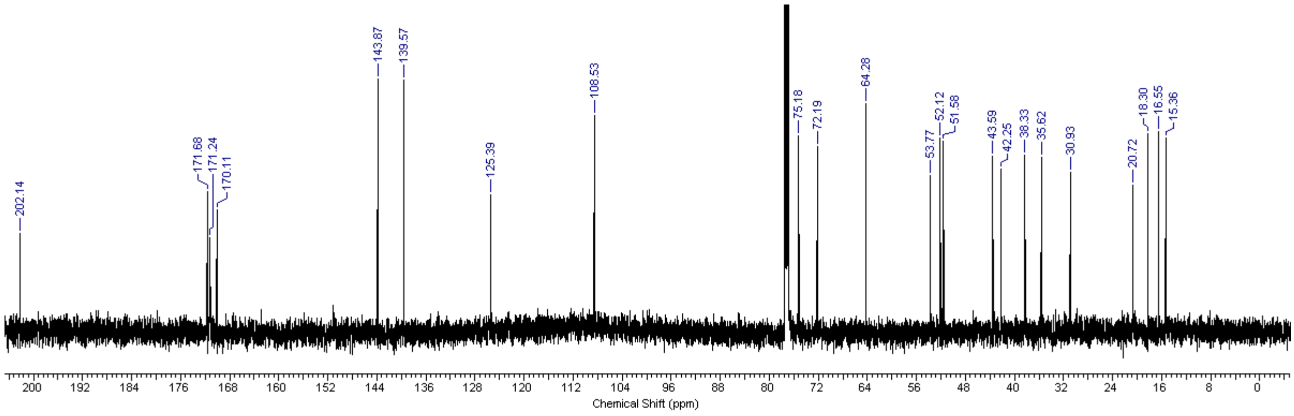
202.14
171.68
171.24
170.11
143.87
139.57
125.39
108.53
75.18
72.19
64.28
53.77
52.12
51.58
43.59
42.25
38.33
35.62
30.93
20.72
18.30
16.55
15.36
200 192 184 176 168 160 152 144 136 128 120 112 104 96 88 80 72 64 56 48 40 32 24 16 8 0
Chemical Shift (ppm)
